# Cancer of childhood in sub-Saharan Africa

**DOI:** 10.3332/ecancer.2017.755

**Published:** 2017-07-28

**Authors:** Cristina Stefan, Freddie Bray, Jacques Ferlay, Biying Liu, D Maxwell Parkin

**Affiliations:** 1 Medical Research Council, PO Box 19070, Tygerberg 7505, Republic of South Africa; 2 Section of Cancer Surveillance, International Agency for Research on Cancer, Lyon, France; 3 African Cancer Registry Network, INCTR, Prama House, 267 Banbury Road, Oxford OX2 7HT, UK; 4 CTSU, University of Oxford, Oxford OX3 7LF, United Kingdom

**Keywords:** cancer, incidence, childhood, sub-Saharan Africa, cancer registry

## Abstract

Measurement of incidence rates of childhood cancer in Africa is difficult. The study ‘Cancer of Childhood in sub Saharan Africa’ brings together results from 16 population-based registries which, as members of the African Cancer Registry Network (AFCRN), have been evaluated as achieving adequate coverage of their target population. The cancers are classified according to the third revision of the International Classification of Childhood Cancer (ICCC-3) and recorded rates in Africa are compared with those in childhood populations in the UK, France, and the USA.

It is clear that, in many centres, lack of adequate diagnostic and treatment facilities leads to under-diagnosis (and enumeration) of leukaemias and brain cancers. However, for several childhood cancers, incidence rates in Africa are higher than those in high-income countries. This applies to infection-related cancers such as Kaposi sarcoma, Burkitt lymphoma, Hodgkin lymphoma and hepatocellular carcinoma, and also to two common embryonal cancers - retinoblastoma and nephroblastoma. These (and other) observations are unlikely to be artefact, and are of considerable interest when considering possible aetiological factors, including ethnic differences in risk (and hence genetic/familial antecedents).

The data reported are the most extensive so far available on the incidence of cancer in sub Saharan Africa, and clearly indicate the need for more resources to be devoted to cancer registration, especially in the childhood age range, as part of an overall programme to improve the availability of diagnosis and treatment of this group of cancers, many of which have—potentially—an excellent prognosis.

## 1. Foreword

There is an increasing global awareness of the growth of the cancer epidemic that will clearly become stronger in the coming decades. For many in the healthcare system, it has become obvious that, even in the richest of countries, it is now impossible to pay for all cancer treatments for all people and that environmental and lifestyle causes of malignant disease have to be controlled if we want to keep it in check. But in this struggle, we should not lose sight of the few cancers appearing in childhood.

There are few known environmental or lifestyle causes of childhood cancer; therefore, prevention is not meaningful. But with the right treatment, the rates of cure can be as high as 80%, as has been demonstrated in high-income populations. Early diagnosis of some cancers – for example retinoblastoma – can markedly improve prognosis. This kind of success should make childhood cancer a ‘low-hanging fruit’ to be picked first in the fight against the disease. This is, however, not the case in many low resource countries. The fact that only between 5 and 20 children out of 100,000 will develop malignancies has not helped to attract the attention of the health authorities on this issue.

The African continent illustrates the above situation very clearly: There are still too few dedicated children’s cancer centres on the continent, too few paediatric oncologists and specialised nurses, sick children are diagnosed late, and there are comparatively many more deaths here than elsewhere in the world. In order to fight efficiently for African children with cancer, a first step would be to create awareness of the magnitude and the characteristics of the problem.

This necessary awareness is provided by the cancer registries. In Africa, only South Africa operates a national childhood cancer registry. In other countries, however, regional population-based or hospital-based registries exist for many years. Presently, they are members of the African Cancer Registry Network (AFCRN), which has become, with international support, a regional hub for cancer registration in sub-Saharan Africa.

This monograph on childhood cancer statistics in sub-Saharan Africa contains information from 16 registries. It contributes to the knowledge about children’s cancer in sub-Saharan Africa, presenting data directly from source. This approach distinguishes it from the national estimates of cancer incidence in Globocan, prepared by the International Agency for Research on Cancer (IARC) which, due to the more limited data sets available requires extrapolation the statistic findings from one region to the surrounding ones. The result will be, obviously, that figures will differ from those produced by IARC.

It is, nevertheless, highly necessary to make known – to the scientific community and to the political factors of decision on health care – these data, which at the moment represent the best image of the malignant disease in children in the respective regions. Furthermore, a first essential step is to use the data as they exist now in order to drive continued improvement in cancer intelligence in the continent in the future. I am convinced that this monograph is a significant step forward towards improving the care for childhood cancer.

**Dr D Cristina Stefan**

Vice President, Medical Research Council, Republic of South Africa

## 2. Contributors

The following have agreed to the publication of the results for their cancer registry in the monograph, and have edited and approved the text describing their registry, and the results in the tabulations in Chapter 6.

**Table:** 

Eastern Africa	
Ethiopia, Addis Ababa	M.A. Woldegeorgis, S.A. Endalew, T.G. Gaga, Addis Ababa City Cancer Registry, School of Medicine, Addis Ababa University, Ethiopia, Tel: +251 911240621; E-mail: aaccrregistry@gmail.com
France, Reunion	E. Chirpaz, Cellule Epidémiologique, Prévention et Education pour la Santé, 12, rue Jean Chatel, 97400 St Denis, Reunion, Tel: +262 203821; Fax: +262 219323; E-mail: emmanuel.chirpaz@chr-reunion.fr
Kenya, Eldoret	N. Buziba, G. Chesumbai, Eldoret Cancer Registry, Moi University School of Medicine, Department of Haematology Blood Transfusion, PO BOX 4606 30100, Eldoret, Kenya, Tel: +254 53 2033461; Email: eldoretcancerregistry@gmail.com
Kenya, Nairobi	A.R. Korir, R. Gakunga, N. Okerosi, Nairobi Cancer Registry, Kenya Medical Research Institute Centre for Clinical Research, P.O. Box 20778, 00202, Nairobi Kenya, Tel: +254 020-2722541; Fax: +254 020-2720031; E-mail: cancerregistry@kemri.org
Malawi, Blantyre	C. Dzamalala, S. Kachiwala, Malawi Cancer Registry, Queen Elizabeth Central Hospital, P.O. Box 95, Blantyre Malawi, Tel: +265 1878058; Fax: +265 8714700; E-mail: mcr@afcrn.org
Mauritius	S.S. Manraj, Mauritius National Cancer Registry, Central Health Laboratory, Mauritius, Tel: +230 2433772/425 7118; Fax: +230 424 5848; E-mail: ssmanraj@gmail.com
Uganda, Kyadondo County	H.R. Wabinga, S. Nambooze, Department of Pathology, College of Health Sciences, Makerere University PO Box 7072, Kampala Uganda, Tel: +256 41531730; Fax: +256 41530412; E-mail: kampalacancerregistry@gmail.com
Zimbabwe, Harare: African	E. Chokunonga, M. Borok, Parirenyatwa Hospital, P.O. Box A 449, Avondale, Harare Zimbabwe,Tel: +263 4791631 ext 152; Fax: +263 4794445; E-mail: cancer@ecoweb.co.zw
**Southern Africa**	
Botswana	M. Kebabonye-Pusoentsi, H.G. Medhin, Botswana National Cancer Registry (BNCR), Ministry of Health, Department of Public Health, Non Commnicable Disease Programme, PO Box 1373, Gaborone BotswanaTel: +267 72306355/3632444; E-mail: mpusoentsi@gmail.com
South Africa Rep., South Africa Childhood Cancer Study Group	C Stefan, Faculty of Medicine and Health Sciences, University of Stellenbosch, PO Box 241; Francie van Zijl Drive, CAPE TOWN 8000, South Africa; E-mail: cristinastefan10@gmail.com
South Africa Rep., Eastern Cape	N. Somdyala, Burden of Disease Research Unit, South African Medical Research Council, PO Box 19070, Tygerberg, South Africa Republic, Tel: +27 (0) 21 938 0314; Fax +27 (0) 21 938 0310; E-mail: nontuthuzelo.somdyala@mrc.ac.za
**Western Africa**	
The Gambia	R. Njie, B. Lamin, Gambia Cancer Registry, Hepatitis Unit, Medical Research Council, The Gambia Unit, Atlantic Road, Fajara PO Box 273, National Cancer Registry, Gambia, W/Africa, Tel: +220 4495 442 dial extension 5004; E-mail: lbojang@mrc.gm
Guinea, Conakry	M. Koulibaly, Registre de Cancer de Guinee, Centre National d’Anatomie Pathologique, Faculté de Médecine/Pharmacie, Université de Conakry, B.P. 4152, Conakry, Tel: +224 628 33 30 61; E-mail: mtoty09@gmail.com
Mali, Bamako	S. Bayo, B. Kamate, C. Traore, I. Malle, Registre du Cancer du Mali, Département de Pathologie, Hôpital National du Point G, Bamako Mali, Tel: (223) 222-4231 / 222-0739; Fax: (223) 221-1999; E-mail: bayosine@yahoo.fr
Niger, Niamey	H. Nouhou, Chef du laboratoire d’anatomie et cytologie pathologiques, Faculté des Sciences de la SantéB.P: 10896 Niamey Niger, Tél: +227220315727/20315730; Fax: +22720315730; E-mail: hnouhou@yahoo.fr
Nigeria, Ibadan	O.J. Ogunbiyi, Department of Pathology, University College Hospital, P.M.B. 5116, Ibadan Nigeria Tel: +234 802 3231728; Fax: +234 809 4654000; E-mail: fogunbiyi@comui.edu.ng

## 3. Acknowledgments

The most important acknowledgment is naturally to all of the contributing registries, listed in Chapter 2. The directors of the registries consented to the use of their data, stored in the database of the African Network of Cancer Registries, and to the publication of the resulting analyses. We acknowledge also the dedicated work of the registry staff (including the unpaid interns and volunteers) who collected the data – often in difficult and trying circumstances – and coded and entered it into the local databases, which contribute to the central AFCRN archive.

Coordination of the project was undertaken within the framework of the activities of the African Cancer Registry Network (AFCRN). AFCRN is a project of the Cancer Registry Programme of the International Network for Cancer Treatment and Research (INCTR). It is supported financially through The (INCTR) Challenge Fund, a registered UK charity (charity number 1079181) that raises funds for INCTR projects. The Challenge Fund in turn receives donations designated to support cancer registry activities in low- and middle-income countries^1^. For this project, in particular, the support of the South African Medical Research Council, via a Funding Agreement (March 2015) is gratefully acknowledged.

Within IARC, Mr Sebastien Antoni of the Section of Cancer Surveillance provided support in the organisation of the data from the cancer registries in the African Network of Cancer Registries database, from which the tables were prepared.

We would particularly like to thank Dr Ian Magrath, President of the International Network of Cancer treatment and Research (INCTR), and Dr Lorenzo Leoncini, Department of Medical Biotechnology, University of Siena, for their assistance with the discussions of childhood leukaemia and lymphoma, and for providing much of the text related to Burkitt lymphoma aetiology and pathogenesis. Scott Howard (St Jude Hospital, Memphis) reviewed the section on Retinoblastoma (section 8.5).

## 4. Introduction

Cancer continues to receive a relatively low public health priority in Africa, largely because of limited resources and other pressing public health problems, including communicable diseases such as acquired immunodeficiency syndrome (AIDS)/human immunodeficiency viral (HIV) infection, malaria, and tuberculosis. Another factor may be a general lack of awareness among policy makers, the general public, and international private or public health agencies, concerning the magnitude of the current and future cancer burden on the continent and its economic impact. Cancers in childhood have received very little attention, for the same reasons, yet the probability of developing a cancer in the first 15 years of life varies relatively little throughout the world [1].

Most publications from Africa on the nature and pattern of childhood cancer describe case series, from pathology laboratories, referral hospitals, or specialist departments within them. Although these can provide useful insights when there are glaring anomalies in the observed pattern, in general, it is difficult to use such data for comparative studies, in part because the absolute risk of the disease in the population (in terms of the incidence rate) is not known, and in part because of the selective factors operating in a given cases series. Thus, clinicians can only document the cases that are referred to them, and pathologists rely upon biopsies being performed, with the inevitable bias in the composition of the case series.

It is only through meticulous collection of data on every new case arising in a given population of known size and composition (by age and sex, at least) that the true picture can be obtained. This can only be achieved by population-based cancer registration. Cancer registration in Africa has a long history, but it has proved to be a difficult task, partly due to logistical reasons (difficulty in tracing cases, and lack of medical information systems), and partly due to woeful underfunding. However, in recent years, there has been an effort to improve the quality and scope of cancer registration, through the Global Initiative on Cancer Registration (GICR) of the International Agency for Research on Cancer (IARC). In sub-Saharan Africa, there are at least 25 population-based cancer registries that achieve a reasonable degree of population coverage, from which valid data on cancer incidence can be obtained[2].

Nevertheless, studies of the incidence of cancer in childhood in Africa are even more difficult than those of adults. Because of the relative rarity of cancer in this age group, population-based studies must involve rather large populations (or long time periods) in order that sufficient cases can be assembled to permit calculation of valid rates. This is the major reason that there are so few population-based studies.

Pooled results, for the period 2001–2010, from six of the registries appearing in this monograph, from the *International Incidence of Childhood Cancer*, Volume III, have recently been published [3], and these data, plus those from more restricted periods, have appeared online (http://iicc.iarc.fr/results/registries.php). Older data were published in IICC-2 [4], and in a section of the monograph Cancer in Africa, also published by IARC [5].

In this volume, we have therefore obtained data from functioning population-based cancer registries in sub-Saharan Africa, all of which, as members of the African Cancer Registry Network have been evaluated as achieving at least 70% coverage of their target population (i.e. identifying 70% of the actual incident cancers overall). The original objective was to include data from those registries that could provide at least 150 paediatric cancer cases, in a recent period of no longer that 10 years duration. At the time, the study began, 15 cancer registries met this criterion, and agreed to participate. One smaller registry (Eastern Cape, Rep. of South Africa) was also included. Table 4 lists the participants, with the total number of childhood cancers contributed by each one.

The editors hoped that the data in this volume will help to highlight (or confirm) some of the interesting features of the epidemiology of paediatric cancer in sub-Saharan Africa and act as a stimulus to further research, as well as drawing attention to the needs of this important, but neglected, group of patients.

## 5. Sources and methods

### Source of the data

The data used to create the tables presented in this monograph were extracted from the database of the African Cancer Registry Network. The objective was to process childhood cancer registrations for a period of 10 years, as recent as possible, for which the number of cases registered annually was reasonably constant.

### Processing of the data

A listing of individual anonymous cases with the following variables was extracted for each contributor:

1. a registration number which identifies the patient or the case
2. sex
3. ethnic group or race (optional)
4. age at incidence date (or date of birth)
5. date of incidence
6. site of the tumour
7. morphology of the tumour
8. behaviour of the tumour
9. basis of diagnosis

Cases with an age at diagnosis (in years) between 0 and 14 were abstracted.

All data had been coded according to the ICD-O3 [6]. They were processed by the IARC software packages DEPedits and IARCcrgTools [7] for verification. Since almost all contributing registries used the CanReg system, a software program developed at IARC and designed for population-based cancer registries, the data had already been submitted to the same edits as those performed by the IARCcrgTools programs. This simplified and speeded up the data validation process.

From the ICD-O3 codes, each case was classified according to the third revision of the International Classification of Childhood Cancer (ICCC-3) [8]. This categorises the cancers into 12 main groups, which are split further into 47 subgroups (Table 5.1). Benign tumours (behaviour code 0 in ICD-O3) and those of uncertain behaviour (code 1) were excluded, since African registries do not systematically record such tumours. Because of the limited numbers of cases in most of the series in this monograph, we present detailed tabulations for a limited number of subgroups (Table 5.2).

### Presentation of the data

The main sets of tables in this book present data on age-specific and age-standardised incidence, either by population (cancer registry) or as summary tables by cancer site.

### Tables for each registry:

#### Population at-risk

The AFCRN database contains data for each registry on population at risk by sex and age group for as many years as possible. A denominator corresponding to the period of the incident cases (person-years at risk) was estimated based on this information, with intercensal estimates and postcensal projections, as necessary. The average annual population at risk (by five-year age group) for the period analysed is presented as a table, within the description of the registry.

#### The cancer incidence table

An example is shown as Table 5.2.

The left-hand side of the table shows the number of cancer cases registered during the corresponding period by cancer type, and age group.

In the column *M/F,* the sex ratio (number of cases in boys, divided by the number in girls) is shown.

The relative frequency of the different cancer types (as a percentage of the total) is shown in the column labelled REL. FREQ. (%).

The right-hand side of the table shows the incidence rates, per million person-years. Incidence rates are presented for each 5-year age group (0–4, 5–9, 10–14), and for all ages (0–15) – the Crude rate.

In the column Cum., 0–14 is the cumulative incidence rate up to age 14 years. This is the sum over each year of age of the age-specific incidence rates, taken from birth to age 14.

Thus, if the rates at ages 0–4, 5–9, 10–14 are denoted as *r*_1_*, r*_2_*, r*_3_, then Cum. 0–14 = 5x(*r*_1_*+ r*_2_*+ r*_3_).

Note that the final row of the table includes ‘Other and Unspecified Cancers’. These include not only cases in Group XII of ICCC-3 (almost all of which are cases with unspecified histology – ICD-O code 8000/3), but also a few diagnoses that are not listed separately in the table, such as IVb, VIb, and VIc.

#### Summary tables

The tables which appear in Chapter 8 (Results and Discussion – by type of Cancer) show the results for each cancer registry, for a specific cancer type.

For each registry is displayed:

- The number of cases,
- The sex ratio (number of cases in boys, divided by the number in girls)
- The crude incidence rate (per million)
- The age-standardised rate (and standard error) per million
- The cumulative rate 0–14 (and standard error) per million.

The age-standardised rate (ASR) is calculated, using the age-specific rates (*r*_1_*, r*_2_*, r*_3_) and the world standard population, for the three age groups 0–4, 5–9, and 10–14:

ASR = [(*r*_1_ × 12000) + (*r*_2_ × 10000) + (*r*_3_ × 9000)]/31000.

The standard errors of the ASR and cumulative rate are calculated as in Boyle & Parkin (1991) [9].

For comparative purposes, the results for four other populations are shown. They are as follows:

- USA (18 cancer registries of the SEER programme) white children
- USA (18 cancer registries of the SEER programme) black children
- United Kingdom
- France (10 cancer registries)

The results are for 2003–2007, and the relevant data were extracted from the database of the SEER program (https://seer.cancer.gov/analysis/incidence.html), and for the United Kingdom and France, from the database of ‘Cancer Incidence in Five Continents, Volume X’ [10]. As for the African series, tumours of benign and uncertain behaviour were not included.

For each cancer type, the cumulative rates for the contributing registries (and for the comparison populations) are also shown in the form of a bar chart. The bars for the registries from Eastern Africa are coloured red, those for Western Africa green, those for Southern Africa blue, and for the comparison populations, in grey. For United Kingdom and France, it was not possible to calculate cumulative rates for those paediatric cancers (neuroblastoma, retinoblastoma, nephroblastoma) that are not defined by ICD-10 codes or the morphological groups included in cancer incidence in five continents.

### Data quality

There are few indicators of data quality that are appropriate to evaluating the completeness and validity of registration of childhood cancers. Civil registration of deaths (by cause) is little developed in sub-Saharan Africa, so that the ratio of deaths in the population to cases registered cannot be used. In ‘Cancer Incidence in Five Continents’ [10], the possibility of underenumeration (and duplicate registration) in this age range is investigated by comparing incidence rates within the childhood age groups with the corresponding values from the previous volume (Volume IX) (Table 5.3). The rationale is that the incidence rates of cancer (all types combined) within children (i.e. within the age groups 0–4 years, 5–9 years, and 10–14 years) tend to exhibit much less variability than do the incidence rates of cancer in adults. However, the editors note that ‘there are some well-documented geographical and ethnic differences for certain childhood cancers’ which may rather invalidate this approach, and it is known that, in particular, there are some important and major variations in incidence rates for some childhood cancers in African populations [5].

In Table 5.4, we show for each contributing centre the percentage of cases, within the major diagnostic groups, diagnosed on the basis of histology or cytology (morphologically verified). Although this is a useful indicator of validity (and to some extent of completeness of registration) [11], it is probably of lesser value in childhood than in adults, since a large proportion of cancers in childhood (with the exception of tumours of the nervous system) would normally be diagnosed by invasive techniques.

The percentage of cases for which the diagnosis is based only on information from a death certificate (DCO%) also appears as an indicator of data validity in cancer incidence in five continents. Unfortunately, this has less widespread application as a comparative measure in Africa, since several registries do not have access to death certificates, or decline to register unverified diagnoses of ‘cancer’, having observed the poor accuracy of cause of death statements. In Table 5.5, we show the percentage of death certificate registrations in childhood from the 16 registries, and also the percentage of DCO cases among the ‘Other and Unspecified cancers’, or with a more specific diagnosis. Only two centres (Eldoret and Harare) have substantial numbers of DCO cases, almost all of which are in the ‘Other and Unspecified’ category.

**Table 5.5.** Cancer cases registered from death certificate only: all cases, those of specified type (ICCC groups I–IX, and other and unspecified cancers.

	All cases		Specified cancers (I–XI)	Other and unspecified
	Number	DCO%	DCO%	DCO%
**Africa, East**				
Ethiopia, Addis Ababa 2011–2013	**160**	**0**	**–**	**–**
Kenya, Eldoret 2007–2011	**282**	**11.0**	**5.3**	**85.0**
Kenya, Nairobi 2007–2012	**577**	**2.9**	**2.2**	**13.9**
Malawi, Blantyre 2003–2010	**986**	**0**	**–**	**–**
Uganda, Kyadondo County 2003–2012	**1254**	**0**	**–**	**–**
Zimbabwe, Harare: African 2003–2012	**504**	**6.0**	**4.9**	**40.0**
France, Reunion 2002–2008, 2011	**178**	**1.1**	**0.6**	**25.0**
Mauritius 2003–2012	**270**	**0**	**–**	**–**
**Africa, South**				
Botswana 2003–2008	**267**	**0**	**–**	**–**
RSA 2008–2012	**3483**	**0.1**	**0.1**	**–**
RSA: Black 2008–2012	**2583**	**0**	**–**	**–**
RSA: White 2008–2012	**340**	**0.3**	**0.3**	**–**
RSA: Eastern Cape 2003–2012	**113**	**0**	**–**	**–**
**Africa, West**				
The Gambia 2002–2011	**194**	**0**	**–**	**–**
Guinea, Conakry 2001–2010	**166**	**0**	**–**	**–**
Mali, Bamako 2006–2014	**801**	**1.0**	**1.1**	**0.6**
Niger, Niamey 2001–2009	**175**	**0**	**–**	**–**
Nigeria, Ibadan 2003–2012	**387**	**0**	**–**	**–**

**Figure:**
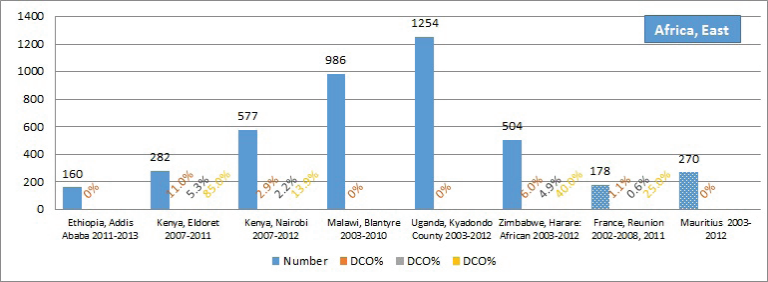

**Figure:**
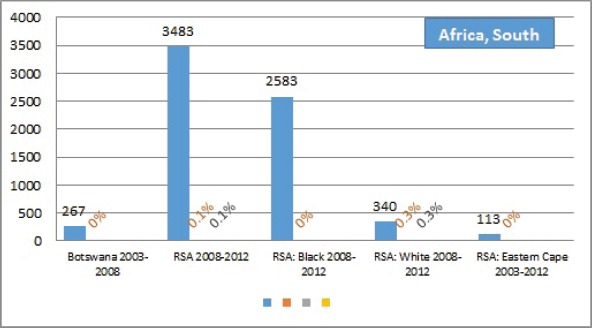

**Figure:**
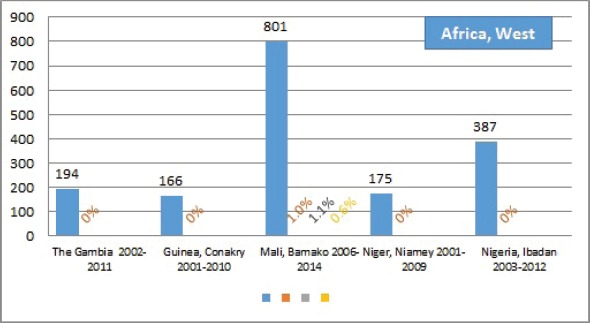

## 6. Results by registry (by region)

### 6.1. East Africa

#### 6.1.1 Ethiopia: Addis Ababa City Cancer Registry

**Figure:**
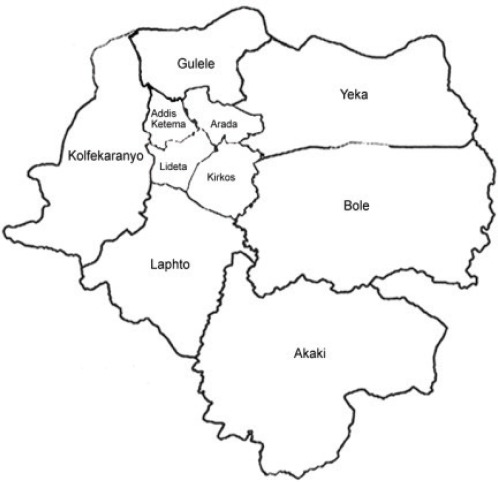
Map of the catchment area served by the cancer registry. Source unknown.

The Addis Ababa City Cancer Registry is the first population based cancer registry in Ethiopia. It was established in 2011 in the Radiotherapy Centre of Tikur Anbessa Specialised Hospital, Addis Ababa University. The registry aims to collect information on all cancer cases occurring in the city of Addis Ababa (population in 2012 estimated as 3.05 million).

The main sources of information for the registry are as follows:

1. Hospitals that include departments of radiotherapy, pathology, haematology, paediatrics, gynaecology, surgery, internal medicine, etc.
2. Medical treatment clinics
3. Diagnostic laboratories with pathology and haematology services.

Because of the weak infrastructure of medical information systems, data collection is entirely active, with contact persons working in each of the institutions providing cases paying regular visits to clinical and laboratory services to identify and record new cases. The largest number of the registrations (54.7%) came from Tikur Anbessa Specialised Hospital. The other 45.3% is from other two government and 14 private health institutions.

There is a paediatric oncology department at the Tikur Anbessa Specialised Hospital. Two paediatric oncologists attend the patients.

The registry uses the Canreg5 system for data entry, data analysis and management.

#### Years presented

Data for the five-year period 2011–2013 are presented in the following table.

**Table 6.1.1.** Ethiopia, Addis Ababa (2011–2013).

	NUMBER OF CASES				REL. FREQ.(%)		RATES PER MILLION					
	0-4	5-9	10-14	All	*M/F*	Overall	0-4	5-9	10-14	Crude	Cum. 0-14	%MV
I Leukaemia	14	19	16	**49**	*1.5*	30.6	27.3	35.0	24.7	28.8	**434.9**	93.9
Ia Lymphoid	11	18	9	**38**	*1.5*	23.8	21.5	33.1	13.9	22.3	**342.4**	94.7
Ib Acute myeloid	1	0	3	**4**	*3.0*	2.5	2.0	-	4.6	2.3	**32.9**	100.0
II Lymphoma	8	12	12	**32**	*1.3*	20.0	15.6	22.1	18.5	18.8	**281.1**	93.8
IIa Hodgkin	1	7	8	**16**	*1.0*	10.0	2.0	12.9	12.4	9.4	**135.9**	100.0
IIb NHL (except Burkitt)	5	4	4	**13**	*1.6*	8.1	9.8	7.4	6.2	7.6	**116.4**	92.3
IIc Burkitt	1	0	0	**1**	*-*	0.6	2.0	-	-	0.6	**9.8**	100.0
III Brain and spinal neoplasms	2	1	2	**5**	*1.5*	3.1	3.9	1.8	3.1	2.9	**44.1**	40.0
IVa Neuroblastoma	1	4	0	**5**	*4.0*	3.1	2.0	7.4	-	2.9	**46.5**	40.0
V Retinoblastoma	5	3	0	**8**	*0.6*	5.0	9.8	5.5	-	4.7	**76.4**	62.5
VIa Nephroblastoma	12	1	2	**15**	*0.9*	9.4	23.4	1.8	3.1	8.8	**141.7**	73.3
VII Hepatic tumours	0	0	0	**0**	-	-	-	-	-	-	-	-
VIIa Hepatoblastoma	0	0	0	**0**	-	-	-	-	-	-	-	-
VIIb Hepatic carcinomas	0	0	0	**0**	-	-	-	-	-	-	-	-
VIII Bone tumours	0	1	4	**5**	*1.5*	3.1	-	1.8	6.2	2.9	**40.1**	80.0
VIIIa Osteosarcoma	0	1	2	**3**	*0.5*	1.9	-	1.8	3.1	1.8	**24.6**	66.7
VIIIc Ewing	0	0	2	**2**	-	1.2	-	-	3.1	1.2	**15.4**	100.0
IX Soft tissue sarcomas	6	9	8	**23**	*1.3*	14.4	11.7	16.6	12.4	13.5	**203.1**	95.7
IXa Rhabdomyosarcoma	4	2	2	**8**	*0.6*	5.0	7.8	3.7	3.1	4.7	**72.9**	87.5
IXc Kaposi sarcoma	0	0	1	**1**	-	0.6	-	-	1.5	0.6	**7.7**	100.0
X Germ cell tumours	1	1	3	**5**	*0.7*	3.1	2.0	1.8	4.6	2.9	**42.1**	40.0
XI Carcinomas	3	0	6	**9**	*0.5*	5.6	5.9	-	9.3	5.3	**75.6**	100.0
Other and unspecified	1	2	1	**4**	*1.0*	2.5	2.0	3.7	1.5	2.3	**35.9**	75.0
TOTAL	53	53	54	**160**	*1.2*	100.0	103.4	97.5	83.4	93.9	**1421.4**	85.0

Population (average annual)		
	**Males**	**Females**
0–4	86,847	83,998
5–9	87,893	93,303
10–14	94,061	121,842
all (0–14)	268,802	299,143

**Source:** Population estimates for each individual year for the period.

1997: Population and Housing Census.

1998−2012: Projection using Exponential Growth Rate Method.

#### 6.1.2 France: Registre des Cancers de la Réunion

**Figure:**
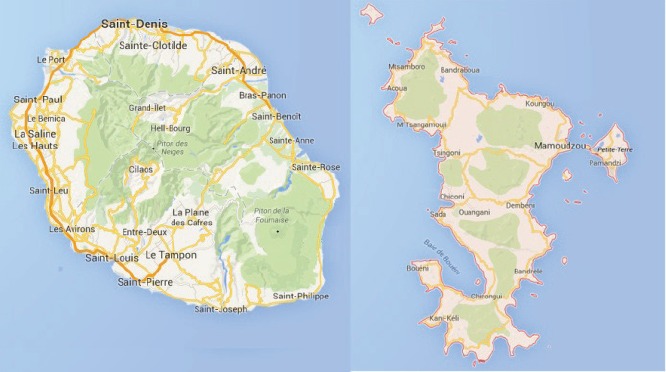
Map of the catchment area served by the cancer registry. Source unknown.

The registry was founded in 1989 and is based in the Clinical Research and Epidemiology Unit of Felix Guyon Hospital and financed by the regional health agency (ARS Réunion – Mayotte).

The registry covers the whole population of Reunion, which is one of four French overseas departments. The population was estimated to be around 850,000 in 2014. It is a mixed and cosmopolitan population (European, African, Malagasy, and Indian origin mainly).

Since 2005, registration has been extended to paediatric cancer cases (age at diagnosis < 18 years old) diagnosed in Mayotte, a small French island. In 2012, year of the last census, the population of Mayotte was estimated to be around 215,000 inhabitants (with 50% aged less than 18).

The registry collects cases from four public hospitals and five private clinics, five of the six histopathology laboratories of the island and the two haematology laboratories located in the University Hospital, the regional cancer network (*ONCORUN*) and Social security. Within these medical services, there are three cancer care units (two public hospitals and one private clinic) including three oncology/haematology units, two radiotherapy centres, one unit specialised in paediatric oncology, and one nuclear medicine unit (scintigraphy and PETSCAN). There is no histopathology laboratory in Mayotte; all analyses are performed in Reunion Island and all paediatric cancers diagnosed in Mayotte are treated in Reunion Island.

Death certificates are not used as a source of information (in France, the part of the certificate that contains the cause of death is anonymised). Registration is active and it is facilitated by the fact that data requested are largely computerised at a lot of levels.

ACCESS® software customised locally is used for data entry and analysis.

#### Years presented

The registry was inactive for some years after 2006, and, although retrospective data collection has taken place, this was incomplete for the years 2009 and 2010. Results for eight years: **2002–2008 and 2011** are therefore presented in the following table, only for Reunion Island.

**Table 6.1.2.** France, Reunion (2002–2008, 2011).

	NUMBER OF CASES				REL. FREQ.(%)		RATES PER MILLION					
	0-4	5-9	10-14	All	*M/F*	Overall	0-4	5-9	10-14	Crude	Cum. 0-14	%MV
I Leukaemia	28	13	12	**53**	*1.8*	29.8	51.4	23.7	21.2	32.0	**481.9**	98.1
Ia Lymphoid	22	11	8	**41**	*1.9*	23.0	40.4	20.1	14.1	24.7	**373.2**	97.6
Ib Acute myeloid	5	2	3	**10**	*1.5*	5.6	9.2	3.7	5.3	6.0	**90.7**	100.0
II Lymphoma	1	5	14	**20**	*0.8*	11.2	1.8	9.1	24.7	12.1	**178.4**	100.0
IIa Hodgkin	0	5	7	**12**	*1.0*	6.7	-	9.1	12.4	7.2	**107.4**	100.0
IIb NHL (except Burkitt)	0	0	5	**5**	*0.7*	2.8	-	-	8.8	3.0	**44.1**	100.0
IIc Burkitt	0	0	1	**1**	*-*	0.6	-	-	1.8	0.6	**8.8**	100.0
III Brain and spinal neoplasms	4	14	6	**24**	*0.8*	13.5	7.3	25.6	10.6	14.5	**217.6**	87.5
IVa Neuroblastoma	14	2	0	**16**	*1.7*	9.0	25.7	3.7	-	9.6	**146.9**	100.0
V Retinoblastoma	6	0	0	**6**	*5.0*	3.4	11.0	-	-	3.6	**55.1**	66.7
VIa Nephroblastoma	6	3	1	**10**	*0.7*	5.6	11.0	5.5	1.8	6.0	**91.3**	100.0
VII Hepatic tumours	3	0	1	**4**	*3.0*	2.2	5.5	-	1.8	2.4	**36.4**	100.0
VIIa Hepatoblastoma	2	0	1	**3**	*-*	1.7	3.7	-	1.8	1.8	**27.2**	100.0
VIIb Hepatic carcinomas	1	0	0	**1**	*-*	0.6	1.8	-	-	0.6	**9.2**	100.0
VIII Bone tumours	1	4	6	**11**	*1.2*	6.2	1.8	7.3	10.6	6.6	**98.7**	81.8
VIIIa Osteosarcoma	0	1	5	**6**	*2.0*	3.4	-	1.8	8.8	3.6	**53.2**	83.3
VIIIc Ewing	1	2	1	**4**	*0.3*	2.2	1.8	3.7	1.8	2.4	**36.3**	75.0
IX Soft tissue sarcomas	8	4	5	**17**	*1.4*	9.6	14.7	7.3	8.8	10.3	**154.1**	100.0
IXa Rhabdomyosarcoma	5	3	4	**12**	*1.4*	6.7	9.2	5.5	7.1	7.2	**108.6**	100.0
IXc Kaposi sarcoma	0	0	0	**0**	*-*	-	-	-	-	-	**-**	-
X Germ cell tumours	0	3	2	**5**	*-*	2.8	-	5.5	3.5	3.0	**45.0**	80.0
XI Carcinomas	1	2	5	**8**	*1.0*	4.5	1.8	3.7	8.8	4.8	**71.6**	100.0
Other and unspecified	1	1	2	**4**	*-*	2.2	1.8	1.8	3.5	2.4	**36.0**	75.0
TOTAL	73	51	54	**178**	*1.3*	100.0	134.1	93.2	95.3	107.3	**1612.9**	94.4

Population (average annual)		
	**Males**	**Females**
0–4	35,004	33,025
5–9	35,017	33,405
10–14	35,819	35,028
All (0–14)	105,839	101,457

**Source:** Data provided by INSEE, based on 1999, 2006, and 2012 censuses.

Intercensal estimates taking into account the natural balance and net migration.

#### 6.1.3 Kenya: Eldoret Cancer Registry

**Figure:**
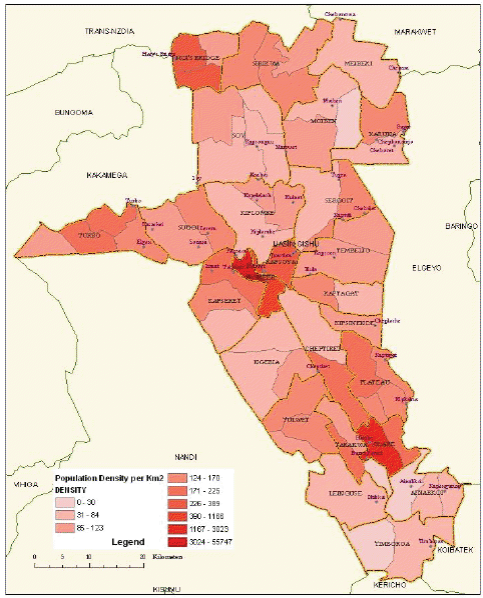
Map of the catchment area served by the cancer registry. Source unknown.

Established in 1999, Eldoret Cancer Registry (ECR) is situated in the Department of Haematology and Blood Transfusion, Moi University College of Health Sciences. It aims to record information on all cancer cases diagnosed among the population of Uasin Gishu County in the Rift Valley region of Kenya, with a population of 894,179 people (2009 Kenyan National Census).

Case finding is an active process. The registry collects all cancer cases that are diagnosed or referred to Moi Teaching and Referral Hospital (MTRH), the second largest hospital in Kenya, receiving patients from the western part of Kenya and beyond. In addition to MTRH, the registry collects data from all government, private hospitals and cancer centres, as well as the Eldoret Hospice. The sources used include the medical records departments, radiotherapy units, haematology and histopathology laboratories, outpatient clinics, medical wards, imaging units and autopsy reports. The ECR also reaches out to clinics of private physicians to collect cases that may have been missed at hospital level.

The ECR has a link with the civil registration office to access cancer-specific mortality data from death certificates. Cases not already in the registry database are traced back to the hospital where the death occurred; those cases that cannot be traced are registered as ‘Death Certificate Only (DCO)’ cases. This results in a substantial percentage of cases being registered as DCO (31, or 11% in 2007–2011), most of which (17) were simply specified as ‘cancer’.

The Moi Teaching and Referral Hospital’s Oncology Division, has a vibrant paediatric oncology unit that is responsible for the treatment and management of paediatrics between the ages 0–14 years.

The registry uses CanReg 5 for data management and analysis.

#### Years presented

Data for the five-year period 2007–2011 are presented in the table below:

**Table 6.1.3.** Kenya, Eldoret (2007–2011).

	NUMBER OF CASES				REL. FREQ.(%)		RATES PER MILLION					
	0-4	5-9	10-14	All	*M/F*	Overall	0-4	5-9	10-14	Crude	Cum. 0-14	%MV
I Leukaemia	14	15	16	**45**	*1.5*	16.0	20.6	23.9	29.3	24.3	**369.0**	93.3
Ia Lymphoid	10	10	8	**28**	*3.7*	9.9	14.7	16.0	14.7	15.1	**226.5**	96.4
Ib Acute myeloid	2	3	3	**8**	*0.6*	2.8	2.9	4.8	5.5	4.3	**66.1**	100.0
II Lymphoma	22	27	32	**81**	*2.9*	28.7	32.3	43.1	58.6	43.7	**670.1**	88.9
IIa Hodgkin	0	2	11	**13**	*2.2*	4.6	-	3.2	20.1	7.0	**116.7**	84.6
IIb NHL (except Burkitt)	18	15	14	**47**	*2.9*	16.7	26.4	23.9	25.6	25.4	**380.1**	89.4
IIc Burkitt	3	6	2	**11**	*2.7*	3.9	4.4	9.6	3.7	5.9	**88.2**	100.0
III Brain and spinal neoplasms	2	3	2	**7**	*0.2*	2.5	2.9	4.8	3.7	3.8	**56.9**	71.4
IVa Neuroblastoma	5	4	0	**9**	*1.2*	3.2	7.3	6.4	-	4.9	**68.6**	88.9
V Retinoblastoma	23	4	1	**28**	*1.0*	9.9	33.8	6.4	1.8	15.1	**209.9**	89.3
VIa Nephroblastoma	9	14	1	**24**	*1.2*	8.5	13.2	22.4	1.8	12.9	**187.0**	95.8
VII Hepatic tumours	1	0	0	**1**	*-*	0.4	1.5	-	-	0.5	**7.3**	100.0
VIIa Hepatoblastoma	0	0	0	**0**	*-*	-	-	-	-	-	**-**	-
VIIb Hepatic carcinomas	1	0	0	**1**	*-*	0.4	1.5	-	-	0.5	**7.3**	100.0
VIII Bone tumours	0	2	7	**9**	*1.2*	3.2	-	3.2	12.8	4.9	**80.1**	77.8
VIIIa Osteosarcoma	0	2	6	**8**	*1.7*	2.8	-	3.2	11.0	4.3	**70.9**	75.0
VIIIc Ewing	0	0	0	**0**	*-*	-	-	-	-	-	**-**	-
IX Soft tissue sarcomas	15	10	15	**40**	*2.1*	14.2	22.0	16.0	27.5	21.6	**327.3**	80.0
IXa Rhabdomyosarcoma	11	3	6	**20**	*1.2*	7.1	16.2	4.8	11.0	10.8	**159.6**	75.0
IXc Kaposi sarcoma	3	5	6	**14**	*3.7*	5.0	4.4	8.0	11.0	7.6	**116.9**	78.6
X Germ cell tumours	0	1	0	**1**	*-*	0.4	-	1.6	-	0.5	**8.0**	100.0
XI Carcinomas	5	4	8	**17**	*1.4*	6.0	7.3	6.4	14.7	9.2	**141.9**	94.1
Other and unspecified	9	5	6	**20**	*1.2*	7.1	13.2	8.0	11.0	10.8	**160.9**	15.0
TOTAL	105	89	88	**282**	*1.6*	100.0	154.2	142.1	161.2	152.2	**2287.2**	83.3

Population (average annual)		
	**Males**	**Females**
0–4	68,684	67,530
5–9	63,190	62,084
10–14	54,540	54,657
all (0–14)	186,414	184,271

**Source:** Uasin−Gishu population, National 2009 Census.

#### 6.1.4 Kenya: Nairobi Cancer Registry

**Figure:**
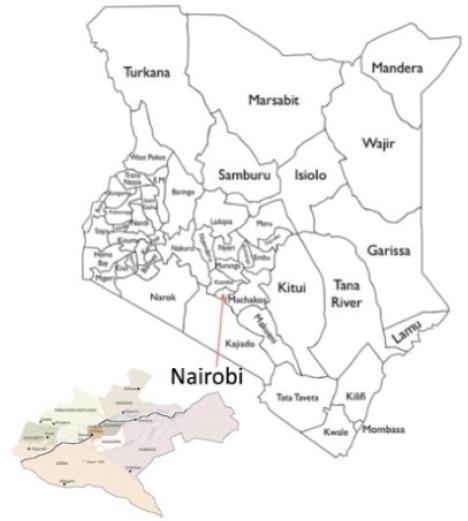
Map of the catchment area served by the cancer registry. Source unknown.

Nairobi Cancer Registry (NCR) is based in Kenya Medical Research Institute (KEMRI), Centre for Clinical Research. KEMRI is mandated by the Government of Kenya to carry out any medical research activities in Kenya on behalf of the Ministry of Health since 1979.

Nairobi County is the smallest of Kenya’s 47 counties and hosts Kenya’s cosmopolitan capital city – Nairobi City. It has a representation of all the 42 indigenous tribes of Kenya and in addition, a sizeable population of Asians, many non-Kenyan Africans, Europeans, and Americans.

According to 2009 National Population and Housing Census, Kenya has a total population of 38,610,097 of which 20,741,420 are children. The Nairobi Cancer Registry covers a population of 3,138,369 million out of which 1,221,067 are children.

Nairobi County hosts the main public teaching and referral hospital in Kenya – Kenyatta National Hospital (KNH). KNH has a capacity of more than 1800 beds. Other than KNH, there are other major private hospitals that offer paediatric oncology services such as The Nairobi Hospital, Aga-Khan University Hospital, Mater Hospital, MP Shah Hospital and Gertrude’s Children’s Hospital. These major referral hospitals have pathology laboratories and cancer specialty clinics for paediatric oncology.

Data are collected from public and private hospitals, Registration of Births and Deaths, Nairobi Hospice and private laboratories within Nairobi County. Cancer registrars visit various sources of data whereby they abstract cases into a case registration form, completed cases are then submitted to NCR office after which a senior cancer registrar and the registry supervisor go through the forms for coding and quality checks based on standards provided by IARC.

#### Years presented

Data for the six-year period 2007–2012 are presented in the following table:

**Table 6.1.4.** Kenya, Nairobi (2007–2012).

	NUMBER OF CASES				REL. FREQ.(%)		RATES PER MILLION					
	0-4	5-9	10-14	All	*M/F*	Overall	0-4	5-9	10-14	Crude	Cum. 0-14	%MV
I Leukaemia	39	43	38	**120**	*1.4*	20.8	16.4	23.4	25.6	21.0	**326.8**	97.5
Ia Lymphoid	32	30	22	**84**	*1.3*	14.6	13.4	16.3	14.8	14.7	**222.8**	100.0
Ib Acute myeloid	4	3	7	**14**	*1.0*	2.4	1.7	1.6	4.7	2.5	**40.2**	100.0
II Lymphoma	15	43	43	**101**	*2.2*	17.5	6.3	23.4	29.0	17.7	**293.3**	96.0
IIa Hodgkin	3	23	14	**40**	*2.6*	6.9	1.3	12.5	9.4	7.0	**116.0**	100.0
IIb NHL (except Burkitt)	2	11	12	**25**	*1.5*	4.3	0.8	6.0	8.1	4.4	**74.6**	96.0
IIc Burkitt	4	6	9	**19**	*2.8*	3.3	1.7	3.3	6.1	3.3	**55.1**	100.0
III Brain and spinal neoplasms	10	16	14	**40**	*0.6*	6.9	4.2	8.7	9.4	7.0	**111.7**	67.5
IVa Neuroblastoma	6	2	2	**10**	*0.7*	1.7	2.5	1.1	1.3	1.8	**24.8**	100.0
V Retinoblastoma	46	3	0	**49**	*1.5*	8.5	19.3	1.6	-	8.6	**104.7**	93.9
VIa Nephroblastoma	40	8	1	**49**	*0.4*	8.5	16.8	4.3	0.7	8.6	**109.0**	81.6
VII Hepatic tumours	1	0	3	**4**	*-*	0.7	0.4	-	2.0	0.7	**12.2**	75.0
VIIa Hepatoblastoma	1	0	0	**1**	*-*	0.2	0.4	-	-	0.2	**2.1**	-
VIIb Hepatic carcinomas	0	0	3	**3**	*-*	0.5	-	-	2.0	0.5	**10.1**	100.0
VIII Bone tumours	3	6	21	**30**	*1.5*	5.2	1.3	3.3	14.2	5.3	**93.4**	80.0
VIIIa Osteosarcoma	1	3	14	**18**	*1.2*	3.1	0.4	1.6	9.4	3.2	**57.5**	83.3
VIIIc Ewing	1	0	2	**3**	*2.0*	0.5	0.4	-	1.3	0.5	**8.8**	100.0
IX Soft tissue sarcomas	27	24	29	**80**	*2.0*	13.9	11.3	13.0	19.6	14.0	**219.7**	95.0
IXa Rhabdomyosarcoma	22	14	14	**50**	*1.3*	8.7	9.2	7.6	9.4	8.8	**131.4**	96.0
IXc Kaposi sarcoma	1	5	7	**13**	*5.5*	2.3	0.4	2.7	4.7	2.3	**39.3**	92.3
X Germ cell tumours	2	2	1	**5**	*0.2*	0.9	0.8	1.1	0.7	0.9	**13.0**	100.0
XI Carcinomas	5	12	36	**53**	*3.1*	9.2	2.1	6.5	24.3	9.3	**164.6**	100.0
Other and unspecified	8	15	13	**36**	*3.0*	6.2	3.4	8.1	8.8	6.3	**101.4**	13.9
TOTAL	202	174	201	**577**	*1.5*	100.0	84.8	94.5	135.6	101.1	**1574.6**	87.2

Population (average annual)		
	**Males**	**Females**
0–4	199,381	197,780
5–9	151,900	154,977
10–14	119,951	127,014
all (0–14)	471,232	479,771

**Source:** National 2009 Census.

#### 6.1.5 Malawi National Cancer Registry

**Figure:**
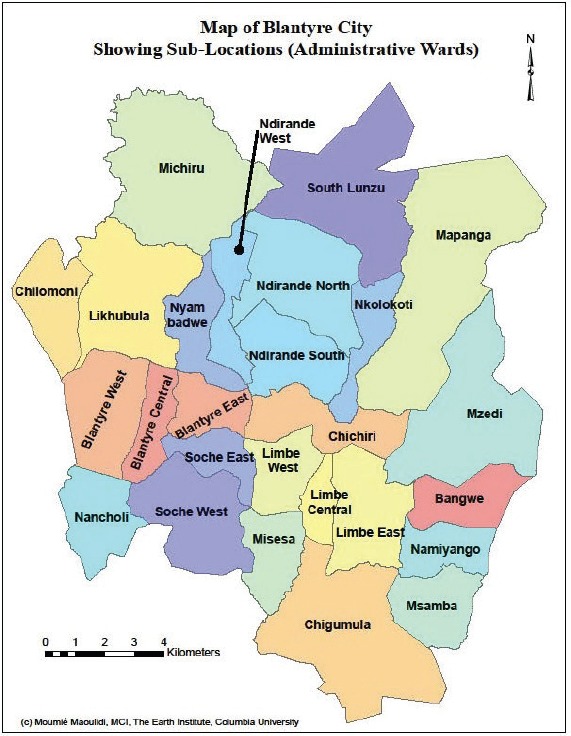
Map of the catchment area served by the cancer registry. Source unknown.

The registry, established in 1989, was initially a histopathology-based registry (recording data on cancer cases diagnosed in the pathology laboratory in Queen Elizabeth’s Hospital (QEH), Blantyre), but in 1993, it became population based, recording cases from all hospitals and clinics serving the population of Blantyre District (Urban and Rural), however diagnosed.

The population of Blantyre District (Blantyre city and some rural areas within it) was slightly more than one million in 2008. The population is widely mixed with various ethnic groups from the whole country, with probably no specific tribe dominating. There is also a significant population of immigrants from neighbouring countries (Tanzania, Zambia, and Mozambique) and small numbers of Zimbabweans, Asians (especially from India), and Europeans.

Registration is carried out by a programme of regular visits by cancer registrars and data clerks to all hospitals (governmental, Christian and private) in the District, with data recorded from hospital records departments and clinical services (including histopathology). The Queen Elizabeth Central Hospital (QECH), a Government Central Hospital, is by far the most important source of data for the registry, as most of the diagnoses for paediatric cancers are made at tertiary-level health care. QECH also has a dedicated cancer ward for children, with the children coming from both within and outside Blantyre. Research links between QECH and health institutions from outside the country also help to provide better diagnostic capabilities for certain specific childhood cancer types. An example of such a linkage is the longstanding QECH-University of Stellenbosch (South Africa) Burkitt’s lymphoma study. Of late new study links have been established at QECH focussing on head and neck tumours in all ages, including children. This development is expected to further improve MCR case finding efforts at QECH.

There is no comprehensive system of death registrations in Malawi, and thus death certificates are not routinely used as a source of information.

The CanReg-4 system was used for data management during the period for which data are presented.

#### Years presented

Data for an eight-year period 2003–2010 are presented in the table below:

**Table 6.1.5.** Malawi, Blantyre (2003–2010).

	NUMBER OF CASES				REL. FREQ.(%)		RATES PER MILLION					
	0-4	5-9	10-14	All	*M/F*	Overall	0-4	5-9	10-14	Crude	Cum. 0-14	%MV
I Leukaemia	6	4	10	**20**	*1.9*	2.0	4.9	3.8	10.9	6.3	**98.0**	60.0
Ia Lymphoid	0	0	1	**1**	*-*	0.1	-	-	1.1	0.3	**5.5**	100.0
Ib Acute myeloid	3	0	0	**3**	*-*	0.3	2.4	-	-	0.9	**12.2**	66.7
II Lymphoma	102	270	134	**506**	*1.6*	51.3	82.6	258.9	146.2	158.4	**2438.5**	70.4
IIa Hodgkin	2	6	10	**18**	*3.5*	1.8	1.6	5.8	10.9	5.6	**91.4**	94.4
IIb NHL (except Burkitt)	10	11	17	**38**	*1.7*	3.9	8.1	10.5	18.5	11.9	**186.0**	78.9
IIc Burkitt	81	235	94	**410**	*1.6*	41.6	65.6	225.3	102.5	128.4	**1967.4**	70.5
III Brain and spinal neoplasms	1	2	0	**3**	*-*	0.3	0.8	1.9	-	0.9	**13.6**	66.7
IVa Neuroblastoma	5	4	0	**9**	*1.2*	0.9	4.1	3.8	-	2.8	**39.4**	55.6
V Retinoblastoma	74	13	0	**87**	*1.0*	8.8	60.0	12.5	-	27.2	**362.1**	65.5
VIa Nephroblastoma	54	23	3	**80**	*1.5*	8.1	43.8	22.1	3.3	25.0	**345.4**	85.0
VII Hepatic tumours	5	9	7	**21**	*2.0*	2.1	4.1	8.6	7.6	6.6	**101.6**	90.5
VIIa Hepatoblastoma	4	4	0	**8**	*3.0*	0.8	3.2	3.8	-	2.5	**35.4**	87.5
VIIb Hepatic carcinomas	1	5	7	**13**	*1.6*	1.3	0.8	4.8	7.6	4.1	**66.2**	92.3
VIII Bone tumours	2	7	15	**24**	*1.0*	2.4	1.6	6.7	16.4	7.5	**123.5**	79.2
VIIIa Osteosarcoma	0	4	7	**11**	*0.8*	1.1	-	3.8	7.6	3.4	**57.4**	72.7
VIIIc Ewing	0	1	3	**4**	*3.0*	0.4	-	1.0	3.3	1.3	**21.2**	100.0
IX Soft tissue sarcomas	50	57	55	**162**	*1.8*	16.4	40.5	54.6	60.0	50.7	**775.8**	62.3
IXa Rhabdomyosarcoma	21	19	12	**52**	*1.6*	5.3	17.0	18.2	13.1	16.3	**241.6**	92.3
IXc Kaposi sarcoma	20	28	35	**83**	*2.1*	8.4	16.2	26.8	38.2	26.0	**406.2**	32.5
X Germ cell tumours	13	4	7	**24**	*0.3*	2.4	10.5	3.8	7.6	7.5	**110.0**	91.7
XI Carcinomas	7	10	9	**26**	*0.9*	2.6	5.7	9.6	9.8	8.1	**125.4**	92.3
Other and unspecified	8	7	9	**24**	*0.7*	2.4	6.5	6.7	9.8	7.5	**115.1**	45.8
TOTAL	327	410	249	**986**	*1.4*	100.0	264.9	393.1	271.6	308.7	**4648.4**	70.6

Population (average annual)		
	**Males**	**Females**
0-4	76,472	77,804
5–9	63,844	66,534
10–14	54,662	59,920
all (0–14)	194,977	204,257

**Source:** Population estimates for each individual year for the period 2001−2010, based on:

• National Statistical Office of Malawi. 1998 Population and Housing Census. Analytic report

• National Statistical Office of Malawi. 2008 Population and Housing Census.

• Detailed Tables for Blantyre Urban (A3.30) and Rural (A3.31)

• Assuming constant rates of increase between censuses in each sex-age group

• Estimates for 2009 & 2010 assume the same rates of growth (in each sex-age group) are continued

#### 6.1.6 Mauritius National Cancer Registry

**Figure:**
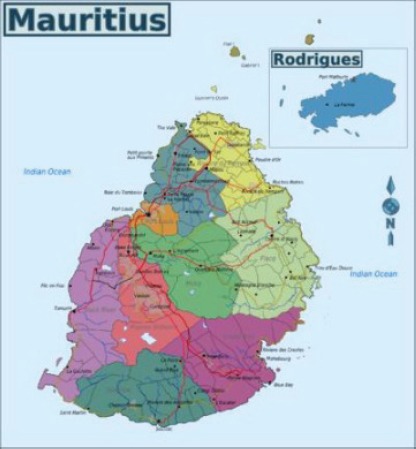
Map of the catchment area served by the cancer registry. Source unknown.

The Mauritius National Cancer Registry (MNCR) covers the entire Republic of Mauritius including mainly the islands of Mauritius and Rodrigues; an area of 1968.8 sq km. The total population is of at 1.29 million on 31 December 2012. It is a multi-racial population consisting of 68% Indo–Mauritians, 27% creoles, 3% Sino–Mauritians, and 2% Franco–Mauritians. Since 1989, the Mauritius National Cancer Registry is operational and has reached a population-based level (PBCR) in 2001. The registry is supported by the Ministry of Health and Quality of Life of Mauritius and is based in the Central Health Laboratory, Victoria Hospital, Quatre Bornes.

Data have been collected on an annual basis retrospectively and semi-actively from various sources, namely radiotherapy register, pathology laboratory archives, overseas treatment unit, private pathologists, and private clinics. All private pathologists in Mauritius have been convinced to supply data on cases of cancer they diagnose, according to a simplified standard format. Data have been obtained from that particular source as from 2000. Unfortunately, the data are not complete for a few of them. A link with the civil registration office gives access to cancer-specific mortality data from death certificates. However, these data are not entered as primary data but used to update status on cases that mention cancer as the cause of death.

There is a free healthcare delivery system across the Republic of Mauritius. As regards to diagnosis of leukaemias, bone marrow aspiration, and biopsy facilities exist at regional hospital level. Positive samples are sent abroad for immunophenotyping and establishment of treatment protocols; following which the patients are admitted in a well-equipped, dedicated children’s ward with 12 bed capacity and a wide range of chemotherapeutic drugs. Concerning solid tumours, a nationwide paediatric surgery service is available with two dedicated paediatric surgeons.

The CANREG is used for data entry and management.

#### Years presented

Data for a 10-year period 2003–2012 are presented in the following table:

**Table 6.1.6.** Mauritius (2003–2012).

	NUMBER OF CASES				REL. FREQ.(%)		RATES PER MILLION					
	0-4	5-9	10-14	All	*M/F*	Overall	0-4	5-9	10-14	Crude	Cum. 0-14	%MV
I Leukaemia	48	29	20	**97**	*1.9*	35.9	54.1	29.9	19.4	33.6	**517.4**	99.0
Ia Lymphoid	15	11	6	**32**	*1.5*	11.9	16.9	11.3	5.8	11.1	**170.5**	100.0
Ib Acute myeloid	5	3	3	**11**	*2.7*	4.1	5.6	3.1	2.9	3.8	**58.2**	100.0
II Lymphoma	4	13	11	**28**	*3.0*	10.4	4.5	13.4	10.7	9.7	**143.0**	100.0
IIa Hodgkin	1	6	6	**13**	*3.3*	4.8	1.1	6.2	5.8	4.5	**65.7**	100.0
IIb NHL (except Burkitt)	3	2	2	**7**	*2.5*	2.6	3.4	2.1	1.9	2.4	**36.9**	100.0
IIc Burkitt	0	1	1	**2**	*1.0*	0.7	-	1.0	1.0	0.7	**10.0**	100.0
III Brain and spinal neoplasms	11	10	5	**26**	*0.9*	9.6	12.4	10.3	4.9	9.0	**137.9**	100.0
IVa Neuroblastoma	7	3	0	**10**	*2.3*	3.7	7.9	3.1	-	3.5	**54.9**	100.0
V Retinoblastoma	7	1	0	**8**	*0.3*	3.0	7.9	1.0	-	2.8	**44.6**	100.0
VIa Nephroblastoma	9	1	0	**10**	*0.7*	3.7	10.2	1.0	-	3.5	**55.9**	100.0
VII Hepatic tumours	2	0	0	**2**	*1.0*	0.7	2.3	-	-	0.7	**11.3**	100.0
VIIa Hepatoblastoma	1	0	0	**1**	*-*	0.4	1.1	-	-	0.3	**5.6**	100.0
VIIb Hepatic carcinomas	1	0	0	**1**	*-*	0.4	1.1	-	-	0.3	**5.6**	100.0
VIII Bone tumours	1	6	7	**14**	*3.7*	5.2	1.1	6.2	6.8	4.9	**70.6**	92.9
VIIIa Osteosarcoma	0	3	5	**8**	*7.0*	3.0	-	3.1	4.9	2.8	**39.8**	100.0
VIIIc Ewing	0	2	1	**3**	*2.0*	1.1	-	2.1	1.0	1.0	**15.2**	100.0
IX Soft tissue sarcomas	7	5	12	**24**	*1.0*	8.9	7.9	5.2	11.7	8.3	**123.6**	100.0
IXa Rhabdomyosarcoma	4	1	2	**7**	*1.3*	2.6	4.5	1.0	1.9	2.4	**37.4**	100.0
IXc Kaposi sarcoma	0	0	0	**0**	*-*	-	-	-	-	-	**-**	-
X Germ cell tumours	6	1	7	**14**	*1.3*	5.2	6.8	1.0	6.8	4.9	**73.0**	100.0
XI Carcinomas	1	2	8	**11**	*1.2*	4.1	1.1	2.1	7.8	3.8	**54.8**	100.0
Other and unspecified	8	6	12	**26**	*1.6*	9.6	9.0	6.2	11.7	9.0	**134.4**	53.8
TOTAL	111	77	82	**270**	* 1.5*	100.0	125.2	79.4	79.7	93.6	**1421.4**	94.8

Population (average annual)		
	**Males**	**Females**
0–4	45,044	43,624
5–9	49,250	47,716
10–14	52,091	50,802
all (0–14)	146,385	142,142

**Source:** The Central Statistics Office Mauritius provides estimates of yearly population for the Republic of Mauritius. These figures are available on the annual health statistics report released by the Ministry of Health and Quality of Life. The estimate of the population at-risk is based on the 2000 census making allowance for births, deaths and migration into and out of the Republic of Mauritius.

#### 6.1.7 Kampala Cancer Registry

**Figure:**
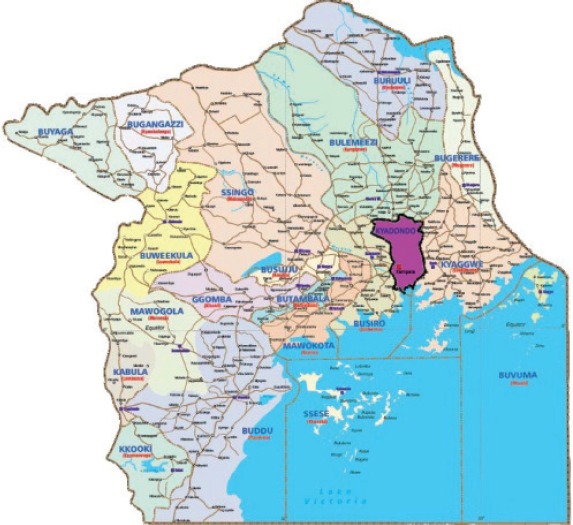
Map of the catchment area served by the cancer registry. Source unknown.

The registry was established in the Department of Pathology of Makerere Faculty of Medicine (now the College of Health Sciences, Makerere University) in 1951 as a population-based cancer registry with the aim of determining cancer incidence in the population of Kyadondo County, which comprises Kampala District (including the city of Kampala – the capital of Uganda) and part of Wakiso District. The population was estimated at 2,010,000 in 2009, and the inhabitants come from all of the 31 ethnic groups found in Uganda, although the majority (about 50%) is from the Ganda ethnicity. There are also many migrants from neighbouring countries, particularly from Kenya, Sudan, and Rwanda. One percent of the population is of European and Asian origin.

Registration is both active and passive, with the assistance of some doctors and medical records staff, in Mulago referral hospital and complex (including radiotherapy and haematology departments), the Uganda Cancer Institute, and three moderately sized private hospitals, as well as private clinics and nursing homes. Information is also collected from Hospice Uganda, as well as on cancer cases diagnosed in the main University Histopathology laboratory, as well as several private laboratories.

There is no system for civil registration of deaths, by cause, in Uganda; however, death certificates are issued for all deaths occurring in hospital and copied into a death register in the hospital mortuary. This source of information is used by the registry.

There are specialised treatment services for paediatric oncology available in the Uganda Cancer Institute.

Data management is carried out using CANREG software.

#### Years presented

Data for a 10-year period 2003–2012 are presented in the following table.

**Table 6.1.7.** Uganda, Kyadondo County (2003–2012).

	NUMBER OF CASES				REL. FREQ.(%)		RATES PER MILLION					
	0-4	5-9	10-14	All	*M/F*	Overall	0-4	5-9	10-14	Crude	Cum. 0-14	%MV
I Leukaemia	24	39	38	**101**	*2.2*	8.1	8.0	14.7	14.4	12.2	**185.5**	49.5
Ia Lymphoid	13	20	15	**48**	*2.0*	3.8	4.4	7.5	5.7	5.8	**87.8**	58.3
Ib Acute myeloid	1	8	3	**12**	*3.0*	1.0	0.3	3.0	1.1	1.4	**22.4**	58.3
II Lymphoma	99	224	123	**446**	*1.4*	35.6	33.1	84.2	46.6	53.8	**819.9**	69.1
IIa Hodgkin	10	15	21	**46**	*2.3*	3.7	3.3	5.6	8.0	5.6	**84.7**	87.0
IIb NHL (except Burkitt)	19	36	24	**79**	*1.2*	6.3	6.4	13.5	9.1	9.5	**145.0**	89.9
IIc Burkitt	56	146	48	**250**	*1.2*	19.9	18.7	54.9	18.2	30.2	**459.1**	66.4
III Brain and spinal neoplasms	14	13	12	**39**	*0.8*	3.1	4.7	4.9	4.5	4.7	**70.6**	23.1
IVa Neuroblastoma	6	1	1	**8**	*1.0*	0.6	2.0	0.4	0.4	1.0	**13.8**	62.5
V Retinoblastoma	53	7	2	**62**	*1.1*	4.9	17.7	2.6	0.8	7.5	**105.7**	71.0
VIa Nephroblastoma	70	19	6	**95**	*1.3*	7.6	23.4	7.1	2.3	11.5	**164.3**	84.2
VII Hepatic tumours	4	2	5	**11**	*1.2*	0.9	1.3	0.8	1.9	1.3	**19.9**	72.7
VIIa Hepatoblastoma	1	2	0	**3**	*0.5*	0.2	0.3	0.8	-	0.4	**5.4**	100.0
VIIb Hepatic carcinomas	3	0	4	**7**	*1.3*	0.6	1.0	-	1.5	0.8	**12.6**	71.4
VIII Bone tumours	3	10	30	**43**	*1.0*	3.4	1.0	3.8	11.4	5.2	**80.7**	53.5
VIIIa Osteosarcoma	0	4	15	**19**	*0.7*	1.5	-	1.5	5.7	2.3	**35.9**	84.2
VIIIc Ewing	0	0	1	**1**	*-*	0.1	-	-	0.4	0.1	**1.9**	100.0
IX Soft tissue sarcomas	76	115	120	**311**	*1.5*	24.8	25.4	43.2	45.5	37.5	**570.8**	74.9
IXa Rhabdomyosarcoma	16	15	13	**44**	*1.4*	3.5	5.4	5.6	4.9	5.3	**79.6**	81.8
IXc Kaposi sarcoma	57	89	94	**240**	*1.5*	19.1	19.1	33.5	35.6	29.0	**440.9**	75.4
X Germ cell tumours	2	1	9	**12**	*0.5*	1.0	0.7	0.4	3.4	1.4	**22.3**	50.0
XI Carcinomas	6	8	17	**31**	*0.8*	2.5	2.0	3.0	6.4	3.7	**57.3**	90.3
Other and unspecified	42	27	26	**95**	*1.2*	7.6	14.1	10.1	9.9	11.5	**170.3**	2.1
TOTAL	399	466	389	**1254**	* 1.4*	100.0	133.6	175.2	147.5	151.4	**2281.1**	63.5

Population (average annual)		
	**Males**	**Females**
0–4	147,804	150,878
5–9	128,883	137,140
10–14	119,699	144,109
all (0–14)	396,385	432,126

**Source:** 2002 Census results from Uganda Statistical service 6 December 2007.

2014 Preliminary results from census

Population data for 2003−2012 assume constant rate of growth within age groups between census counts (2002 and 2014).

#### 6.1.8 Zimbabwe National Cancer Registry: Harare (Africans)

**Figure:**
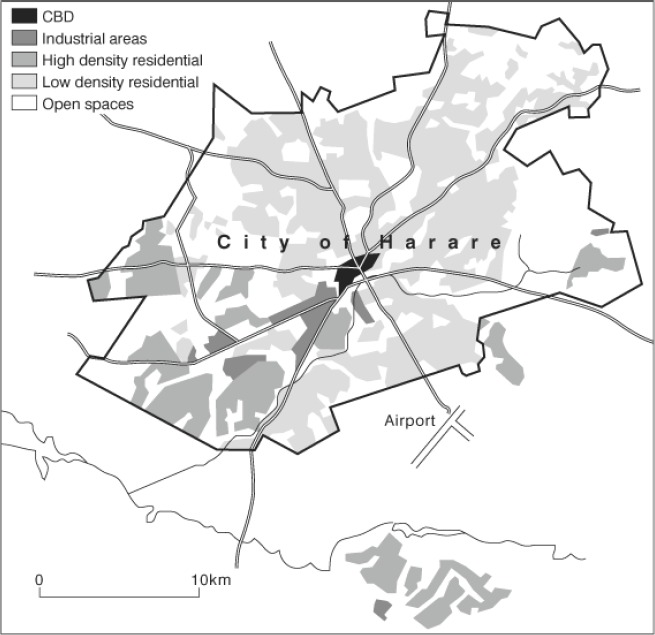
Map of the catchment area served by the cancer registry. Source unknown.

The Zimbabwe National Cancer Registry (ZNCR) was established in 1985 as a result of a collaborative research agreement between the Ministry of Health and Child Care (MHCC) and the International Agency for Research on Cancer (IARC). It is situated in the Parirenyatwa Group of Hospitals complex, a large government tertiary referral centre, which provides most of the cancer management services for the northern part of the country and is one of the two teaching hospitals of the University of Zimbabwe’s College of Health Sciences.

ZNCR aims to record all cancer cases occurring in the population of the capital city of Harare. It also records information on cancer cases from other parts of the country. The population of Harare city at the census of 2012 was 1.49 million, of which, 98% were of African (black) origin.

The ZNCR uses a combination of active and passive methods of case finding. Regular routine visits are made to the inpatient wards, oncology outpatient clinics and medical records departments of the three government referral hospitals (Parirenyatwa Group of Hospitals, Harare Central Hospital, and Chitungwiza Central Hospital), three major private hospitals and the two municipal infectious diseases hospitals in Harare. Other important sources of information are public and private pathology laboratories, the radiology and haematology departments at Parirenyatwa Group of Hospitals and the Harare Death Registry.

The paediatric oncology unit (POU) at Parirenyatwa Hospital is the main referral centre for children with malignancies. There is reasonable access to chemotherapy drugs with major chemotherapy drugs being available regularly and consistently to over 80% of children with cancer. The drugs are a donation by a charitable non – governmental organisation. Children also have access to imaging, laboratory services including flow cytometry for leukaemias. However, there are no facilities for bone marrow transplant. Surgery for children with cancer is performed by a dedicated paediatric surgical unit at Harare Central Hospital. After operations, the patients are sent back to the POU for the rest of the management. Children who require radiotherapy are referred to the radio-oncology unit, which although caters mainly for adults has facilities also for children.

Death certificates of people who die in the greater Harare and the dormitory town of Chitungwiza are scrutinised weekly to record those that have died of malignant disease. Deaths apparently from cancer, which cannot be found in clinical records, are registered as ‘Death Certificate Only’ (DCO). Some 6% of registrations of cancer in childhood in 2003–2012 were DCO cases.

The CANREG system is used for data entry and management.

#### Years presented

Data for a 10-year period 2003–2012 are presented in the following table.

**Table 6.1.8.** Zimbabwe, Harare: African (2003–2012).

	NUMBER OF CASES				REL. FREQ.(%)		RATES PER MILLION					
	0-4	5-9	10-14	All	*M/F*	Overall	0-4	5-9	10-14	Crude	Cum. 0-14	%MV
I Leukaemia	24	18	20	**62**	*1.5*	12.3	12.4	12.6	15.2	13.2	**200.8**	93.5
Ia Lymphoid	9	11	11	**31**	*1.2*	6.2	4.7	7.7	8.3	6.6	**103.4**	90.3
Ib Acute myeloid	6	2	2	**10**	*1.0*	2.0	3.1	1.4	1.5	2.1	**30.1**	100.0
II Lymphoma	13	27	37	**77**	*1.8*	15.3	6.7	18.9	28.0	16.4	**268.1**	96.1
IIa Hodgkin	0	8	7	**15**	*2.8*	3.0	-	5.6	5.3	3.2	**54.5**	100.0
IIb NHL (except Burkitt)	8	12	26	**46**	*1.9*	9.1	4.1	8.4	19.7	9.8	**161.1**	95.7
IIc Burkitt	3	6	2	**11**	*1.8*	2.2	1.6	4.2	1.5	2.3	**36.3**	100.0
III Brain and spinal neoplasms	16	19	10	**45**	*1.0*	8.9	8.3	13.3	7.6	9.6	**145.7**	44.4
IVa Neuroblastoma	5	1	1	**7**	*2.5*	1.4	2.6	0.7	0.8	1.5	**20.2**	100.0
V Retinoblastoma	35	4	0	**39**	*1.6*	7.7	18.1	2.8	-	8.3	**104.6**	94.9
VIa Nephroblastoma	50	16	7	**73**	*0.7*	14.5	25.9	11.2	5.3	15.6	**211.9**	100.0
VII Hepatic tumours	2	1	4	**7**	*2.5*	1.4	1.0	0.7	3.0	1.5	**23.8**	28.6
VIIa Hepatoblastoma	2	0	0	**2**	*-*	0.4	1.0	-	-	0.4	**5.2**	50.0
VIIb Hepatic carcinomas	0	0	3	**3**	*2.0*	0.6	-	-	2.3	0.6	**11.4**	33.3
VIII Bone tumours	5	5	17	**27**	*1.2*	5.4	2.6	3.5	12.9	5.8	**94.8**	77.8
VIIIa Osteosarcoma	3	2	9	**14**	*1.0*	2.8	1.6	1.4	6.8	3.0	**48.9**	92.9
VIIIc Ewing	1	1	1	**3**	*2.0*	0.6	0.5	0.7	0.8	0.6	**9.9**	100.0
IX Soft tissue sarcomas	27	57	42	**126**	*1.3*	25.0	14.0	39.8	31.8	26.9	**428.1**	69.8
IXa Rhabdomyosarcoma	8	8	4	**20**	*1.9*	4.0	4.1	5.6	3.0	4.3	**63.8**	100.0
IXc Kaposi sarcoma	13	40	29	**82**	*1.3*	16.3	6.7	27.9	22.0	17.5	**283.2**	54.9
X Germ cell tumours	2	1	2	**5**	*0.2*	1.0	1.0	0.7	1.5	1.1	**16.3**	40.0
XI Carcinomas	4	6	11	**21**	*0.5*	4.2	2.1	4.2	8.3	4.5	**73.0**	100.0
Other and unspecified	7	6	2	**15**	*1.1*	3.0	3.6	4.2	1.5	3.2	**46.7**	20.0
TOTAL	190	161	153	**504**	* 1.2*	100.0	98.4	112.5	115.9	107.7	**1634.1**	80.6

Population (average annual)		
	**Males**	**Females**
0–4	96,060	96,954
5–9	70,082	73,072
10–14	62,692	69,297
all (0–14)	228,833	239,323

**Source:** 2002 and 2012 Census results.

Estimates 2003−2012 assume constant rate of growth within age groups between census counts (2002 and 2012).

### 6.2. Southern Africa

#### 6.2.1 Botswana National Cancer Registry

**Figure:**
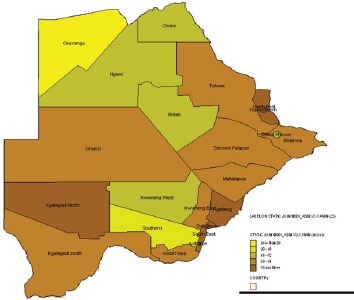
Map of the catchment area served by the cancer registry. Source unknown.

The Botswana National Cancer Registry was first founded in 1999 as a population-based cancer registry. It was resuscitated in 2003/2004 following a period of inactivity, with the assistance of the International Agency for Research on Cancer (IARC). The registry collects data on patient demographics, risk factors, management of cancers, and other related information, from patients diagnosed and managed in Botswana.

The registry is located in the Department of Public Health and funded by the MoH. The registry is staffed by a registry manager and two registrars. Cancer registration is one of the activities of the NCD Programme.

The Population and Housing Census of 2011 showed that Botswana has a population of 2,024,787 million. The Tswana are the majority ethnic group in Botswana, making up 79% of the population. An estimated 70% of the country’s citizens identify themselves as Christians, with Anglicans, Methodists, and the United Congregational Church of Southern Africa making up the majority.

Cancer cases are collected from four public facilities and one private facility. Public facilities offer basic diagnostic and treatment services, while radiation therapy is offered at the Gaborone Private Hospital which is accessible to public patients through government subsidy. Health care in Botswana is free including oncology care.

Botswana has effectively collaborated with Baylor Centre of Excellence to improve the clinical care for children with cancer and haematological conditions through visiting resident oncologists and haematologists.

Data collection is essentially active. The registrars periodically visit the identified data sources for case finding and abstraction. The Botswana National Cancer Registry collects information from the two referral hospitals (Princess Marina Hospital, Nyangabgwe Referral Hospital) and the radiotherapy department of the Gaborone Private Hospital and also from the two district oncology centres at the Letsholathebe and Sekgoma Memorial Hospitals. The registry also collects information from pathology labs and the Integrated Patient Management System (IPMS).

Information on deaths from cancer in hospital is obtained from the Health Statistics Unit in the Ministry of Health (Department of Policy, Planning, Monitoring and Evaluation (DPPME)).

The CanReg4 software is used for data entry and management.

#### Years presented

Data for four-year period 2005–2008 are presented in the table below.

**Table 6.2.1.** Botswana (2003–2008).

	NUMBER OF CASES				REL. FREQ.(%)		RATES PER MILLION					
	0-4	5-9	10-14	All	*M/F*	Overall	0-4	5-9	10-14	Crude	Cum. 0-14	%MV
I Leukaemia	12	18	14	**44**	*1.3*	16.5	9.4	14.2	11.2	11.6	**173.7**	100.0
Ia Lymphoid	10	12	3	**25**	*2.1*	9.4	7.8	9.5	2.4	6.6	**98.3**	100.0
Ib Acute myeloid	2	6	7	**15**	*0.9*	5.6	1.6	4.7	5.6	3.9	**59.4**	100.0
II Lymphoma	9	18	14	**41**	*2.4*	15.4	7.0	14.2	11.2	10.8	**162.0**	100.0
IIa Hodgkin	2	3	7	**12**	*2.0*	4.5	1.6	2.4	5.6	3.2	**47.6**	100.0
IIb NHL (except Burkitt)	3	13	4	**20**	*1.9*	7.5	2.3	10.3	3.2	5.3	**78.9**	100.0
IIc Burkitt	2	0	0	**2**	*-*	0.7	1.6	-	-	0.5	**7.8**	100.0
III Brain and spinal neoplasms	3	7	12	**22**	*2.1*	8.2	2.3	5.5	9.6	5.8	**87.2**	90.9
IVa Neuroblastoma	9	0	0	**9**	*0.3*	3.4	7.0	-	-	2.4	**35.1**	100.0
V Retinoblastoma	19	1	0	**20**	*1.5*	7.5	14.8	0.8	-	5.3	**78.1**	100.0
VIa Nephroblastoma	12	7	3	**22**	*0.8*	8.2	9.4	5.5	2.4	5.8	**86.4**	100.0
VII Hepatic tumours	3	0	2	**5**	*4.0*	1.9	2.3	-	1.6	1.3	**19.7**	60.0
VIIa Hepatoblastoma	2	0	0	**2**	*-*	0.7	1.6	-	-	0.5	**7.8**	100.0
VIIb Hepatic carcinomas	0	0	2	**2**	*1.0*	0.7	-	-	1.6	0.5	**8.0**	50.0
VIII Bone tumours	1	1	15	**17**	*0.5*	6.4	0.8	0.8	12.0	4.5	**67.7**	100.0
VIIIa Osteosarcoma	0	1	9	**10**	*1.0*	3.7	-	0.8	7.2	2.6	**39.9**	100.0
VIIIc Ewing	0	0	0	**0**	*-*	-	-	-	-	-	**-**	-
IX Soft tissue sarcomas	13	27	17	**57**	*1.0*	21.3	10.1	21.3	13.6	15.0	**225.0**	68.4
IXa Rhabdomyosarcoma	3	4	2	**9**	*3.5*	3.4	2.3	3.2	1.6	2.4	**35.5**	100.0
IXc Kaposi sarcoma	6	21	13	**40**	*0.8*	15.0	4.7	16.6	10.4	10.5	**158.1**	55.0
X Germ cell tumours	3	1	2	**6**	*0.5*	2.2	2.3	0.8	1.6	1.6	**23.6**	83.3
XI Carcinomas	6	9	7	**22**	*1.8*	8.2	4.7	7.1	5.6	5.8	**86.8**	100.0
Other and unspecified	1	0	1	**2**	*1.0*	0.7	0.8	-	0.8	0.5	**7.9**	-
TOTAL	91	89	87	**267**	* 1.2*	100.0	71.0	70.2	69.4	70.2	**1053.2**	90.6

Population (average annual)		
	**Males**	**Females**
0–4	107,812	105,738
5–9	106,103	105,265
10–14	104,560	104,266
all (0–14)	318,474	315,269

**Source:** Population estimates for each individual year for the period, based on 2001 and 2011 census results.

Method: annual percentage change by sex and age groups.

#### 6.2.2 RSA: South African Children’s Cancer Study Group (SACCSG)

**Figure:**
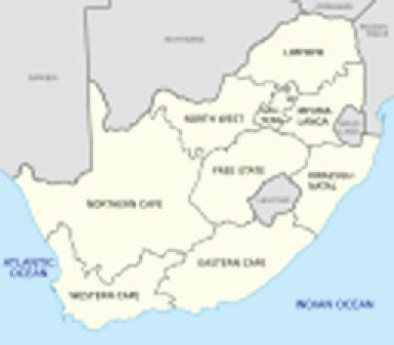
Map of the catchment area served by the cancer registry. Source unknown.

The South African Paediatric Registry commenced in 1987 as an initiative of the South African Children’s Cancer Study Group (SACCSG). Paediatric oncology units in the country collaborate, and data also come from several oncologists and interested parties in private practice. All units have ethical approval to collect and submit data to the Registry. The Registry was hosted over time by various paediatric oncology units in the country; for the last 15 years, it has been kept at Stellenbosch University.

The Registry is essential for collecting data on malignant disease in the paediatric population. It is a central repository of data provided by individual, hospital-based registries throughout the country. These registries record data on all patients presenting to paediatric oncology services; however, children who die without being seen at a hospital, and those treated by private oncologists and other paediatric subspecialists (e.g. neurosurgeons), might not be recorded. Although a central registry of hospital-based registers cannot be complete, it nevertheless has data that can be used for statistical research in an efficient and sustainable way.

The data collected in the hospital-based registry are: demographic information (name, age, sex, and address); tumour type, localisation, and stage; histological type; treatment and outcome; and yearly follow-up results. Data should include the date and cause of death, if the patient died.

The Republic of South Africa is situated at the southernmost part of the continent, covering a surface comparable with that of the Central Europe. Its total population was estimated, for mid-2011, at 50.6 million inhabitants. Out of these, 31.3% (corresponding to 15.8 million) are children under 15 years of age. The SA childhood cancer registry covers the entire country.

In South Africa, the majority of children with cancer are treated in the public healthcare sector, at tertiary (subspecialist) level. A few patients would be managed by private oncologists or surgeons. Clinics, district hospitals and second level hospitals (general specialist hospitals) do not treat oncology cases but refer them to tertiary level once a presumptive or definitive diagnosis is established. There are seven paediatric oncology centres covering the whole country:

- Tygerberg Hospital
- The Red Cross Memorial Children’s Hospital
- Universitas Hospital
- Chris Hani – Baragwanath Hospital
- Charlotte Maxeke Hospital
- Steve Biko Academic Hospital
- Inkosi Albert Luthuli Hospital
- There are a few satellite units in Limpopo (Northern part of the country) and Eastern Cape (East London and Port Elizabeth)

#### National Cancer Registry

Until April 2011, the Registry was the main source of statistical data for epidemiological studies in this field. An alternative source of cancer information was the National Cancer Registry, a pathology-based registry of the National Health Laboratory Service, established in 1986.

#### Years presented

Data for a five-year period 2008–2012 are presented in the following table.

**Table 6.2.2.** South African Republic (2008–2012).

	NUMBER OF CASES				REL. FREQ.(%)		RATES PER MILLION					
	0-4	5-9	10-14	All	*M/F*	Overall	0-4	5-9	10-14	Crude	Cum. 0-14	%MV
I Leukaemia	374	296	210	**880**	*1.3*	25.3	13.4	12.3	9.0	11.7	**173.9**	99.3
Ia Lymphoid	259	207	134	**600**	*1.6*	17.2	9.3	8.6	5.8	8.0	**118.4**	99.2
Ib Acute myeloid	103	79	62	**244**	*1.0*	7.0	3.7	3.3	2.7	3.2	**48.3**	99.6
II Lymphoma	124	209	169	**502**	*2.5*	14.4	4.5	8.7	7.3	6.7	**102.0**	97.2
IIa Hodgkin	21	88	76	**185**	*3.4*	5.3	0.8	3.6	3.3	2.5	**38.4**	98.9
IIb NHL (except Burkitt)	42	79	65	**186**	*1.7*	5.3	1.5	3.3	2.8	2.5	**37.9**	95.7
IIc Burkitt	60	42	27	**129**	*3.2*	3.7	2.2	1.7	1.2	1.7	**25.3**	96.9
III Brain and spinal neoplasms	189	156	94	**439**	*1.1*	12.6	6.8	6.5	4.1	5.8	**86.6**	67.7
IVa Neuroblastoma	164	24	9	**197**	*1.0*	5.7	5.9	1.0	0.4	2.6	**36.4**	96.4
V Retinoblastoma	209	17	4	**230**	*1.1*	6.6	7.5	0.7	0.2	3.1	**42.0**	59.6
VIa Nephroblastoma	358	100	12	**470**	*0.9*	13.5	12.9	4.1	0.5	6.3	**87.7**	96.0
VII Hepatic tumours	43	10	9	**62**	*1.1*	1.8	1.5	0.4	0.4	0.8	**11.7**	77.4
VIIa Hepatoblastoma	41	10	1	**52**	*1.2*	1.5	1.5	0.4	0.0	0.7	**9.7**	76.9
VIIb Hepatic carcinomas	1	0	8	**9**	*1.2*	0.3	0.0	-	0.3	0.1	**1.9**	88.9
VIII Bone tumours	12	41	70	**123**	*1.2*	3.5	0.4	1.7	3.0	1.6	**25.7**	97.6
VIIIa Osteosarcoma	3	26	53	**82**	*1.2*	2.4	0.1	1.1	2.3	1.1	**17.3**	96.3
VIIIc Ewing	8	14	14	**36**	*1.2*	1.0	0.3	0.6	0.6	0.5	**7.4**	100.0
IX Soft tissue sarcomas	168	112	86	**366**	*1.2*	10.5	6.0	4.6	3.7	4.9	**72.0**	91.8
IXa Rhabdomyosarcoma	104	47	31	**182**	*1.2*	5.2	3.7	1.9	1.3	2.4	**35.1**	96.2
IXc Kaposi sarcoma	25	44	34	**103**	*1.1*	3.0	0.9	1.8	1.5	1.4	**20.9**	79.6
X Germ cell tumours	82	22	30	**134**	*0.3*	3.8	2.9	0.9	1.3	1.8	**25.8**	91.0
XI Carcinomas	12	14	37	**63**	*0.9*	1.8	0.4	0.6	1.6	0.8	**13.0**	98.4
Other and unspecified	6	5	6	**17**	*1.4*	0.5	0.2	0.2	0.3	0.2	**3.4**	76.5
TOTAL	1741	1006	736	**3483**	* 1.2*	100.0	62.6	41.7	31.7	46.4	**680.2**	90.1

**Table 6.2.2.** South African Republic: Black (2008–2012).

	NUMBER OF CASES				REL. FREQ.(%)		RATES PER MILLION					
	0-4	5-9	10-14	All	*M/F*	Overall	0-4	5-9	10-14	Crude	Cum. 0-14	%MV
I Leukaemia	222	217	143	**582**	*1.3*	22.5	9.4	10.7	7.4	9.2	**137.6**	99.1
Ia Lymphoid	134	139	86	**359**	*1.4*	13.9	5.7	6.9	4.5	5.7	**84.9**	98.9
Ib Acute myeloid	79	68	47	**194**	*1.1*	7.5	3.3	3.4	2.4	3.1	**45.7**	99.5
II Lymphoma	105	161	127	**393**	*2.4*	15.2	4.4	7.9	6.6	6.2	**94.8**	96.9
IIa Hodgkin	17	67	56	**140**	*3.5*	5.4	0.7	3.3	2.9	2.2	**34.6**	98.6
IIb NHL (except Burkitt)	35	63	53	**151**	*1.5*	5.8	1.5	3.1	2.7	2.4	**36.7**	96.0
IIc Burkitt	52	31	17	**100**	*3.3*	3.9	2.2	1.5	0.9	1.6	**23.1**	96.0
III Brain and spinal neoplasms	118	102	66	**286**	*1.1*	11.1	5.0	5.0	3.4	4.5	**67.2**	64.7
IVa Neuroblastoma	111	19	7	**137**	*1.0*	5.3	4.7	0.9	0.4	2.2	**30.0**	95.6
V Retinoblastoma	179	17	3	**199**	*1.1*	7.7	7.6	0.8	0.2	3.1	**42.9**	58.3
VIa Nephroblastoma	291	85	9	**385**	*0.9*	14.9	12.3	4.2	0.5	6.1	**84.9**	95.6
VII Hepatic tumours	33	6	8	**47**	*1.1*	1.8	1.4	0.3	0.4	0.7	**10.5**	76.6
VIIa Hepatoblastoma	32	6	1	**39**	*1.2*	1.5	1.4	0.3	0.1	0.6	**8.5**	74.4
VIIb Hepatic carcinomas	0	0	7	**7**	*1.3*	0.3	-	-	0.4	0.1	**1.8**	100.0
VIII Bone tumours	9	29	52	**90**	*1.2*	3.5	0.4	1.4	2.7	1.4	**22.5**	96.7
VIIIa Osteosarcoma	1	20	43	**64**	*1.3*	2.5	0.0	1.0	2.2	1.0	**16.3**	95.3
VIIIc Ewing	7	8	7	**22**	*1.2*	0.9	0.3	0.4	0.4	0.3	**5.3**	100.0
IX Soft tissue sarcomas	134	102	70	**306**	*1.2*	11.8	5.7	5.0	3.6	4.8	**71.7**	90.5
IXa Rhabdomyosarcoma	78	40	23	**141**	*1.3*	5.5	3.3	2.0	1.2	2.2	**32.3**	95.7
IXc Kaposi sarcoma	23	44	32	**99**	*1.1*	3.8	1.0	2.2	1.7	1.6	**24.0**	78.8
X Germ cell tumours	60	17	27	**104**	*0.2*	4.0	2.5	0.8	1.4	1.6	**23.9**	89.4
XI Carcinomas	5	9	25	**39**	*1.1*	1.5	0.2	0.4	1.3	0.6	**9.8**	97.4
Other and unspecified	4	5	6	**15**	*1.5*	0.6	0.2	0.2	0.3	0.2	**3.6**	73.3
TOTAL	1271	769	543	**2583**	* 1.2*	100.0	53.8	37.9	28.1	40.9	**599.4**	89.0

**Table 6.2.2.** South African Republic: White (2008–2012).

	NUMBER OF CASES				REL. FREQ.(%)		RATES PER MILLION					
	0-4	5-9	10-14	All	*M/F*	Overall	0-4	5-9	10-14	Crude	Cum. 0-14	%MV
I Leukaemia	57	19	30	**106**	*1.5*	31.2	43.1	15.3	22.8	27.3	**406.1**	100.0
Ia Lymphoid	48	18	23	**89**	*2.0*	26.2	36.3	14.5	17.5	22.9	**341.5**	100.0
Ib Acute myeloid	7	1	4	**12**	*0.2*	3.5	5.3	0.8	3.0	3.1	**45.7**	100.0
II Lymphoma	4	19	21	**44**	*3.0*	12.9	3.0	15.3	16.0	11.3	**171.5**	97.7
IIa Hodgkin	0	12	8	**20**	*4.0*	5.9	-	9.7	6.1	5.2	**78.8**	100.0
IIb NHL (except Burkitt)	4	4	8	**16**	*2.2*	4.7	3.0	3.2	6.1	4.1	**61.6**	93.8
IIc Burkitt	0	3	5	**8**	*3.0*	2.4	-	2.4	3.8	2.1	**31.1**	100.0
III Brain and spinal neoplasms	30	14	8	**52**	*0.8*	15.3	22.7	11.3	6.1	13.4	**200.3**	76.9
IVa Neuroblastoma	18	3	0	**21**	*1.1*	6.2	13.6	2.4	-	5.4	**80.2**	100.0
V Retinoblastoma	12	0	1	**13**	*0.6*	3.8	9.1	-	0.8	3.4	**49.2**	61.5
VIa Nephroblastoma	25	5	0	**30**	*1.7*	8.8	18.9	4.0	-	7.7	**114.7**	96.7
VII Hepatic tumours	6	2	0	**8**	*1.0*	2.4	4.5	1.6	-	2.1	**30.8**	75.0
VIIa Hepatoblastoma	6	2	0	**8**	*1.0*	2.4	4.5	1.6	-	2.1	**30.8**	75.0
VIIb Hepatic carcinomas	0	0	0	**0**	*-*	-	-	-	-	-	**-**	-
VIII Bone tumours	2	6	11	**19**	*1.7*	5.6	1.5	4.8	8.4	4.9	**73.5**	100.0
VIIIa Osteosarcoma	1	1	4	**6**	*2.0*	1.8	0.8	0.8	3.0	1.5	**23.0**	100.0
VIIIc Ewing	1	5	7	**13**	*1.6*	3.8	0.8	4.0	5.3	3.4	**50.5**	100.0
IX Soft tissue sarcomas	13	4	5	**22**	*1.0*	6.5	9.8	3.2	3.8	5.7	**84.3**	100.0
IXa Rhabdomyosarcoma	11	4	3	**18**	*1.0*	5.3	8.3	3.2	2.3	4.6	**69.1**	100.0
IXc Kaposi sarcoma	0	0	0	**0**	*-*	-	-	-	-	-	**-**	-
X Germ cell tumours	9	2	1	**12**	*1.4*	3.5	6.8	1.6	0.8	3.1	**45.9**	91.7
XI Carcinomas	6	3	4	**13**	*0.6*	3.8	4.5	2.4	3.0	3.4	**50.0**	100.0
Other and unspecified	0	0	0	**0**	*-*	-	-	-	-	-	**-**	-
TOTAL	182	77	81	**340**	* 1.3*	100.0	137.6	62.1	61.5	87.7	**1306.4**	93.5

Population (average annual)		
**South African Republic (2008–2012)**	**Males**	**Females**
0–4	2,803,199	2,758,689
5–9	2,425,244	2,397,887
10–14	2,361,743	2,279,847

**South African Republic: Black (2008–2012)**		
0–4	2,376,204	2,346,414
5–9	2,035,685	2,021,448
10–14	1,959,969	1,897,967

**South African Republic: White (2008–2012)**		
0–4	135,706	128,748
5–9	127,838	120,122
10–14	136,521	126,771

**Source:** Statistics South Africa. Mid-year population estimates. (Statistical release P0302)

#### 6.2.3 RSA: Eastern Cape Province Cancer Registry

**Figure:**
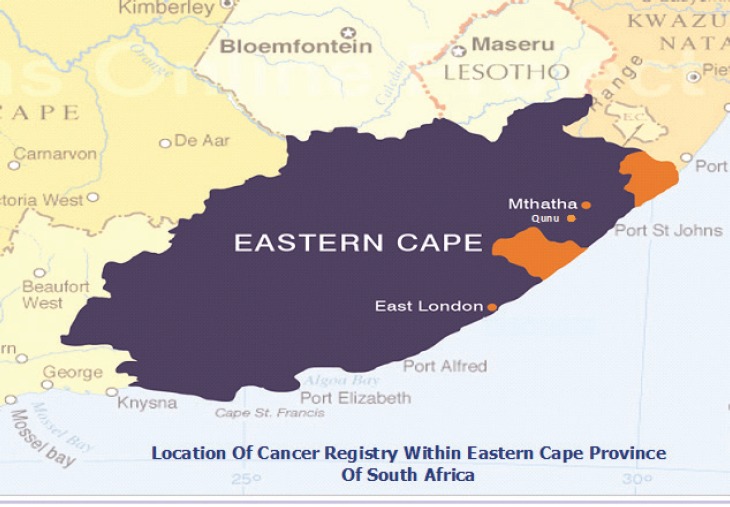
Map of the catchment area served by the cancer registry. Source unknown.

Eastern Cape Province Cancer Registry is population-based and was established in the 1980s as a special registry by the Programme on Mycotoxin and Experimental Carcinogenesis (PROMEC) of the South African Medical Research Council (SAMRC). Initially, the register was set up to monitor trends in the incidence and spatial geographical variations of oesophageal cancer in four magisterial areas of the former Transkei region of the Eastern Cape Province. These were Butterworth and Centane; the so-called high incidence areas and Bizana and Lusikisiki the low incidence areas then. In 1998, the registry expanded its scope to collect data on all cancers and expanded the geographic coverage to eight magisterial areas that include the initial four and Idutywa, Nqamakwe, Willowvale, and Flagstaff. These magisterial areas are in the municipalities of Ntabankulu, Mbizana, and Qaukeni in the north-eastern part of the former Transkei region and Mnquma and Mbashe in the south-western part of the region. The registry is funded mainly by the SAMRC.

The population covered by the registry at the most recent census 2011 was 1.1 million. Of which, 99% of the population are Black Africans who speak isiXhosa supporting both Christian and traditional norms and values.

The registry is collaborating with 15 hospitals that serve the area, including the pathology laboratory under the National Health Laboratory Services (NHLS) situated in Nelson Mandela Medical School at Mthatha. Hospitals in the registration area include: St Patrick’s & Greenville Hospitals (Bizana), Butterworth Hospital (Butterworth), Tafalofefe Hospital (Centane), Holy Cross, St Elizabeth & Bambisana Hospitals (Lusikisiki), and the Nqamakwe Health Centre (Nqamakwe).

Specialists such as paediatricians and oncologists are at referral regional hospital; Nelson Mandela Medical School and oncology radiation unit; Frere Hospital which serve the registration area. It is only at this level of treatment where patients receive specialised investigations and treatment. Cancer is rare in children sometimes cancers like leukaemia can be missed by a general medical doctor at local hospitals. It is therefore possible that cancer in children is under diagnosed in this population.

Both active and passive case finding methods are used. The active case finding system was set up by the registry manager utilising multiple sources. Collaborating hospitals located in eight magisterial areas are visited twice a year. During these visits, records are examined for all cancer patients treated in the facility and their details are abstracted for inclusion in the registry. The records perused include in-patients’ admission, treatment, transfer, discharge and death registers, midnight census records, and pathology reports. Case finding also extends to hospitals outside the registration area to minimise case loss.

Although the information on the death register is limited, the national death register is used to update the vital status of registered cases.

The CanReg-4 system was used for data management during the period for which data are presented.

#### Years presented

Data for a five-year period 2008–2012 are presented in the following table.

**Table 6.2.3.** South African Republic, Eastern Cape (2003–2012).

	NUMBER OF CASES				REL. FREQ.(%)		RATES PER MILLION					
	0-4	5-9	10-14	All	*M/F*	Overall	0-4	5-9	10-14	Crude	Cum. 0-14	%MV
I Leukaemia	2	8	4	**14**	*0.8*	12.4	1.4	5.4	2.6	3.2	**47.1**	42.9
Ia Lymphoid	0	1	1	**2**	*-*	1.8	-	0.7	0.7	0.5	**6.6**	100.0
Ib Acute myeloid	0	0	0	**0**	*-*	-	-	-	-	-	**-**	-
II Lymphoma	1	3	9	**13**	*0.6*	11.5	0.7	2.0	5.9	2.9	**43.2**	76.9
IIa Hodgkin	0	1	1	**2**	*1.0*	1.8	-	0.7	0.7	0.5	**6.6**	100.0
IIb NHL (except Burkitt)	0	1	5	**6**	*0.5*	5.3	-	0.7	3.3	1.4	**19.8**	100.0
IIc Burkitt	0	0	0	**0**	*-*	-	-	-	-	-	**-**	-
III Brain and spinal neoplasms	4	2	4	**10**	*0.7*	8.8	2.8	1.3	2.6	2.3	**34.1**	40.0
IVa Neuroblastoma	1	0	1	**2**	*1.0*	1.8	0.7	-	0.7	0.5	**6.8**	50.0
V Retinoblastoma	13	3	0	**16**	*4.3*	14.2	9.2	2.0	-	3.6	**56.2**	56.2
VIa Nephroblastoma	20	3	1	**24**	*0.8*	21.2	14.2	2.0	0.7	5.4	**84.4**	66.7
VII Hepatic tumours	1	0	1	**2**	*-*	1.8	0.7	-	0.7	0.5	**6.8**	100.0
VIIa Hepatoblastoma	1	0	0	**1**	*-*	0.9	0.7	-	-	0.2	**3.6**	100.0
VIIb Hepatic carcinomas	0	0	1	**1**	*-*	0.9	-	-	0.7	0.2	**3.3**	100.0
VIII Bone tumours	0	0	3	**3**	*0.5*	2.7	-	-	2.0	0.7	**9.9**	100.0
VIIIa Osteosarcoma	0	0	3	**3**	*0.5*	2.7	-	-	2.0	0.7	**9.9**	100.0
VIIIc Ewing	0	0	0	**0**	*-*	-	-	-	-	-	**-**	-
IX Soft tissue sarcomas	6	7	1	**14**	*1.3*	12.4	4.3	4.7	0.7	3.2	**48.1**	85.7
IXa Rhabdomyosarcoma	5	5	0	**10**	*4.0*	8.8	3.6	3.4	-	2.3	**34.5**	100.0
IXc Kaposi sarcoma	0	1	1	**2**	*-*	1.8	-	0.7	0.7	0.5	**6.6**	50.0
X Germ cell tumours	0	1	2	**3**	*2.0*	2.7	-	0.7	1.3	0.7	**9.9**	100.0
XI Carcinomas	2	1	1	**4**	*3.0*	3.5	1.4	0.7	0.7	0.9	**13.7**	100.0
Other and unspecified	3	1	4	**8**	*1.0*	7.1	2.1	0.7	2.6	1.8	**27.2**	-
TOTAL	53	29	31	**113**	* 1.1*	100.0	37.6	19.5	20.4	25.6	**387.4**	61.9

Population (average annual)		
	**Males**	**Females**
0–4	71,179	69,650
5–9	75,134	73,754
10–14	77,701	74,489
all (0–14)	224,014	217,893

**Source:** Population estimates for each individual year for the period, based on 2001 and 2011 census results.

Estimate for 2012 based on annual change 2001–2011 within 5-year age groups.

Source: Space−Time Research Web page: www.str.com.au

Butterworth, Flagstaff, Lusikisiki, Willowvale, Nqamakwe, Idutywa, Kentani/Centane and Bizana

### 6.3. West Africa

#### 6.3.1 Guinea: Registre de Cancer de Guinee

**Figure:**
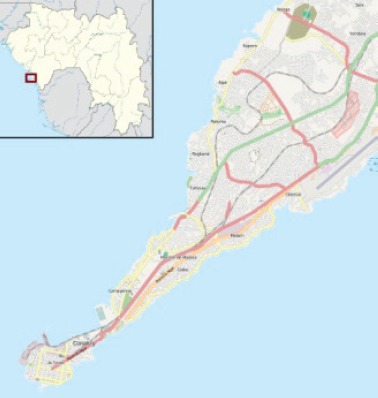
Map of the catchment area served by the cancer registry. Source unknown.

The cancer registry of Guinea was established in 1990 with the assistance of the International Agency for Research on Cancer (IARC). It is located in, and supported by, the Department of Pathology at the National Hospital of Donka, Conakry. It aims to be population based for the city of Conakry. A six-month duration of stay is used as the criterion to distinguish cancer patients who are usual residents of the city from temporary visitors (cases domiciled with their families for the purpose of receiving medical care).

The most recent census of the population was in 2014, at which time the population of the city was 1,667,864.

The main sources of information on cancer cases are the Hôpital National de Donka (which has all specialities, including oncological surgery), the Hôpital National Ignace Deen (471 beds - all specialities), and, the Hôpital Amitié Sino-Guinéen (Kipé), which has 120 beds, and includes services such as surgery and neurosurgery. Data are also collected from four community hospitals and several private clinics. There is little specialised oncology in Guinée, apart from a department of Oncological Surgery and Gynaecological Oncology in Hôpital de Donka. There is no service of radiotherapy in the country – patients are sent to Dakar for treatment. There are two laboratories of pathology in the city (Hôpital de Donka and Hôpital Amitié Sino-Guinéen). The former is a major source of information for the registry; data on cancer cases are retrieved from the request form, onto which the diagnosis has been written. There is no civil registration of death that entails certification of cause of death.

There was a Paediatric Oncology Unit created since 2008 in the paediatric ward of the Donka Hospital. This unit was approved by the Franco–African Paediatric Oncology Group (GFAOP) in 2012. But in 2015 a fire destroyed the Unit’s facilities and the Donka Hospital was closed for 2015–2016. Another paediatric oncology unit was set up by the Conseil en Santé de Kipé and was officially admitted to GALOP in 2012 with the sponsorship from Mali and Senegal. It will be functional when two paediatricians complete their training.

Case finding is entirely active, by medical students, as part of their course work (preparation of mini-theses). They visit the clinical services expected to generate cancer cases, and collect the information onto a Registration Form. None of the hospitals have central records departments. Medical records are kept in each service, although these may also have registers of admissions/discharges, with a simple diagnosis for each.

The registry uses the CANREG-4 system for data entry and management.

#### Years presented

Data for a 10-year period 2001–2010 are presented in the following table.

**Table 6.3.1.** Guinea, Conakry (2001–2010).

	NUMBER OF CASES				REL. FREQ.(%)		RATES PER MILLION					
	0-4	5-9	10-14	All	*M/F*	Overall	0-4	5-9	10-14	Crude	Cum. 0-14	%MV
I Leukaemia	2	0	1	**3**	*-*	1.8	0.8	-	0.8	0.5	**7.7**	100.0
Ia Lymphoid	0	0	0	**0**	*-*	-	-	-	-	-	**-**	-
Ib Acute myeloid	2	0	0	**2**	*-*	1.2	0.8	-	-	0.3	**3.9**	100.0
II Lymphoma	2	27	34	**63**	*1.7*	38.0	0.8	13.9	26.2	10.8	**204.1**	92.1
IIa Hodgkin	0	3	5	**8**	*1.0*	4.8	-	1.5	3.8	1.4	**26.9**	100.0
IIb NHL (except Burkitt)	1	6	10	**17**	*1.4*	10.2	0.4	3.1	7.7	2.9	**55.8**	100.0
IIc Burkitt	1	12	5	**18**	*1.6*	10.8	0.4	6.2	3.8	3.1	**52.0**	88.9
III Brain and spinal neoplasms	0	0	0	**0**	*-*	-	-	-	-	-	**-**	-
IVa Neuroblastoma	0	0	0	**0**	*-*	-	-	-	-	-	**-**	-
V Retinoblastoma	16	1	0	**17**	*3.2*	10.2	6.2	0.5	-	2.9	**33.7**	11.8
VIa Nephroblastoma	5	4	2	**11**	*0.8*	6.6	1.9	2.1	1.5	1.9	**27.7**	90.9
VII Hepatic tumours	1	1	2	**4**	*-*	2.4	0.4	0.5	1.5	0.7	**12.2**	50.0
VIIa Hepatoblastoma	1	0	0	**1**	*-*	0.6	0.4	-	-	0.2	**1.9**	100.0
VIIb Hepatic carcinomas	0	0	1	**1**	*-*	0.6	-	-	0.8	0.2	**3.8**	100.0
VIII Bone tumours	0	1	6	**7**	*2.5*	4.2	-	0.5	4.6	1.2	**25.7**	100.0
VIIIa Osteosarcoma	0	1	2	**3**	*-*	1.8	-	0.5	1.5	0.5	**10.3**	100.0
VIIIc Ewing	0	0	0	**0**	*-*	-	-	-	-	-	**-**	-
IX Soft tissue sarcomas	5	7	3	**15**	*1.5*	9.0	1.9	3.6	2.3	2.6	**39.2**	100.0
IXa Rhabdomyosarcoma	2	2	0	**4**	*1.0*	2.4	0.8	1.0	-	0.7	**9.0**	100.0
IXc Kaposi sarcoma	0	0	0	**0**	*-*	-	-	-	-	-	**-**	-
X Germ cell tumours	3	1	2	**6**	*0.5*	3.6	1.2	0.5	1.5	1.0	**16.1**	100.0
XI Carcinomas	5	10	7	**22**	*1.2*	13.3	1.9	5.1	5.4	3.8	**62.3**	100.0
Other and unspecified	7	3	8	**18**	*1.0*	10.8	2.7	1.5	6.2	3.1	**52.1**	11.1
TOTAL	46	55	65	**166**	* 1.6*	100.0	17.9	28.3	50.0	28.5	**480.8**	76.5

Population (average annual)		
	**Males**	**Females**
0–4	128,774	128,527
5–9	97,811	96,775
10–14	65,819	64,138
all (0–14)	292,404	289,439

**Source:** Population estimates for each individual year for the period.

Estimates 2001−2010 assume constant rate of growth within age groups between 1996 and 2014 censuses.

Age distributions by sex for the 1996 census were applied to the total population by sex for the 2014 census.

Source: Ministere du plan, Institut National de la Statistique (INS)

Bureau Central du Recensement (BCR)

Troisieme recensement general de la population et de l´habitation. Resultats préliminaires

#### 6.3.2 Registre de Cancer de Mali

**Figure:**
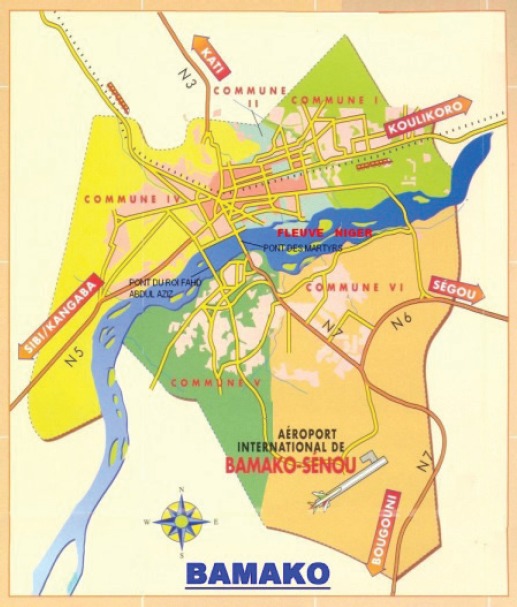
Map of the catchment area served by the cancer registry. Source unknown.

Registre de Cancer de Mali was established in 1986. It is based in the Department of Pathology of the Centre Hospitalier Universitaire (CHU) de Point G, in the capital city of Bamako. Since its inception, the registry has collected information on cases of cancer from all possible sources within the city, and has aimed to be population based for Bamako. The work of the registry is entirely financed via the Department of Pathology.

The most recent census was in 2009, at which time, the population was 1,810,366. More recent estimates can be made, projecting forward the annual growth rates observed between 1999 and 2009. The city consists of 6 communes.

The Head of the Pathology Department oversees the activities of the registry. The data collection is active. There are four major hospitals in and the city (The University Hospitals (CHU) of Point G and Gabriel Touré, the Hôpital du Mali and the Hôpital du Kati). There are in addition some specialist services for ophthalmology, dermatology, and dental/ENT, as well as some six small district hospitals.

There are specialised cancer treatment services in CHU de Point G (medical oncology and the only paediatric oncology unit in Mali) and Hôpital du Mali, which houses a service of radiotherapy. There is a specialist Haematology Clinic in the CHU of Point G.

There are four laboratories de pathology. The main one is located in Point G with four qualified pathologists, which does the great bulk of the work in Bamako (and nationally). The laboratory at Hôpital du Mali deals with some of the specimens from that hospital. A few clinicians send specimens to overseas labs.

All deaths must be certified at the civil registry in order to receive a burial permit. The registry uses death certificates as a source of information. Deaths apparently from cancer are matched with the registry database. It is difficult to follow back unmatched cases to hospitals: they are therefore registered as DCO cases.

Data entry and management is carried out using CanReg 4.

#### Years presented

Data for a nine-year period 2006–2014 are presented in the table below.

[All 159 cases in the ‘Other and Unspecified’ category were without a histological diagnosis, although 43 were cancers of the eye (probably retinoblastomas) and 28 cancers of the kidney (probably nephroblastomas].

**Table 6.3.2.** Mali, Bamako (2006–2014).

	NUMBER OF CASES				REL. FREQ.(%)		RATES PER MILLION					
	0-4	5-9	10-14	All	*M/F*	Overall	0-4	5-9	10-14	Crude	Cum. 0-14	%MV
I Leukaemia	6	27	24	**57**	*1.4*	7.1	2.3	12.3	12.5	8.5	**135.4**	73.7
Ia Lymphoid	3	20	16	**39**	*1.4*	4.9	1.2	9.1	8.3	5.8	**92.9**	66.7
Ib Acute myeloid	0	3	4	**7**	*0.8*	0.9	-	1.4	2.1	1.0	**17.2**	100.0
II Lymphoma	22	72	59	**153**	*1.9*	19.1	8.5	32.8	30.6	22.8	**359.7**	96.1
IIa Hodgkin	3	16	19	**38**	*3.8*	4.7	1.2	7.3	9.9	5.7	**91.6**	97.4
IIb NHL (except Burkitt)	5	17	13	**35**	*2.2*	4.4	1.9	7.7	6.7	5.2	**82.1**	94.3
IIc Burkitt	9	27	14	**50**	*1.3*	6.2	3.5	12.3	7.3	7.5	**115.2**	100.0
III Brain and spinal neoplasms	6	9	3	**18**	*1.6*	2.2	2.3	4.1	1.6	2.7	**39.9**	44.4
IVa Neuroblastoma	10	5	0	**15**	*1.5*	1.9	3.9	2.3	-	2.2	**30.7**	80.0
V Retinoblastoma	112	20	9	**141**	*1.2*	17.6	43.2	9.1	4.7	21.0	**285.2**	82.3
VIa Nephroblastoma	58	41	8	**107**	*1.1*	13.4	22.4	18.7	4.2	15.9	**226.2**	87.9
VII Hepatic tumours	8	3	2	**13**	*1.6*	1.6	3.1	1.4	1.0	1.9	**27.5**	46.2
VIIa Hepatoblastoma	7	1	0	**8**	*1.0*	1.0	2.7	0.5	-	1.2	**15.8**	62.5
VIIb Hepatic carcinomas	0	1	1	**2**	*1.0*	0.2	-	0.5	0.5	0.3	**4.9**	50.0
VIII Bone tumours	14	9	20	**43**	*0.6*	5.4	5.4	4.1	10.4	6.4	**99.4**	48.8
VIIIa Osteosarcoma	2	4	7	**13**	*0.6*	1.6	0.8	1.8	3.6	1.9	**31.1**	92.3
VIIIc Ewing	1	1	0	**2**	*1.0*	0.2	0.4	0.5	-	0.3	**4.2**	100.0
IX Soft tissue sarcomas	17	11	23	**51**	*2.2*	6.4	6.6	5.0	11.9	7.6	**117.6**	92.2
IXa Rhabdomyosarcoma	11	5	6	**22**	*4.5*	2.7	4.2	2.3	3.1	3.3	**48.2**	81.8
IXc Kaposi sarcoma	0	0	1	**1**	*-*	0.1	-	-	0.5	0.1	**2.6**	100.0
X Germ cell tumours	5	4	5	**14**	*1.0*	1.7	1.9	1.8	2.6	2.1	**31.7**	71.4
XI Carcinomas	4	10	16	**30**	*1.5*	3.7	1.5	4.6	8.3	4.5	**72.0**	96.7
Other and unspecified	76	45	38	**159**	*1.4*	19.9	29.3	20.5	19.7	23.7	**347.9**	19.5
TOTAL	338	256	207	**801**	* 1.4*	100.0	130.5	116.7	107.4	119.4	**1773.1**	70.3

Population (average annual)		
	**Males**	**Females**
0–4	145,540	142,210
5–9	121,108	122,697
10–14	98,422	115,644
all (0–14)	365,070	380,551

**Source:** Population estimates based on the censuses of 1999 and 2009 (institut National de Statistique)

Intercensal estimates assume a constant rate of growth within 5-year age groups

Populations for 2010–2014 were estimated assuming that the same rates of growth were continued.

#### 6.3.3 Registre Des Cancers Du Niger

**Figure:**
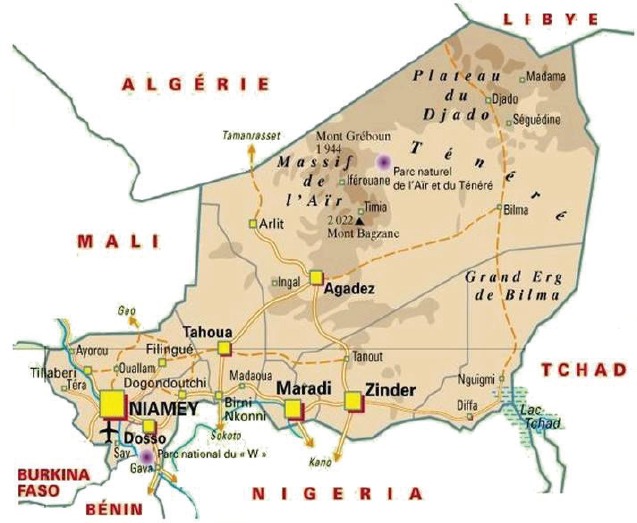
Map of the catchment area served by the cancer registry. Source unknown.

The Registre des cancers du Niger was founded in 1992, in the Faculté des Sciences de la Santé of the University of Niamey. It is located in the Department of Pathology at the University Hospital. This department is a referral centre for pathology services for the whole country. Nevertheless, the registry was designed to be population based with complete recording of all cancer cases diagnosed among the population of the capital city, Niamey. Niger comprises eight administrative subdivisions, three in the capital city.

In 2010, the population of Niamey was estimated to be 1,222,066. The definition of ‘usual resident’ of Niamey is six months’ residence in the city.

The most important source of information is the Department of Pathology, which provides histopathology and cytology services for the whole country. Although some specimens are sent out of the country, the registry receives copies of reports of all cancer cases diagnosed by the pathology services in the city, including biochemical tests such as human chorionic gonadotrophin (HCG), prostate-specific antigen (PSA), and alpha-foetoprotein.

Case finding is through actively searching for cases in the hospital services in the city where cancer might be diagnosed. These include, especially, the National Hospital, the University Hospital and the main maternity hospital. Visits are made to the major services (surgery, urology, medicine, gynaecology, paediatrics, and the biology laboratory) once every two weeks. Other clinics visited include maternal and child health clinics and occasionally some private clinics with clinicians to collect biopsies.

No cause of death is recorded on death certificates in Niger.

There is not a specific service of Paediatric Oncology. Children with cancer are referred to medical oncologists, surgical oncologists, or haematology oncologists as appropriate.

The registration process is carried out using the CANREG-4 software.

#### Years presented

Data of a four-year period 2006–2009 are presented in the table below.

[All 46 cases in the ‘Other and Unspecified’ category were without a histological diagnosis, although 14 were cancers of the skin and four cancers of the kidney (probably nephroblastomas].

**Table 6.3.3.** Niger, Niamey (2001–2009).

	NUMBER OF CASES				REL. FREQ.(%)		RATES PER MILLION					
	0-4	5-9	10-14	All	*M/F*	Overall	0-4	5-9	10-14	Crude	Cum. 0-14	%MV
!I Leukaemia	0	9	6	**15**	*4.0*	8.6	-	8.1	5.8	4.5	**69.6**	6.7
Ia Lymphoid	0	8	2	**10**	*4.0*	5.7	-	7.2	1.9	3.0	**45.6**	10.0
Ib Acute myeloid	0	0	0	**0**	*-*	-	-	-	-	-	**-**	-
II Lymphoma	7	27	22	**56**	*1.9*	32.0	5.8	24.2	21.4	16.8	**257.4**	78.6
IIa Hodgkin	0	3	8	**11**	*4.5*	6.3	-	2.7	7.8	3.3	**52.4**	90.9
IIb NHL (except Burkitt)	2	6	1	**9**	*0.8*	5.1	1.7	5.4	1.0	2.7	**40.1**	88.9
IIc Burkitt	3	15	9	**27**	*2.4*	15.4	2.5	13.5	8.8	8.1	**123.6**	77.8
III Brain and spinal neoplasms	3	2	1	**6**	*5.0*	3.4	2.5	1.8	1.0	1.8	**26.4**	-
IVa Neuroblastoma	1	0	0	**1**	*-*	0.6	0.8	-	-	0.3	**4.2**	100.0
V Retinoblastoma	10	0	0	**10**	*1.0*	5.7	8.4	-	-	3.0	**41.8**	90.0
VIa Nephroblastoma	1	3	0	**4**	*0.3*	2.3	0.8	2.7	-	1.2	**17.6**	100.0
VII Hepatic tumours	0	1	0	**1**	*-*	0.6	-	0.9	-	0.3	**4.5**	-
VIIa Hepatoblastoma	0	0	0	**0**	*-*	-	-	-	-	-	**-**	-
VIIb Hepatic carcinomas	0	0	0	**0**	*-*	-	-	-	-	-	**-**	-
VIII Bone tumours	3	4	15	**22**	*1.4*	12.6	2.5	3.6	14.6	6.6	**103.4**	45.5
VIIIa Osteosarcoma	0	1	10	**11**	*2.7*	6.3	-	0.9	9.7	3.3	**53.1**	90.9
VIIIc Ewing	0	0	0	**0**	*-*	-	-	-	-	-	**-**	-
IX Soft tissue sarcomas	2	2	3	**7**	*0.8*	4.0	1.7	1.8	2.9	2.1	**31.9**	85.7
IXa Rhabdomyosarcoma	2	1	2	**5**	*1.5*	2.9	1.7	0.9	1.9	1.5	**22.6**	80.0
IXc Kaposi sarcoma	0	0	0	**0**	*-*	-	-	-	-	-	**-**	-
X Germ cell tumours	0	1	3	**4**	*-*	2.3	-	0.9	2.9	1.2	**19.1**	50.0
XI Carcinomas	1	1	1	**3**	*2.0*	1.7	0.8	0.9	1.0	0.9	**13.5**	100.0
Other and unspecified	17	16	13	**46**	*1.9*	26.3	14.2	14.4	12.6	13.8	**206.0**	6.5
TOTAL	45	66	64	**175**	* 1.7*	100.0	37.6	59.2	62.2	52.4	**795.4**	47.4

Population (average annual)		
	**Males**	**Females**
0–4	67,012	65,952
5–9	61,477	62,346
10–14	55,924	58,315
all (0–14)	184,413	186,613

**Source:** Structure par âge et par sexe de la population de la région de Niamey sur la période 2001–2012

Source: Institut National de la Statistique

#### 6.3.4 Nigeria: Ibadan Cancer Registry

**Figure:**
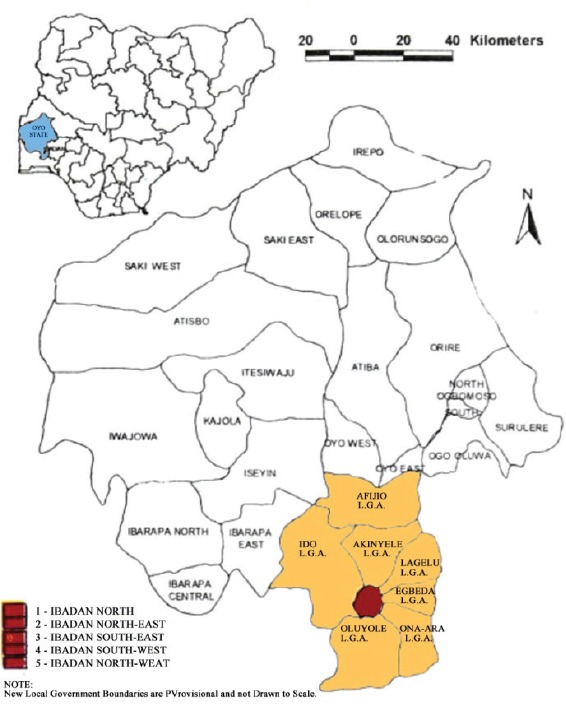
Map of the catchment area served by the cancer registry. Source unknown.

The Ibadan Cancer Registry (IBCR) was set up in April 1960 and is now based in the Pathology Department of the University College Hospital (UCH) in Ibadan. The registry is funded in part by both the University College Hospital and the College of Medicine of the University of Ibadan, as well as by grant support from the IARC. The Registry is led by its Principal Investigator (Consultant Pathologist) and is staffed with six members.

Ibadan is the capital of Oyo State. According to the 2006 population census figures, the estimated figure for the catchment area of the registry (comprising 11 local government areas) was 2,549,265. Oyo State is homogenous, mainly inhabited by the Yoruba ethnic group who are primarily agrarian. Ibadan however includes a collection of Hausa/Fulani and persons of Igbo and Efik/Ibibio extraction constituting about 10% of the population. There are about 55% Muslims and 45% Christians. Residents are defined as persons who have been living in the relevant area for at least one year.

The registry collects cases from 11 local governments within Ibadan and its environs. Apart from these, the registry also receives cases from out of town facilities like the Baptist Medical Centre, Ogbomosho (Ogbomosho North local Government Area), and the Ladoke Akintola University of Technology Teaching Hospital, Oshogbo. Decreasingly lower numbers still come from the Obafemi Awolowo University Teaching Hospital, Ile-Ife (Ife North Local Government area) and many of the state and privately-owned hospitals.

In the registry coverage area, paediatric oncology services available include surgery, chemotherapy, radiation therapy, and palliative care. These are mostly available in the teaching hospitals. Palliative care takes place at a hospice located within the hospital premises.

Specialist cancer treatment services are available for cancer care in the UCH including radiotherapy. The Pathology Laboratory of UCH provides histological, fine-needle aspiration cytology (FNAC) and other diagnostic immunohistochemical and cytological services and is a major source of information for the registry. Registry staff pay regular visits to the Haematology Department to retrieve information on the reports of haematological malignancies. In addition, there are two government state hospitals (Adeoyo Hospital and Ring Road Hospital) and several private and mission hospitals that provide general medical, gynaecological and paediatric services. There are now two other private pathology laboratories in the registry area.

The Ibadan Cancer Registry uses a proactive method for data collection. Visits are made to these sources, where the registry staff scrutinise the records kept in the medical records department, and registers of individual departments concerned with diagnosis and treatment of cancers, to identify and abstract information on cases of cancers, diagnosed by all methods, among residents of the registry region. The death registration system is inadequate and incomplete in the state; except for some of the death certificates written at UCH.

The CanReg computer system has been used for data recording and management since 1997.

#### Years presented

Data of a four-year period 2006–2009 are presented in the table below.

**Table 6.3.4.** Nigeria, Ibadan (2003–2012).

	NUMBER OF CASES				REL. FREQ.(%)		RATES PER MILLION					
	0-4	5-9	10-14	All	*M/F*	Overall	0-4	5-9	10-14	Crude	Cum. 0-14	%MV
I Leukaemia	4	4	9	**17**	*1.8*	4.4	2.3	2.5	6.3	3.5	**55.0**	23.5
Ia Lymphoid	1	1	4	**6**	*1.0*	1.6	0.6	0.6	2.8	1.2	**19.8**	16.7
Ib Acute myeloid	1	3	2	**6**	*5.0*	1.6	0.6	1.9	1.4	1.2	**19.1**	33.3
II Lymphoma	9	49	47	**105**	*1.4*	27.1	5.1	30.3	32.7	21.8	**340.3**	66.7
IIa Hodgkin	0	3	5	**8**	*7.0*	2.1	-	1.9	3.5	1.7	**26.6**	50.0
IIb NHL (except Burkitt)	2	8	18	**28**	*3.0*	7.2	1.1	4.9	12.5	5.8	**93.0**	89.3
IIc Burkitt	6	34	20	**60**	*1.0*	15.5	3.4	21.0	13.9	12.5	**191.6**	55.0
III Brain and spinal neoplasms	11	5	5	**21**	*1.1*	5.4	6.2	3.1	3.5	4.4	**64.1**	85.7
IVa Neuroblastoma	5	3	1	**9**	*0.3*	2.3	2.8	1.9	0.7	1.9	**26.9**	88.9
V Retinoblastoma	52	5	0	**57**	*0.9*	14.7	29.5	3.1	-	11.8	**163.1**	70.2
VIa Nephroblastoma	24	3	6	**33**	*0.8*	8.5	13.6	1.9	4.2	6.8	**98.3**	72.7
VII Hepatic tumours	3	1	6	**10**	*9.0*	2.6	1.7	0.6	4.2	2.1	**32.5**	50.0
VIIa Hepatoblastoma	2	0	0	**2**	*-*	0.5	1.1	-	-	0.4	**5.7**	50.0
VIIb Hepatic carcinomas	1	1	6	**8**	*7.0*	2.1	0.6	0.6	4.2	1.7	**26.8**	50.0
VIII Bone tumours	1	2	11	**14**	*1.8*	3.6	0.6	1.2	7.6	2.9	**47.3**	78.6
VIIIa Osteosarcoma	0	2	8	**10**	*2.3*	2.6	-	1.2	5.6	2.1	**34.0**	80.0
VIIIc Ewing	1	0	0	**1**	*-*	0.3	0.6	-	-	0.2	**2.8**	100.0
IX Soft tissue sarcomas	21	20	17	**58**	*1.8*	15.0	11.9	12.4	11.8	12.0	**180.5**	75.9
IXa Rhabdomyosarcoma	18	13	10	**41**	*1.6*	10.6	10.2	8.0	7.0	8.5	**126.0**	82.9
IXc Kaposi sarcoma	0	2	1	**3**	*2.0*	0.8	-	1.2	0.7	0.6	**9.7**	100.0
X Germ cell tumours	13	0	1	**14**	*0.3*	3.6	7.4	-	0.7	2.9	**40.4**	92.9
XI Carcinomas	11	17	18	**46**	*1.1*	11.9	6.2	10.5	12.5	9.5	**146.3**	76.1
Other and unspecified	2	1	0	**3**	*2.0*	0.8	1.1	0.6	-	0.6	**8.8**	100.0
TOTAL	156	110	121	**387**	* 1.2*	100.0	88.6	68.0	84.1	80.3	**1203.4**	71.1

Population (average annual)		
	**Males**	**Females**
0–4	71,263	69,625
5–9	65,522	63,926
10–14	57,436	57,642
all (0–14)	194,221	191,192

**Source:** Population estimates for 2006 for Ibadan city.

Federal Republic of Nigeria 2006 Population and Housing Census

Priority Table Volume IV

Published by National Population Commission Abuja, Nigeria April, 2010

#### 6.3.5 Gambia National Cancer Registry

**Figure:**
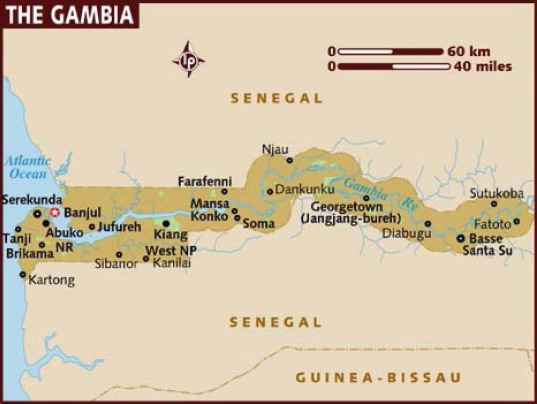
Map of the catchment area served by the cancer registry. Source unknown.

The Gambia National Cancer Registry (GNCR) was initially established in 1986 as part of the Gambia Hepatitis Intervention Study (GHIS) to record data on the pattern of cancer occurrence in The Gambia. This collaborative project, involving IARC, The Gambia government and the UK Medical Research Council, was initially supported by the Government of Italy through its Ministry of Foreign Affairs and the Swedish Medical Research Council. The registry is wholly funded by IARC through the Gambia Hepatitis Intervention Study.

According to the 2010 revision of the World Population Prospects, the total population of The Gambia was 1,728,000.

The main source of the data is from four Government hospitals, the main ones being the EFSTH in Banjul, the AFPRC hospital in Farafenni on the north bank, Bwiam hospital in the West Coast region, and Bansang Hospital in the eastern region of the country which serves mainly a rural population. These hospitals are the major referral centres for the various government dispensaries and health centres, which are evenly located around the country. They provide general medical and laboratory services. The MRC clinic in Fajara is also a major referral centre, with three out-reach stations in rural areas; this institution has a broad-based laboratory research facility, and since 2011 has been the national referral centre for all patients with suspected liver disease. Data is also obtained from the Prevention of Liver Fibrosis and Cancer in Africa (PROLIFICA) project database, a 5-year EU-funded study involving The Gambia and 2 other West African countries. A Cuban haematologist has recently set up a clinic for paediatric haematology.

Registration of death is incomplete in The Gambia. Copies of certificates mentioning cancer are obtained from the registration office. ‘Death Certificate Only’ cases (those for which no diagnostic information could be found from any other source) are not included in the database.

Case finding is entirely active, by trained register officer. They visit the clinical services where is expected to generate cancer cases, and collect the information onto a standard registration form. They cross check these against the ward and the central medical records, in addition to registers of admissions/discharges.

Data entry and management uses the CanReg-5 software.

#### Years presented

Data for a 10-year period 2002–2011 are presented in the table below.

**Table 6.3.5.** The Gambia (2002–2011).

	NUMBER OF CASES				REL. FREQ.(%)		RATES PER MILLION					
	0-4	5-9	10-14	All	*M/F*	Overall	0-4	5-9	10-14	Crude	Cum. 0-14	%MV
I Leukaemia	4	9	5	**18**	*3.5*	9.3	1.5	3.9	2.5	2.6	**39.3**	22.2
Ia Lymphoid	1	5	0	**6**	*2.0*	3.1	0.4	2.1	-	0.9	**12.6**	50.0
Ib Acute myeloid	0	0	0	**0**	*-*	-	-	-	-	-	**-**	-
II Lymphoma	16	30	27	**73**	*1.7*	37.6	5.9	12.9	13.6	10.4	**162.0**	58.9
IIa Hodgkin	0	3	2	**5**	*-*	2.6	-	1.3	1.0	0.7	**11.5**	80.0
IIb NHL (except Burkitt)	1	3	7	**11**	*1.8*	5.7	0.4	1.3	3.5	1.6	**25.9**	81.8
IIc Burkitt	7	10	5	**22**	*1.2*	11.3	2.6	4.3	2.5	3.1	**47.0**	50.0
III Brain and spinal neoplasms	0	0	0	**0**	*-*	-	-	-	-	-	**-**	-
IVa Neuroblastoma	1	2	0	**3**	*2.0*	1.5	0.4	0.9	-	0.4	**6.1**	33.3
V Retinoblastoma	23	1	1	**25**	*0.7*	12.9	8.5	0.4	0.5	3.6	**47.0**	76.0
VIa Nephroblastoma	4	1	0	**5**	*0.7*	2.6	1.5	0.4	-	0.7	**9.5**	60.0
VII Hepatic tumours	9	7	11	**27**	*2.4*	13.9	3.3	3.0	5.5	3.8	**59.3**	-
VIIa Hepatoblastoma	0	0	0	**0**	*-*	-	-	-	-	-	**-**	-
VIIb Hepatic carcinomas	2	4	1	**7**	*1.3*	3.6	0.7	1.7	0.5	1.0	**14.8**	-
VIII Bone tumours	0	2	6	**8**	*1.0*	4.1	-	0.9	3.0	1.1	**19.4**	75.0
VIIIa Osteosarcoma	0	0	3	**3**	*0.5*	1.5	-	-	1.5	0.4	**7.6**	66.7
VIIIc Ewing	0	0	0	**0**	*-*	-	-	-	-	-	**-**	-
IX Soft tissue sarcomas	3	4	3	**10**	*1.5*	5.2	1.1	1.7	1.5	1.4	**21.7**	90.0
IXa Rhabdomyosarcoma	2	0	3	**5**	*0.7*	2.6	0.7	-	1.5	0.7	**11.2**	100.0
IXc Kaposi sarcoma	0	1	0	**1**	*-*	0.5	-	0.4	-	0.1	**2.1**	100.0
X Germ cell tumours	1	1	0	**2**	*1.0*	1.0	0.4	0.4	-	0.3	**4.0**	100.0
XI Carcinomas	3	6	0	**9**	*2.0*	4.6	1.1	2.6	-	1.3	**18.4**	100.0
Other and unspecified	3	4	7	**14**	*2.5*	7.2	1.1	1.7	3.5	2.0	**31.8**	42.9
TOTAL	67	67	60	**194**	* 1.6*	100.0	24.7	28.8	30.2	27.6	**418.6**	52.6

Population (average annual)		
	**Males**	**Females**
0–4	136,978	134,511
5–9	117,184	115,562
10–14	99,399	98,977
all (0–14)	353,561	349,050

**Source:** Population estimates for each individual year for the period.

Source: UN, World Population Prospects, the 2012 revision.

## 7. Summary table (all cancers)

The table below summarises the number of cases recorded in each of the participating registries (with sex ratio and incidence rates (and standard errors) for all cancers). The results for the individual cancers are presented in the following chapter (8. Results and Discussion – by type of cancer).

**Table 7.**
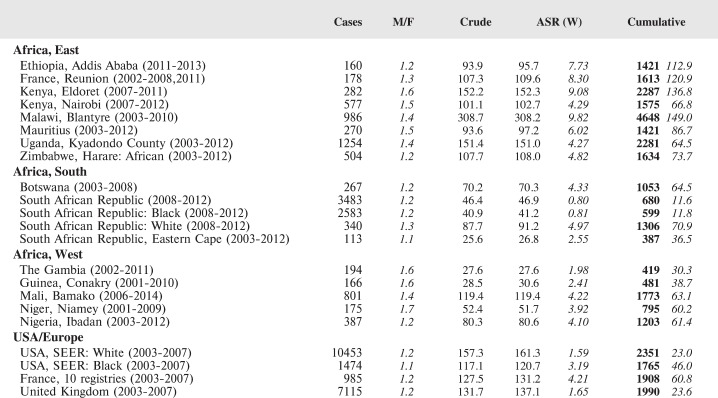
Crude, age-standardized (world) and cumulative (0–14) incidence Rates (per million) and standard errors – total.

## 8. Results and discussion – by type of cancer

This section provides a summary and discussion of the results for each of the 11 groups of cancers in the International Classification of Childhood Cancers.

For each group, and for the major subgroups within them, we provide a table showing, for each registry, the number of cases registered, the sex ratio, and the incidence rates (crude, age standardised, and cumulative), for ages 0–14. The standard errors of the age standardised and cumulative rates are shown in italic (Chapter 5).

To facilitate comparison, the cumulative rates are plotted as bar charts, with the bars for the registries in East Africa in red (stippled for the island populations of Mauritius and Reunion), blue for registries in Southern Africa, and green for those in West Africa. The cumulative incidence is shown as grey bars for the period 2003-2007 in four comparison populations (whenever available): for the SEER registries of the United States (black and white populations), for the United Kingdom, and for 10 cancer registries in France (data from Cancer Incidence in Five Continents, Volume X).

For those registries with 10 or more cases of a given cancer group (or subgroup), the age-specific incidence rates are shown for three age groups (0–4; 5–9; 10–14) as vertical bars, using the same colour coding as above.

For each group (or subgroup), we provide a brief commentary on the observations, highlighting features of interest, and discussing them in the light of other published work, and aetiological factors that might be relevant to them.

### 8.1. Leukaemias

**Table 8.1.**
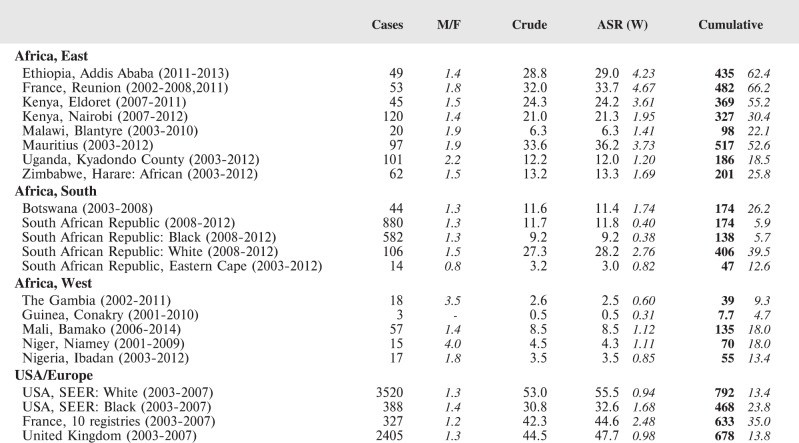
Crude, age-standardised (world) and cumulative (0-14) incidence rates (per million) and standard errors – leukaemias.

**Figure 8.1.**
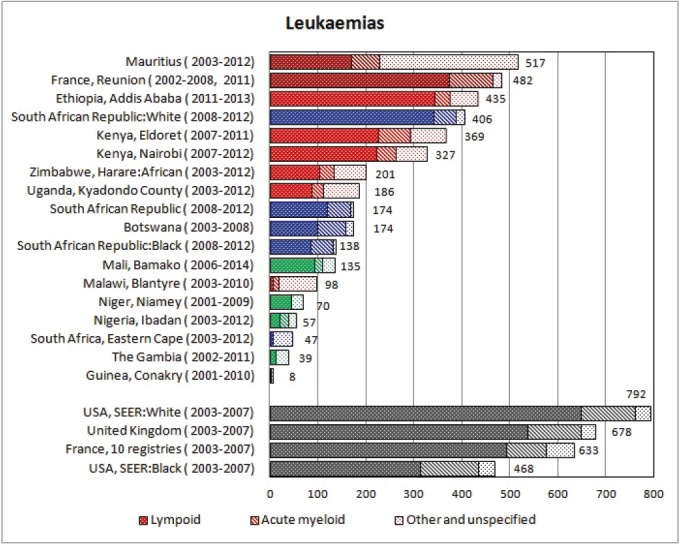
Cumulative (0-14) incidence rate – leukaemias: lymphoid, acute myeloid, and others.

#### 8.1.1 Lymphoid leukaemia

**Table 8.1.1.**
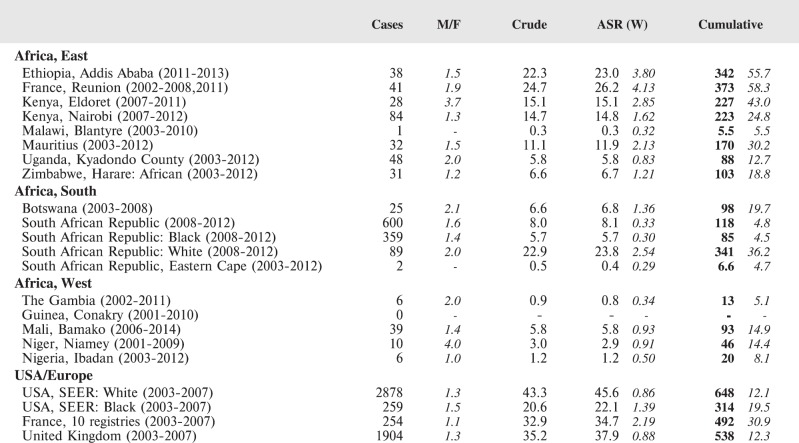
Crude, age-standardised (world) and cumulative (0-14) incidence rates (per million) and standard errors – lymphoid leukaemia.

**Figure 8.1.1a.**
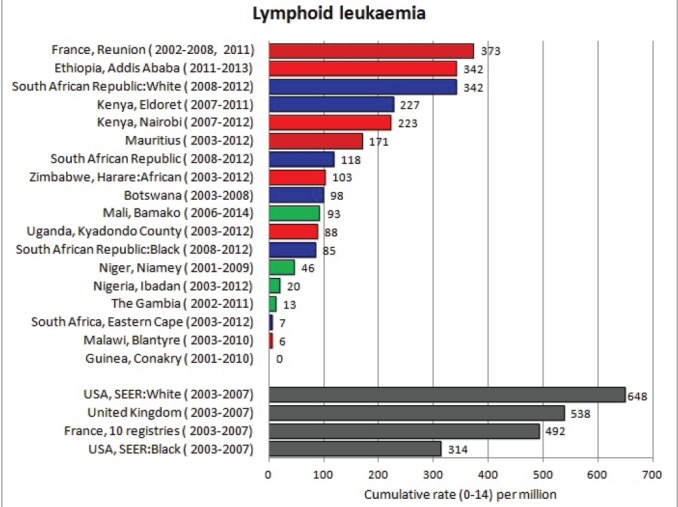
Cumulative (0–14) incidence rate – lymphoid leukaemia.

**Figure 8.1.1b.**
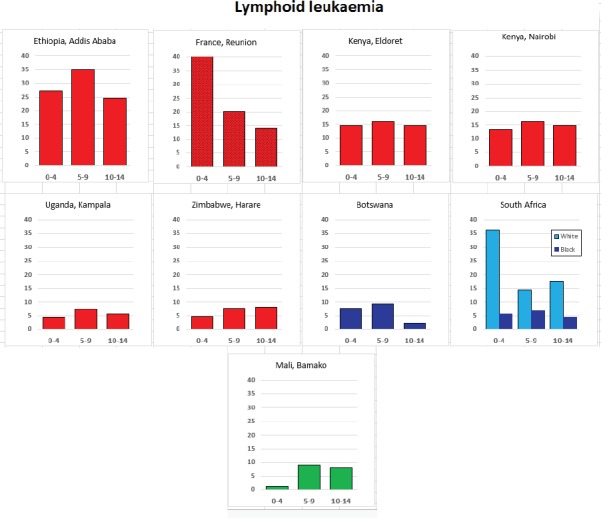
Age-specific histogram – lymphoid leukaemia.

#### 8.1.2 Acute myeloid leukaemia

**Table 8.1.2.**
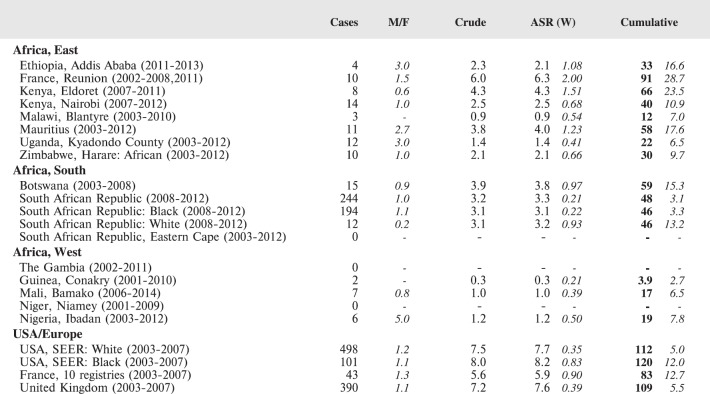
Crude, age-standardised (world) and cumulative (0–14) incidence rates (per million) and standard errors – Acute myeloid leukaemia.

**Figure 8.1.2a.**
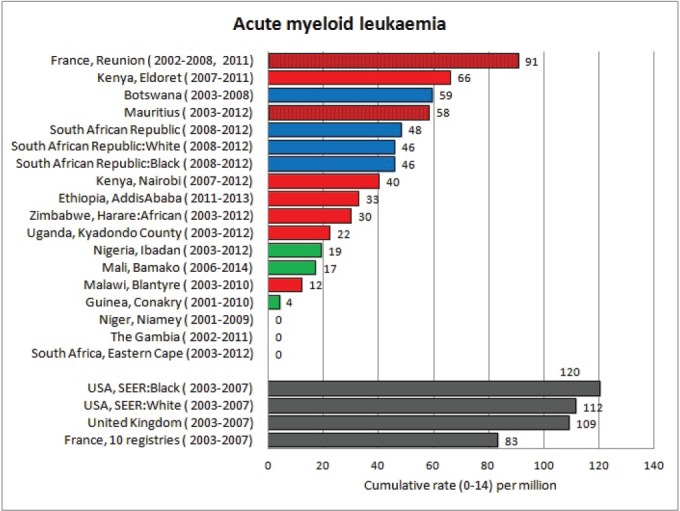
Cumulative (0-14) incidence rate – acute myeloid leukaemia.

**Figure 8.1.2b.**
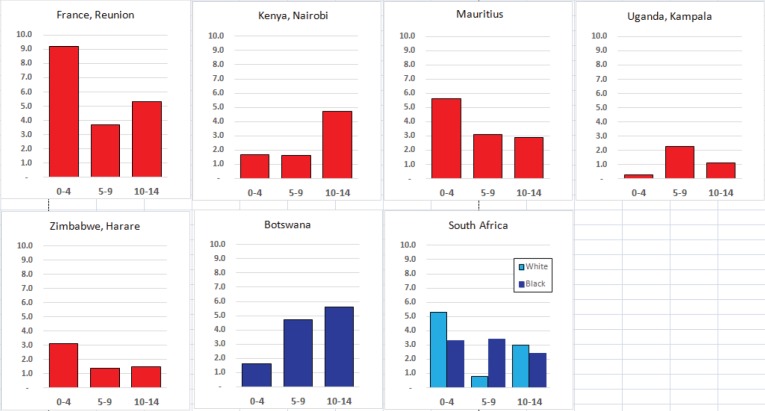
Age-specific histogram – acute myeloid leukaemia.

The International Classification of Childhood Cancer [8] separates leukaemias into five groups: lymphoid leukaemias (Ia), acute myeloid leukaemias (Ib), chronic myeloproliferative diseases, including chronic myeloid leukaemias (Ic), unspecified or combined types (Ie), and a separate group (Id) for refractory anaemia, myelodysplastic syndrome, and other myeloproliferative diseases. The first two groups comprise more than 95% of childhood leukaemias, at least in Europe [12], so analysis has been confined to these two groups.

In white populations of Europe, the Americas and Oceania, and also in much of eastern Asia, around a third of all childhood cancers are leukaemias. Cumulative rates in the three white populations in Table 8.1 are 600–800 per million.

There are several problems in interpreting the results from Africa presented in this volume. In particular, a major concern is that low incidence rates – especially of acute lymphoid leukaemias – may be the consequence of under-diagnosis and underreporting. Oncological haematology and clinical haematology services in general are few in Africa. As a consequence, diagnoses may be missed by internists, because the common clinical presentation of childhood leukaemia – fever, lymphadenopathy and anaemia – is easy to confuse with other common conditions in paediatric practice in the tropics. The diagnosis may be missed on examination of blood smears, where blast cells may be mistaken for the activated lymphocytes common in children with malaria. Finally, children with the above symptoms (particularly the very young) may not be brought to medical attention before the rapid evolution of the disease has caused death [13]. In addition to this, cancer registries in Africa may have less efficient methods of detecting cases diagnosed by blood smears or cytology than cancers identified through histopathology diagnoses. Some of the problems in individual registries have been alluded to in the sections describing the results from each centre.

Interpretation of the results from Africa with respect to specific types of leukaemia is also complicated by the fact that, from several registries, a substantial fraction of cases fall into the ‘Other and Unspecified’ group (e.g. more than half of the leukaemia cases from Mauritius and Blantyre (Malawi), and around 40% from Kampala/Kyadondo (Uganda) and Harare (Zimbabwe)).

Nevertheless, even taking these factors into account, the results presented are consistent with other reports in suggesting that incidence rates – particularly of acute lymphoblastic leukaemia, are low in African children.

Lymphoblastic neoplasms are derived from immature (precursor) lymphoid cells that are committed to one of the two major lymphoid differentiation pathways: B (bone marrow derived) or T (thymus derived). B lymphoblastic and T lymphoblastic precursor cells are collectively referred to as *acute lymphoblastic leukaemia/ lymphoma,* depending upon whether or not neoplastic blast cells are present in the bone marrow at the time of presentation. The cellular morphology, however, is identical.

Acute lymphoblastic leukaemia (ALL) comprises 75–80% of all leukaemias in populations of European origin, with cumulative rates generally in the range 500–650 per million. 75% of cases of ALL occur in children less than six years of age, and there is a marked peak in incidence at age 2–3 years of age [12]. This peak represents precursor B-cell acute lymphocytic leukaemia (it is not present in pre-T cell leukaemias), and its emergence with improving socioeconomic status in many countries worldwide has known for many years, since the early observations of Court Brown and Doll (1961) [14] and Miller (1977) [15]. This pattern in populations with a rising prosperity (and decreasing rates of infectious disease), together with current notions on the origin of the disease suggest a role for secular and demographic changes in child bearing and (lack of) stimulation of the immune system by infection [12].

In black children in the United States, leukaemia accounts for about a quarter of all childhood cancer; the incidence of ALL is only half that among whites, largely because of a much reduced early childhood peak [16]. In the United Kingdom, however, [17] did not find much difference in incidence between children of West Indian origin and that among whites.

With the exception of the data from Addis Ababa (Ethiopia), incidence rates of ALL in populations of black (African) origin are consistently lower than in North America and Europe (Figure 8.1.1a and [12]), and, for the most part, in US blacks. The age-specific incidence histograms (Figure 8.1.1b) suggest that, except in whites from Republic of South Africa (RSA) and Reunion, there is no peak in incidence in the 0–4 age group, consistent with the notion that pre-B ALL is linked with higher levels of socio-economic development. In Ibadan (Nigeria), Williams (1985) observed that a higher percentage of childhood cases of ALL were of higher socioeconomic status (27%), compared with cases of acute myeloid leukaemia (AML) (6%) or Burkitt lymphoma (3.3%). There was a higher incidence of ALL in coloured children living in Cape Town, compared with those living in rural areas of the Western Cape in RSA [19]; however, the difference may relate to access to medical diagnosis, as much as true differences in risk.

There are few long-term trend data from Africa to examine possible changes in incidence in childhood; however, examination of data from Harare (1990–2012) does not suggest any increase in rates of lymphoid leukaemia.

Apart from ALL, most childhood leukaemias are acute myeloid leukaemia (AML). AML is a leukaemia of myeloid precursor cells that can differentiated into a range of morphological subtypes (some of which are also clinically quite distinct with respect to their treatment and response to it).

In Europe and North America, the ASR is typically in the range 4–9 per million (cum rate 60–130) [4]; the rate in Europe was 65 (ASR) and 90 (cumulative) per million in 1993–1997 [12]. In the USA, the incidence of AML, unlike that for ALL, was similar for white and black children for all age groups [16]. The incidence of AML varies with age, but with a different pattern than that for ALL. In the USA, AML rates were highest in the first 2 years of life, but subsequently decrease with a nadir at approximately 9 years of age, followed by slowly increasing rates during the adolescent years [16]. In Europe, the rates are highest in the 0–4 age group, and lowest at 5–9 [12].

Incidence rates of AML in the series in this volume are based on small numbers, but in general, the rates are rather lower than in the United States or Europe.

Reports from Africa have sometimes suggested that AML is relatively common, but this is most likely because of the deficit of cases of ALL, rather than a particularly high incidence of AML. In RSA, the rates in blacks and whites were the same, and in an earlier report [20], the incidence in black children was no higher than in whites. Chloromas (solid leukaemic masses) have been reported to complicate up to 25% of cases of acute myeloid leukaemia [21].

### 8.2. Lymphomas

**Table 8.2.**
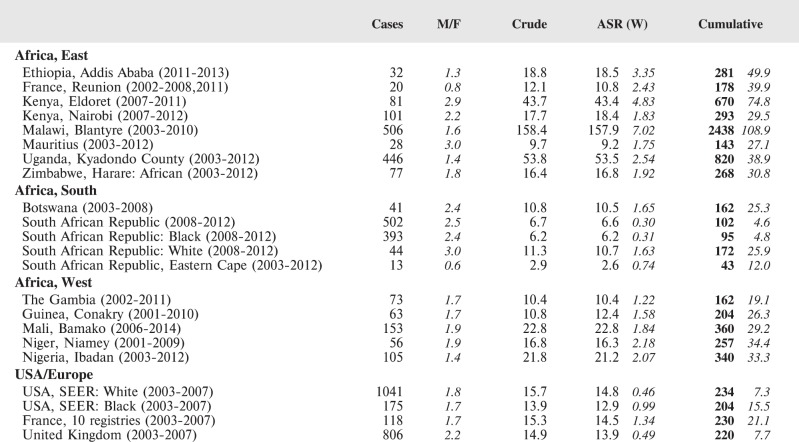
Crude, age-standardised (world), and cumulative (0–14) incidence rates (per million) and standard errors – lymphomas.

**Figure 8.2.**
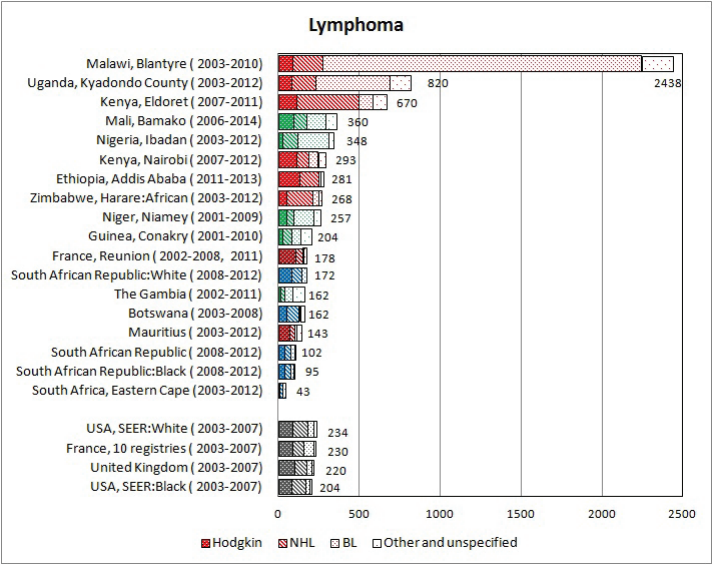
Cumulative (0–14) incidence rate per million – lymphomas: Hodgkin, NHL, BL and others.

#### 8.2.1 Hodgkin lymphoma

**Table 8.2.1a.**
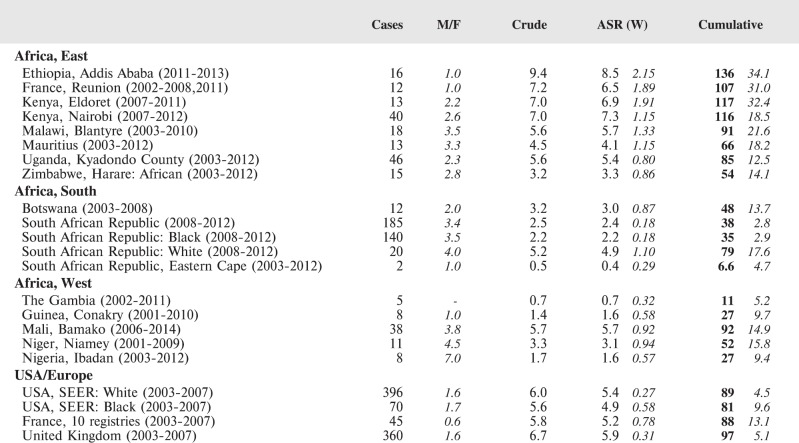
Crude, age-standardised (world), and cumulative (0–14) incidence rate (per million) and standard errors – Hodgkinlymphoma.

**Figure 8.2.1a.**
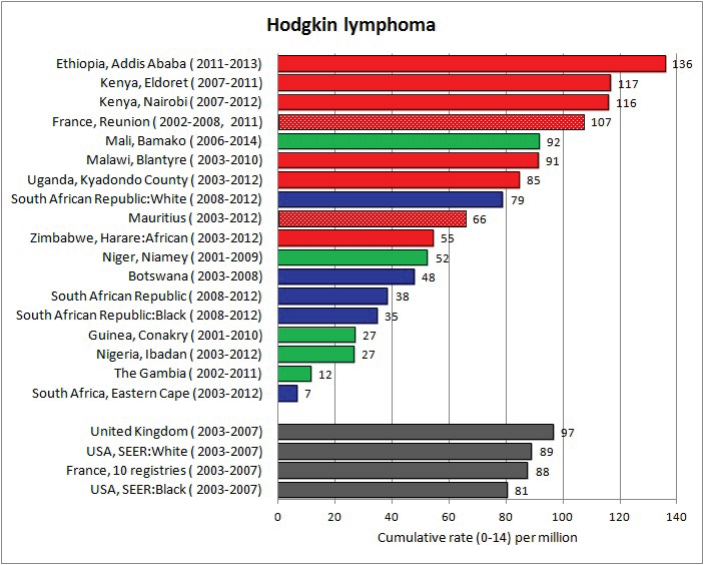
Cumulative (0–14) incidence rate – Hodgkin lymphoma.

**Figure 8.2.1b.**
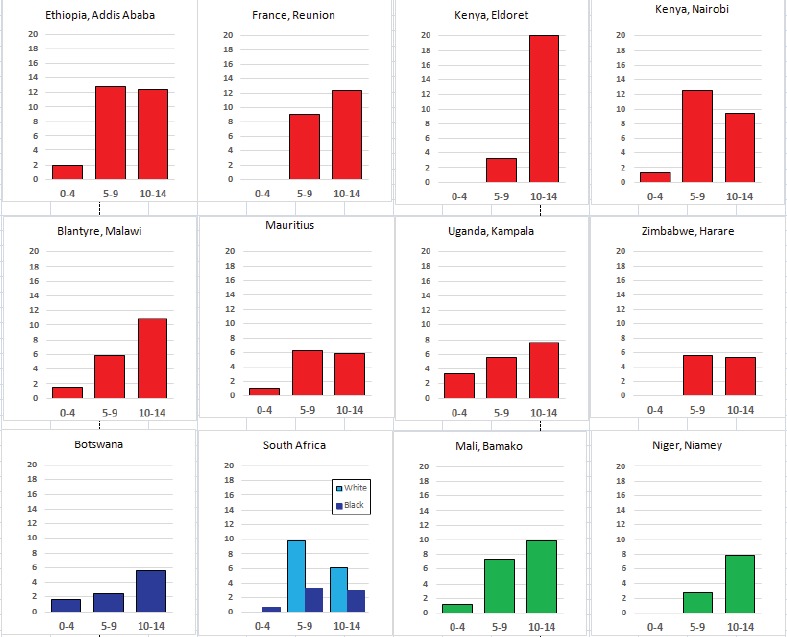
Age-specific histograms – Hodgkin lymphoma.

Stiller and Parkin (1990) [22] observed that, although it was not possible to estimate incidence of childhood Hodgkin disease in Africa, most published series of childhood cancers contained ‘considerable numbers’ of cases. Table 8.2.1 and the bar chart Figure 8.2.1a suggest that the incidence in many centres in sub-Saharan Africa is indeed similar to that in populations of the developed countries of Europe and North America.

In white populations of Europe and North America, incidence rates rise progressively throughout childhood (HL is very uncommon under age 5) to reach a ‘young adult’ peak at about age 25 years [16]. Hodgkin lymphoma is more common among males (M/F = 1.3 in the USA, 1.7 in Europe), with the differential greatest for children younger than 5 years of age [16, 23]. In the USA, rates are slightly higher in whites than blacks (Figure 8.2.1a), however, the incidence was very similar for black and white children younger than 10 years of age [16]. The majority of cases in childhood are of the nodular sclerosis subtype (~70% in the USA, 60% in Europe), becoming relatively more common in the older age groups. Mixed cellularity cases are rarer (16% in the USA, ~30% in Europe), but it was more common in Eastern Europe (40% of cases), associated with a small peak of incidence at ages 5-9 in boys [23].

Correa and O’Conor (1971) were the first to observe that the age-specific incidence pattern in developing countries is rather different, with an initial peak in childhood (especially in boys), no young adult peak, and then a rise in incidence to old age.

It is thought that HL may represent two distinct diseases with different aetiology: an EBV-related form of the disease, more prevalent in developing countries, in young children, in boys and mainly presenting as mixed cellularity HD, and a form unrelated to EBV, more prevalent in industrialised countries, in adolescents and young adults with slight female predominance and presenting as nodular sclerosis.

Figure 8.2.1b shows age-specific incidence in age groups 0–4, 5–9, and 10–14 years in African registries with more than 10 cases. Small numbers make interpretation difficult, but rates are always low before age 5; thereafter, there is some variation, with some populations showing higher rates at 5–9 than at 10–14. Allowing for small numbers, it appears that the sex ratio is generally higher than in Europe or the USA.

Case series from Africa confirm the relatively young age of patients, with a relatively high percentage of cases occur in children (Parkin et al, 2002). Case series from Africa with adequate detail on histological subtype are rather few [24, 25, 26]. African children have an excess of mixed cellularity (MC) subtype and a marked deficit of nodular sclerosing (NS) cases (nodular sclerosis subtypes being relatively more common in white children in RSA [25], while lymphocyte depleted (LD) cases may comprise up to 30% in some series.

Positivity for Epstein–Barr virus (EBV) in malignant cells is more common in childhood Hodgkin disease than in adults, in the MC subtype and in cases from developing countries [27]. There is a higher prevalence of EBV in Hodgkin disease cases from Kenya than in European countries, with almost all paediatric cases from Kenya being EBV-positive [28, 29].

#### 8.2.2 Burkitt lymphoma

**Table 8.2.2.**
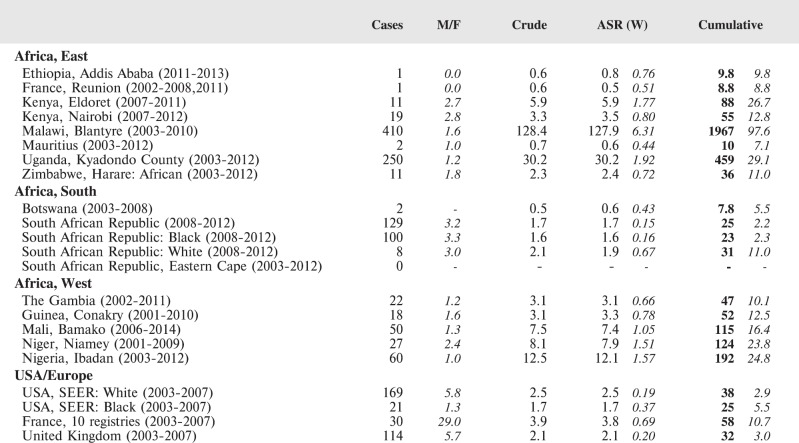
Crude, age-standardised (world) and cumulative (0–14) incidence rates (per million) and standard errors – Burkitt lymphoma.

**Figure 8.2.2a.**
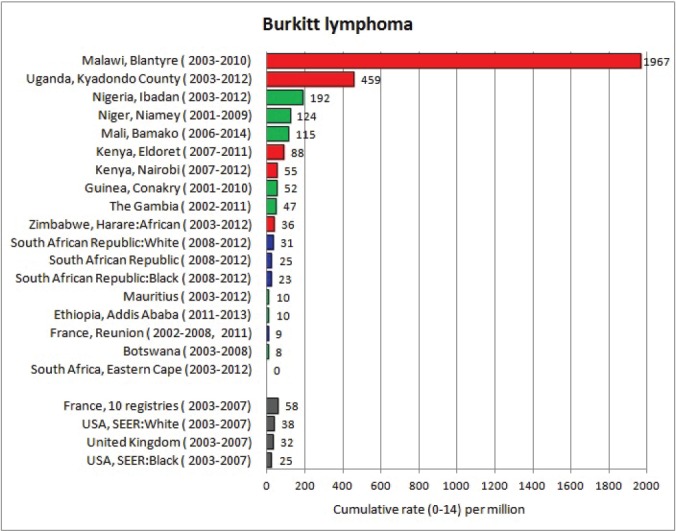
Cumulative (0–14) incidence rate – Burkitt lymphoma.

**Figure 8.2.2b.**
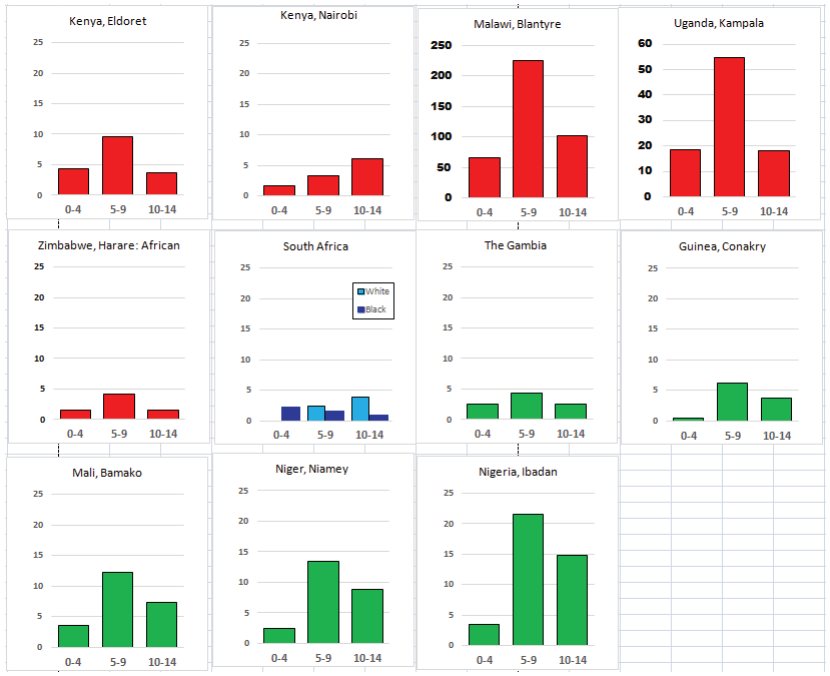
Age-specific histograms – Burkitt lymphoma.

Burkitt lymphoma (BL), an aggressive B-cell lymphoma first recognised as a tumour of African children, occurs throughout the world, but has a markedly different incidence in different world regions, and even within regions. By far the highest incidence rates of BL are found in tropical African countries, and the tumour in these regions is consequently referred to as ‘endemic BL’. Other African countries have much lower incidence rates, which are similar to those of very highly developed countries. BL in these regions is consequently referred to as ‘sporadic BL’. Immunodeficiency-associated Burkitt lymphoma is primarily associated with HIV infection. These ‘variants’ of BL differ in their clinical characteristics, most obviously with respect to the frequency of jaw, orbital and other facial tumours, but there is sufficient overlap in the anatomical sites of disease, histopathology, immunophenotype, as well as response to the same treatment (although randomised trials have not been done), to justify the consideration of eBL and sBL as variants of the same disease. Understanding the relationship between tumours, however, requires careful epidemiological studies, which are difficult to perform in Africa at the present time because of lack of resources, particularly good pathology laboratories, and in the absence of widely accepted definitions of eBL and sBL may confuse rather than clarify. Molecular characterisation, however, of BL in Africa and BL in the USA and Europe, and the discovery and deciphering of the biology of Epstein–Barr virus (EBV), which is associated with the vast majority of BLs in the world, and essentially all BLs in tropical zones has led to a much clearer understanding of the similarities and differences of BL in its classical and variant forms, and the role of EBV, in EBV-positive and EBV-negative BL. Most recently, the development of strong evidence that malaria (*Plasmodium falciparum*) predisposes to BL, after many years of studying possible interactions between these two organisms in BL pathogenesis without being able to demonstrate definitive, collaborative interactions has finally led to an understanding of the overlapping pathways of tumorigenesis between these two organisms in the context of BL, as well as a clearer idea of the role played by EBV in the pathogenesis of BL in subtropical and even temperate regions.

#### Incidence and geographical occurrence

In the series reported in this volume, the highest rates are observed in the population of Blantyre (Malawi), with a remarkable age standardised rate of 128 per million corresponding to a cumulative risk of a child developing BL of about 2 per 1000. The next highest rate in observed in Kampala (Uganda) – cumulative rate of 450 per million, ASR 30 per million. Rates about one quarter this level are observed in several West African centres, but in much of southern Africa, the incidence is little different from that in Europe and North America (Figure 8.2.2a).

The geography of BL in Africa has been the subject of many studies, beginning with the early work of Burkitt, which relied upon collecting information on the relative frequency of cancers in clinical series from hospitals in Africa. He delimited a zone 15° north and south of the equator (with a prolongation southward into Mozambique to the east) as the high-incidence area although even within this area, BL was noted to be infrequent in high-altitude regions, such as Rwanda and Burundi, the Kenya Highlands and the plateaux of Zambia and Zimbabwe [30]. These (and other) observations led to the linkage to the geography of holo-endemic malaria [31, 32].

In the early clinical series from Uganda [33], the peak incidence was 4–7 years, with the jaw being the most frequently involved site and the commonest presenting feature, and patients with jaw tumours being slightly younger than those without. Patients less than one year do not occur, or are extremely rare; and Burkitt found that less than 1% are under the age of 2 years and less than 3% under 3 years. Cases from Ibadan had a similar age distribution – in a series of 67 cases, the peak age was 7 years [34]. The male:female ratio is close to 2:1 in most published series prior to the era of HIV. However, in children with jaw tumours, it is closer to 3:1 and in children without jaw tumours approximately 1:1.

The series from higher incidence areas in this volume show a peak of incidence at ages 5–9 (Figure 8.2.2b), with sex ratios (M:F) in the range of 1:3.

#### Aetiology

IARC (2012) [34] considers that there is *sufficient evidence* in humans for the carcinogenicity of EBV with respect to Burkitt lymphoma. It has been reported that EBV is detected in the tumour tissue of almost 100% of the cases of endemic Burkitt lymphoma, this proportion is less in cases of sporadic and immunodeficiency-associated Burkitt lymphoma [36, 37].

With regard to endemic Burkitt lymphoma, a cohort study in north-west Uganda showed that high EBV VCA antibody titres were regularly detected in children as early as 4 years before tumour development, which indicates an early infection and a high viral load [38]. Several case control studies (reviewed in [35]) have also shown a relationship between an increase in the titre of antibodies against EBV-VCA and an increase in risk for endemic Burkitt lymphoma.

Since EBV infection is almost universal [39], it is clear that there must be important cofactors. The most important is undoubtedly malaria, which is holo-endemic in those areas where endemic BL occurs (reviewed in [40]).

The evidence linking risk of BL to malaria infection was originally derived from ecological comparisons and was reviewed by Morrow (1985) [41]. The principal observations are that the incidence of BL correlates within and between countries with the incidence of malaria and with parasitaemia rates that the age at which peak levels of antimalarial antibodies are acquired (5–8 years) corresponds to the age of peak incidence of BL (and there is an inverse relationship between the age at onset of BL and the intensity of infection with *Plasmodium falciparum*), and BL incidence declines in regions where death rates due to malaria have declined.

An intervention study in northern Tanzania showed an apparent decline in the incidence of endemic Burkitt lymphoma following the introduction of a malaria eradication programme[42].

It has been harder to demonstrate an association between the risk of BL and malaria infection at an individual level. However, several case–control studies have demonstrated an increasing risk of endemic Burkitt lymphoma in relation to an increase in the titre of antibodies against malaria and also confirmed the synergistic roles of EBV and malaria in the aetiology of the disease [43–46].

IARC (2014) [47] therefore classifies malaria caused by infection with *P. falciparum* in holoendemic areas as probably carcinogenic to humans (group 2A).

HIV is an established risk factor for AIDS-related Burkitt lymphoma [35], but there is little evidence that the risk of endemic Burkitt lymphoma is changed by HIV infection [48, 49]. Despite a high prevalence of HIV in malaria-endemic areas, no epidemic increase in endemic Burkitt lymphoma has been described in Africa.

#### Molecular pathology

At the molecular level, the most consistent factor implicated in the pathogenesis of BL is the translocation of the *MYC* gene (8q24) next to one of the immunoglobulin gene (IG) loci (14q32, 2p12 and 22q11). As a result, control of normal MYC expression is lost, leading to the constitutive expression of the MYC protein. MYC is a global regulator of transcription that affects thousands of genes involved in cell cycle control, cell proliferation, and metabolism, regulation of RNA processing, microRNA expression, signal transduction, cell–cell interaction, immune function, and apoptosis. MYC-driven tumours usually acquire additional genetic mutations or epigenetic modifications that promote cell survival and shift the balance between proliferation and apoptosis towards proliferation. In this regard, the low NF-kB profile in MYC-driven lymphomas could reflect the normal differentiation programme of GC-derived B cells and provides a selective advantage to MYC-transformed lymphomas. However, two pivotal studies [50, 51] revealed the existence of about 10% of BL cases with gene expression profiling (GEP) comparable with the classical ones, but with the absence of the typical translocation. These cases were negative for MYC translocation by FISH analysis, using both split and fusion probes for t(8;14), as well as IgH and IgL split probes. It was demonstrated that alternative mechanisms may be responsible of MYC over-expression in the absence of a detectable translocation; among these, microRNAs (miRNAs) deregulation, epigenetic mechanisms and NMYC over-expression [52]. A gene expression analysis of BL subtypes by Piccaluga et al. (2011) [53] has shown that endemic BL (eBL) have a GEP and a miRNAs expression profile unique and different from those of sporadic BL. In fact, because eBL is known to have an onset in a contest of chronic antigen stimulation (EBV, malaria, and arboviral infections), it is not surprising that its molecular signature includes many genes involved in immune response regulation.

Essentially all African children are infected by EBV during their first year of life so that essentially all African BLs are EBV positive. EBV first infects naıve B cells and activates a growth programme in these cells (also termed latency III), which is characterised by expression of nine latent viral genes. These cells will be recognised and targeted by T-cell-mediated immune response, but a fraction of these will instead enter the germinal centre, where they express only three latent viral genes (default program or latency II). In proliferating germinal centre (GC) B cells, the process of somatic hypermutation, which modifies the DNA of the variable region of immunoglobulin genes, is activated, and GC B cells finally differentiate into memory B cells or plasma cells. The virus thus gains access to the memory B-cell compartment, its main reservoir during persistence, where no latent viral genes are expressed. An exception occurs when the latently infected memory B cells divide, in which case they express the EBNA-1 protein (latency I, characterising the BL samples), thereby allowing viral DNA to replicate. However, recent evidence has challenged the view that only the latency phase of EBV infection is significant for the development of EBV-associated malignancies, proposing that lytic EBV replication may be of pathogenic relevance also in the context of an active immune response. In fact, a recent study [53] on EBV-infected endemic BLs identified a non-canonical latency programme of the virus characterised by a large number of cases expressing LMP-1/-2A/- 2B along with lytic reactivation. Lytically infected B cells secrete factors that may promote tumorigenesis, including growth and angiogenesis factors, immunosuppressive cytokines and genes involved in metabolic reprogramming. Interestingly, EBV-positive BL are characterised by altered lipid metabolism [55]. It was observed heterogeneity in lytic/latent expression programmes not only between the different samples but also within the same specimen, on a cell-to-cell basis [53]. Intra-patient heterogeneity might be related to the activation of the immune response following the expression of the viral genes. These findings was supported by the data on the mutational landscape of eBL, characterized by a lower number of point mutations in genes previously found altered in sBL [56], including *MYC*, *ID3*, *TCF3*, *DDX3X*, *CCND3*, and *TP53* [53]. A near mutual exclusivity between *TCF3/ID3* mutations and the presence of EBV was detected, indicating that TCF3 pathway is more significantly activated in EBV-negative cases [54]. EBV may impact on host cell homoeostasis also by interfering with cellular miRNAs expression and by encoding its own genes and miRNAs. EBV-encoded miRNAs impact cell proliferation, cell death, and immune escape [57].

Malaria *per se* is a cofactor of BL; in fact, malaria parasites are strong polyclonal stimulators of the B-cell system, thereby increasing the likelihood of chromosomal translocations. Moreover, certain *Plasmodium falciparum* antigens and exposure to a large number of antigens during multiple infections will reactivate the virus from memory B cells, thereby increasing viral load and consequently the number of EBV-infected B-cells *in vivo*. CMV and KSHV presence has been demonstrated in eBL samples [54] contributing to chronic antigenic stimulation leading to an extrinsic activation of the BCR signalling [58].

An intermediate situation may be the presence of EBV in the absence of *P. falciparum* – a situation that applies largely to less developed countries outside tropical regions where the fraction of EBV-infected cells varies in different regions, to a significant degree because of different socioeconomic circumstances which influence the age at which EBV infection occurs.

#### 8.2.3 NHL (except Burkitt)

**Table 8.2.3.**
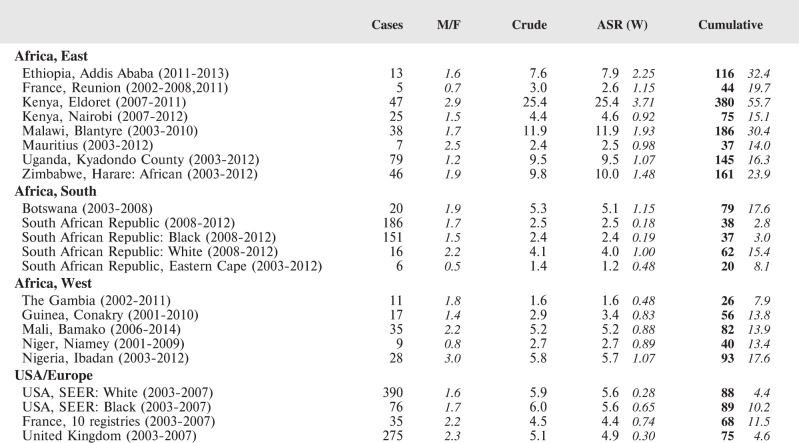
Crude, age-standardised (world) and cumulative (0–14) incidence rates (per million) and standard errors – NHL (except Burkitt).

**Figure 8.2.3a.**
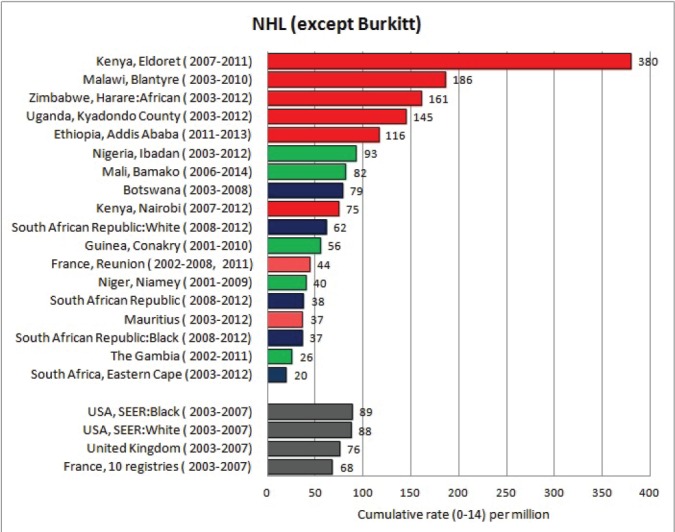
Cumulative (0-14) incidence rate – NHL (except Burkitt).

**Figure 8.2.3b.**
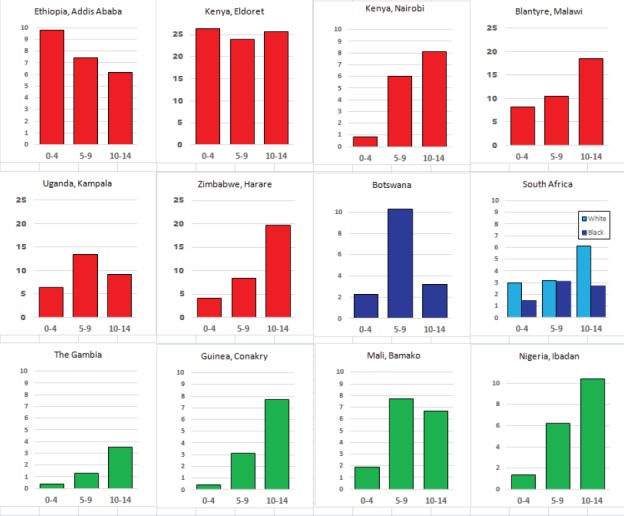
Age-specific histograms – NHL (except Burkitt).

The greatest problem in the study of NHLs has always been that these do not by any means constitute a single diagnostic entity. The very title indicates that it is a diagnosis of exclusion. Many attempts at classification of this group of neoplasms have been made, but the most usual criteria chosen, such as morphological pattern, immunological origin, histological grade and prognosis may have little or no relevance to aetiology, the usual concern of epidemiologists. The most recent WHO classification attempts to incorporate morphological, immunophenotypic and molecular information to produce groupings with more relevance to cause [59].

Unfortunately, most studies, descriptive and analytic, use older terminologies that are impossible to translate into equivalents in more modern schemas. It is generally necessary, therefore, to group together all ‘non-Hodgkin lymphomas’, accepting that this will be grossly unsatisfactory for elucidating meaningful patterns.

Non-Hodgkin lymphoma in childhood is nearly always high-grade. Lymphomas other than BL usually have a total ASR of 5–9 per million, though incidence rates may be a little higher than this in North Africa. In the United States, blacks have a lower rate than whites. Though these geographical patterns are assumed to be related to environmental exposures, the high incidence in young Jewish migrants to Israel from North Africa is retained in their Israeli-born offspring, indicating that genetic susceptibility may also be involved [60].

### 8.3. Brain and spinal neoplasms

**Table 8.3.**
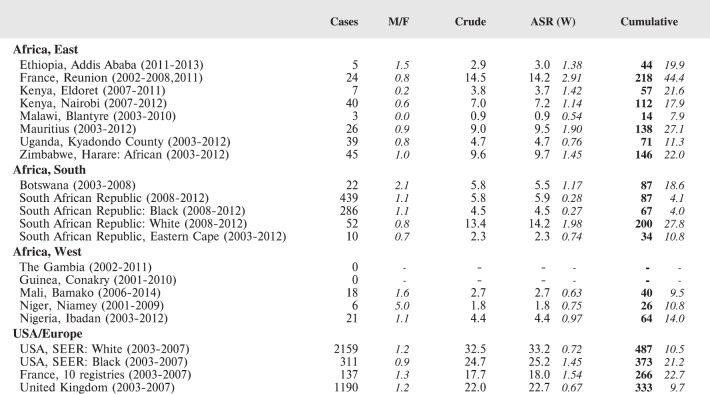
Crude, age-standardised (world) and cumulative (0–14) incidence rates (per million) and standard errors – brainand spinal neoplasms (malignant).

**Figure 8.3a.**
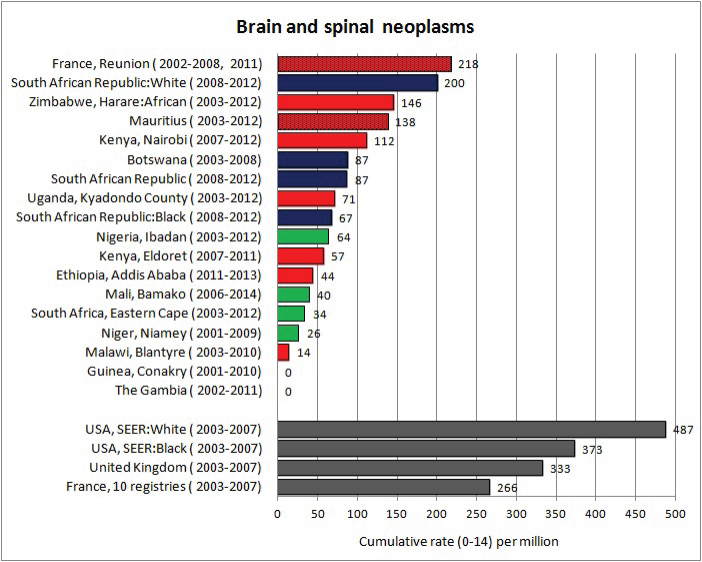
Cumulative (0–14) incidence rate – brain and spinal neoplasms (malignant).

**Figure 8.3b.**
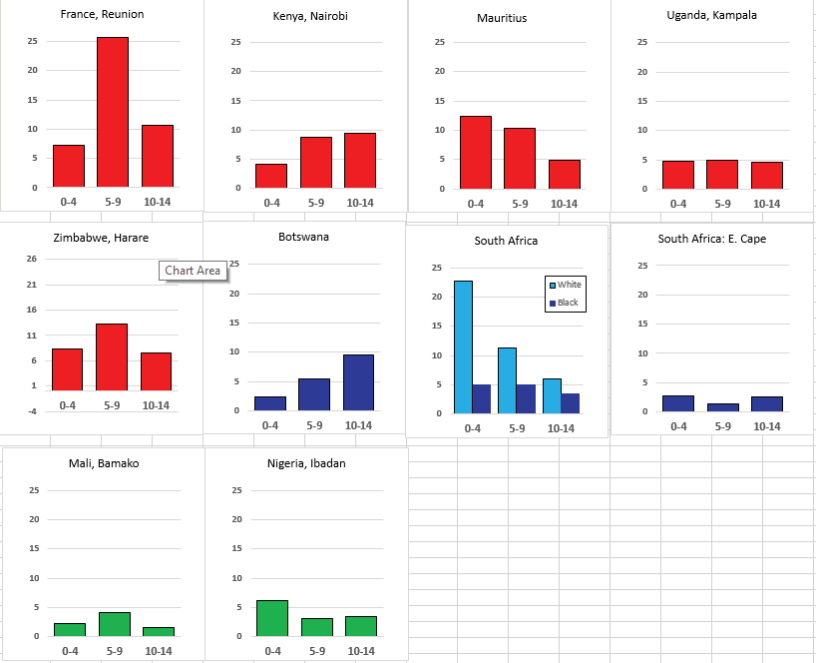
Age-specific histograms – brain and spinal neoplasms (malignant).

**Table 8.3a.** Distribution of the malignant brain and spinal tumours by subtype (registries with 10 or more registered cases).

Registry (period)	a	b	c	d	e	f	
	Ependymomas and choroid plexus tumour	Astrocytomas	Embryonal tumours	Other gliomas	Other specified tumours	Unspecd	ALL
France, Reunion (2002–2008, 2011)	5	3	7	8	1	0	24
Kenya, Nairobi (2007–2012)	0	9	12	7	1	11	40
Mauritius (2003–2012)	0	10	9	4	0	3	26
Uganda, Kyadondo County (2003–2012)	2	4	2	2	0	29	39
Zimbabwe, Harare: African (2003–2012)	1	5	8	5	1	25	45
Botswana (2003–2008)	0	8	7	3	0	4	22
South African Republic (2008–2012)	56	110	108	94	27	44	439
S.A. Republic: Black (2008–2012)	31	68	70	60	21	36	286
S.A Republic: White (2008–2012)	8	17	12	12	1	2	52
S.A Republic, Eastern Cape (2003–2012)	1	2	1	1	0	5	10
Mali, Bamako (2006–2014)	0	3	1	2	1	11	18
Nigeria, Ibadan (2003–2012)	4	5	8	3	1	0	21
	108	244	245	201	54	170	1022
	11%	24%	24%	20%	5%	17%	100%

In developed countries, brain and spinal tumours typically account for 20–25% of all childhood cancer, with a cumulative incidence of 300–500 per million. The most common subgroup is astrocytoma, which covers a wide spectrum of histological types from the relatively benign juvenile astrocytoma (including optic nerve glioma) to the aggressive anaplastic astrocytoma and glioblastoma multiforme. The second most common category comprises primitive neuroectodermal tumours, most of which are cerebellar medulloblastoma. Ependymomas (including choroid plexus tumours) are relatively rare and, like astrocytomas, this category encompasses a wide range of degrees of malignancy.

In developing countries, brain and spinal tumours are usually outnumbered not only by leukaemias but also by lymphomas and recorded incidence is lower than in developed countries. In Africa, rates are very variable, but in general very low, rarely exceeding 150 per million (Table 8.3). There is almost certainly considerable under-ascertainment, because of deficiencies in diagnostic facilities.

Table 8.3a shows the distribution of the registered brain and spinal tumours by subtype. Note the relatively large percentage (17%) for which histological subtype was unknown; they comprise the majority of cases in several registries. In the series in which all cases are histologically verified (RSA national registry, Mauritius) there are approximately equal numbers of astrocytomas and embryonal tumours (medulloblastomas/medulloepitheliomsas and primitive neuroectodermal tumours).

There is some evidence from comparisons of incidence rates within the same country that risk may vary between ethnic groups. Black children in the United States have a lower ASR than whites, while children of West Indian descent in the United Kingdom have a low frequency of brain tumours [17]. The lower recorded incidence in black children than in white in RSA probably is largely related to differential biopsy rates.

### 8.4. Neuroblastoma

**Table 8.4.**
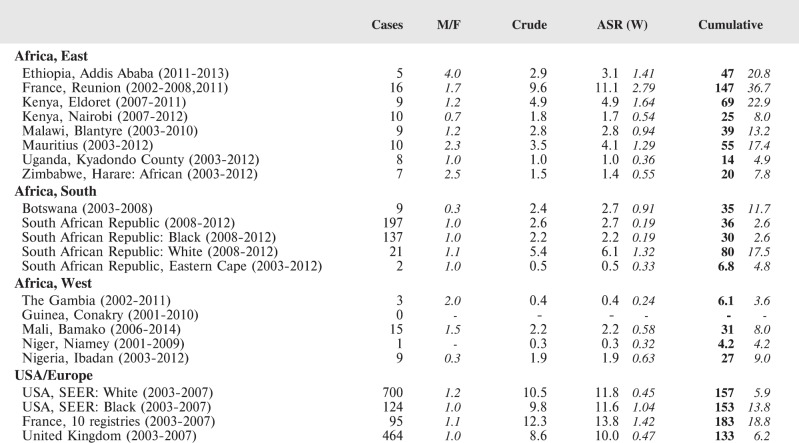
Crude, age-standardised (world) and cumulative (0–14) incidence rates (per million) and standard errors – neuroblastoma.

**Figure 8.4a.**
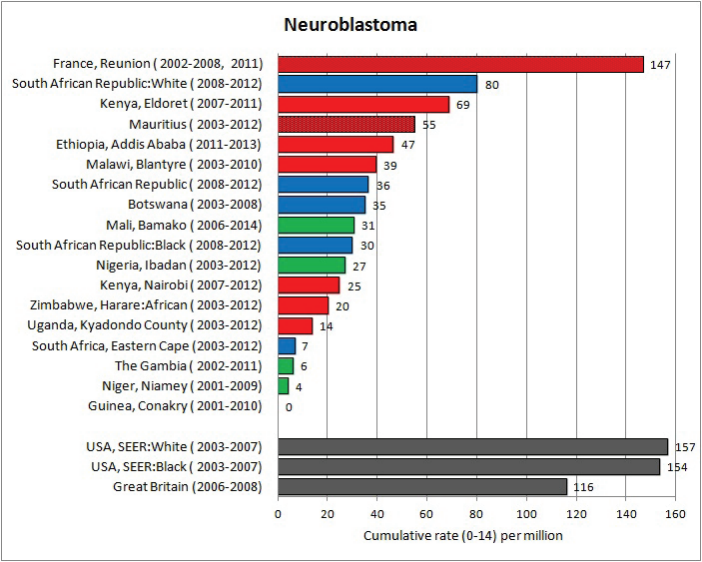
Cumulative (0–14) incidence rate – neuroblastoma.

**Figure 8.4b.**
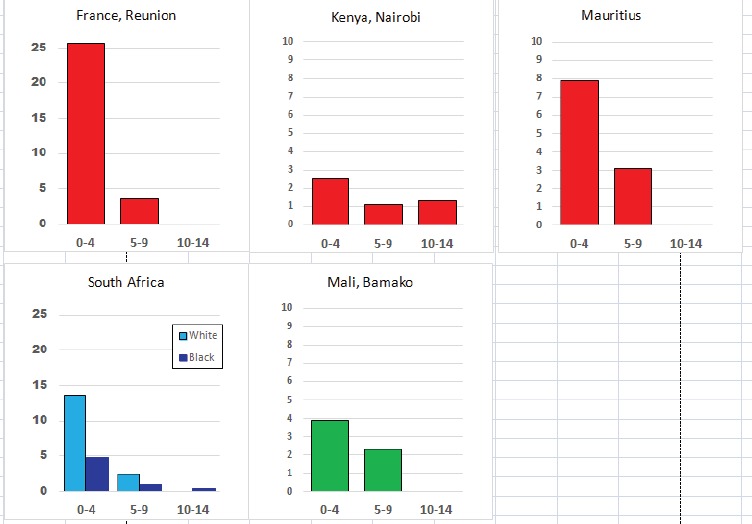
Age-specific histograms – neuroblastoma.

In the predominantly white populations of Europe, North America and Oceania, the cumulative rate is generally in the range 100-180 per million, and 6–10% of all childhood cancers are neuroblastomas [4]. Rates are highest (25–50 per million) in the first year of life, when this is very often the commonest of all cancers. In the United States, blacks had a lower incidence than whites in infancy, but similar rates thereafter, whereas in the United Kingdom, there is no sign of variation between ethnic groups [17].

Although the highest recorded incidence rates for neuroblastoma have long been in industrialised countries with a high material standard of living, studies in Denmark and the United States found that it was more common among less affluent groups [18]. However, the low incidence recorded in (black) African populations does not fit with this observation, and it seems likely that at least some of the deficit in developing countries compared with industrialised countries is due to underdiagnosis (Stiller & Parkin, 1992). For example, the rates in black children in South Africa are considerably lower than rates in whites (Table 8.4), and this most likely reflects different rates of investigation (almost all have been diagnosed by histological examination (Table 5.4, Chapter 5)).

The incidence rates recorded in black African populations in West and East Africa are generally much lower than in Europe and North America (Table 8.4). Early clinical series from tropical Africa had reported a low relative frequency of neuroblastoma among childhood solid tumours [61]. Miller (1977) [15] noted that neuroblastoma cases were infrequent in series from East Africa (Kenya, Tanzania, Malawi, Zambia); in the data from Uganda, and from West Africa (Dakar and Ibadan), the relative frequency was higher, but the ratio of neuroblastoma cases to Wilms tumour cases was less than half of that in blacks in the United States. In a series of 1522 histologically diagnosed solid tumours of children in Kenya, Kung’u (1984) [62] observed only two cases of neuroblastoma (although there were many ‘unspecified small round-cell sarcomas’ of young children in the series) and more recent series [63, 64] have reported similarly low relative frequencies (0.5% and 0%, respectively). Miller (1989) [65] drew upon relative frequency data cited above [15] and data in International Incidence of Childhood Cancer [66] to draw attention to the very low frequency ( < 1% of childhood cancers) in Kenya, Malawi, Tanzania, Uganda, Zaire, and Zambia. The low rates of neuroblastoma in Ibadan, Nigeria, reported in Table 6 echo the rather modest frequency of this cancer in series for the same centre in 1960–1972 (2.6%) [67] and 1960–84 (4.3%) [65]. A recent survey of 10 paediatric oncology centres by members of the Groupe Franco–Africain d’Oncologie Pédiatrique (GFAOP) attempted to assess the relative frequency of neuroblastoma among cases seen in children’s cancer services [68]. In the sub-Saharan centres, neuroblastoma amounted to between 3% and 5% of all paediatric cancers seen (excepting Madagascar, where the frequency was 7.5%); the percentage was between 7% and 30% in the Northern African centres. Most patients presented late, with eight centres reporting between 50% and 80% of cases with advanced stage at presentation.

A disease of very early childhood, neuroblastoma is deemed to originate in events occurring during the intrauterine life. Familial/genetic predisposition and environmental factors have been postulated to play a role in the aetiology [69]. Among the postulated environmental risk factors (which may influence the intrauterine development of the foetus) associated with a higher incidence of neuroblastoma, only folate deficiency [70] and diabetes mellitus [71] could possibly play any significant role on the African continent, but so far there are no published studies on the matter.

### 8.5. Retinoblastoma

**Table 8.5.**
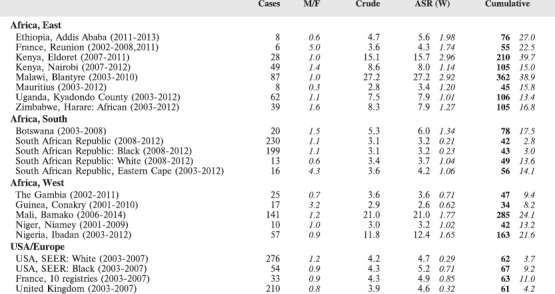
Crude, age-standardised (world) and cumulative (0–14) incidence rates (per million) and standard errors – retinoblastoma.

**Figure 8.5a.**
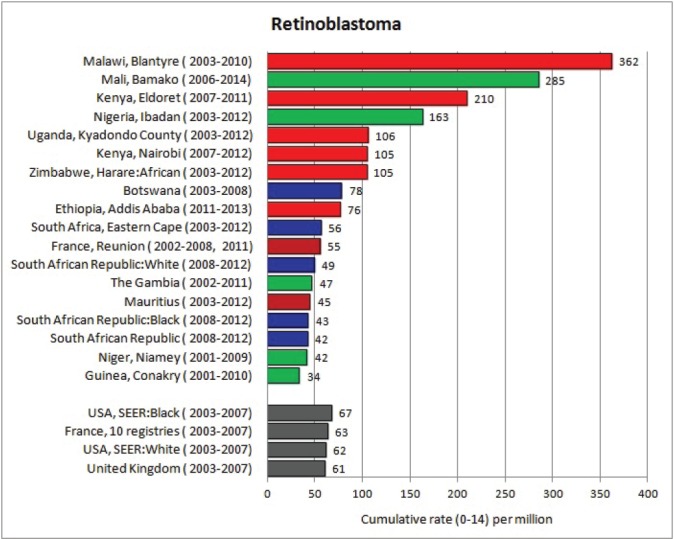
Cumulative (0–14) incidence rate – retinoblastoma.

**Figure 8.5b.**
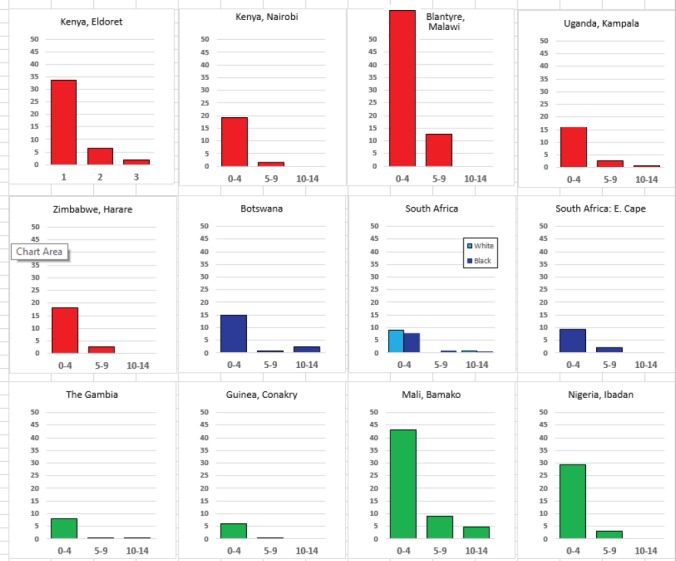
Age-specific histograms – retinoblastoma.

Retinoblastoma is an embryonic neoplasm of retinal origin. Mutations in both alleles of the RB1 gene in a retinal progenitor cell through diverse mechanisms including genetic and epigenetic modifications, is the crucial event in initiation of tumorigenesis. In most patients with sporadic unilateral retinoblastoma, both RB1 gene mutations occur in somatic cells and are not passed over to offspring (nonhereditary retinoblastoma). Almost all patients with sporadic bilateral and virtually all patients with familial retinoblastoma are heterozygous for RB1 gene mutations that cause predisposition to the disease (hereditary retinoblastoma). In families, predisposition to the disease is transmitted as an autosomal dominant trait (familial retinoblastoma).

In most developed countries, the cumulative incidence of retinoblastoma is 50–80 per million and accounts for 2.5–4% of all childhood cancers [4]. It is predominantly a tumour of early childhood, though the proportion of cases occurring among infants aged under one year is rather less than for neuroblastoma. In the United States, incidence among blacks (67 per million) is somewhat higher than among whites (62 per million) (Table 8.5).

The data from Africa (Table 8.5) suggest that, in many of the populations of sub-Saharan Africa, rates are rather higher than in Europe and the United States, with several countries having rates greater than 100 per million.

High relative frequencies of retinoblastomas have been reported in several clinical or pathology series from Africa (although the latter are often restricted to solid tumours). However, care must be taken when interpreting comparisons of relative frequencies. It is likely that retinoblastoma—a relatively easily diagnosed cancer—will be more often correctly diagnosed in Africa than other deep-seated cancers.

It is generally considered that there is less variation in incidence between populations for bilateral tumours (all of which represent the heritable form of the disease, with a genetic aetiology) than in unilateral cases, most of which are sporadic [72]. In Europe, the percentages of bilateral cases are 21% in France, 29% in Germany, and 37% in England and Wales [4]. In the United States SEER registries, the percentage of bilateral cases (1983–1992) was 29% in whites and 44% in blacks.

The numbers of cases in the individual series in Table 8.5 are either too small or laterality is not recorded, so we cannot analyse this feature. However, there are many published accounts of clinical series of cases of retinoblastoma from hospitals around the continent, and almost always the percentage of bilateral cases is relatively low (less than one third). In a recent nationwide case series from Uganda, for example, 26% of 282 cases were bilateral [73]. A deficit of bilateral tumours may represent a higher relative incidence of sporadic, as opposed to heritable cases, but it could also be a consequence of poor survival from the first tumour, or simply failures of recording.

Most case series also note the rather higher mean age at diagnosis of cases in Africa than in Europe or North America. The mean age of the cases in the registry series in Table 8.5 is shown in Table 8.5a. The older age at diagnosis in African children relates to the lower frequency of bilateral tumours (which have an earlier age of onset than sporadic, unilateral cases) and probably also to the relatively advanced stage at which tumours present in clinical practice in Africa [74]. Late diagnosis is undoubtedly related to the poor prognosis of retinoblastoma cases in Africa and is being addressed through programmes of early detection [75].

**Table 8.5a.** Mean age at diagnosis—retinoblastoma.

Registry	Mean age
Ethiopia, Addis Ababa (2011–2013)	3.1
France, Reunion (2002–2008, 2011)	1.8
Kenya, Eldoret (2007–2011)	3.6
Kenya, Nairobi (2007–2012)	2.2
Malawi, Blantyre (2003–2010)	2.6
Mauritius (2003–2012)	2.8
Uganda, Kyadondo County (2003–2012)	3.0
Zimbabwe, Harare: African (2003–2012)	2.4
Botswana (2003–2008)	2.1
South African Republic (2008–2012)	2.2
South African Republic: Black (2008–2012)	2.2
South African Republic: White (2008–2012)	2.2
South African Republic, E.Cape (2003–2012)	2.6
The Gambia (2002–2011)	3.2
Guinea, Conakry (2001–2010)	2.6
Mali, Bamako (2006–2014)	3.5
Niger, Niamey (2001–2009)	2.9
Nigeria, Ibadan (2003–2012)	3.0
USA, SEER: White (2003–2007)	1.2
USA, SEER: Black (2003–2007)	1.5
France, 10 registries (2003–2007)	1.2
United Kingdom (2003–2007)	1.2

### 8.6. Nephroblastoma

**Table 8.6.**
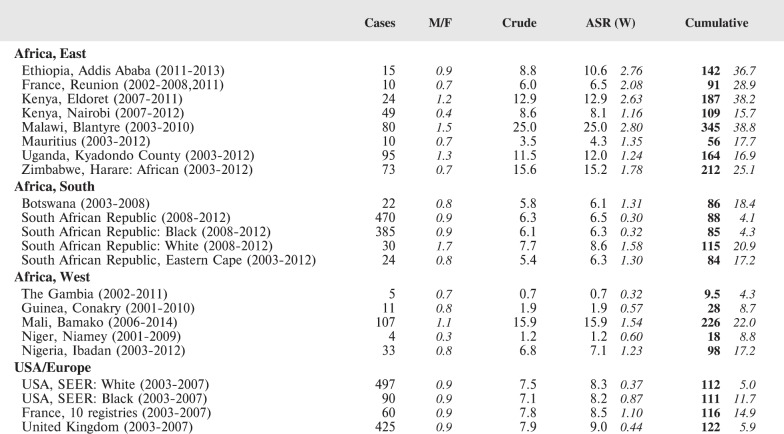
Crude, age-standardised (world) and cumulative (0–14) incidence rates (per million) and standard errors—nephroblastoma.

**Figure 8.6a.**
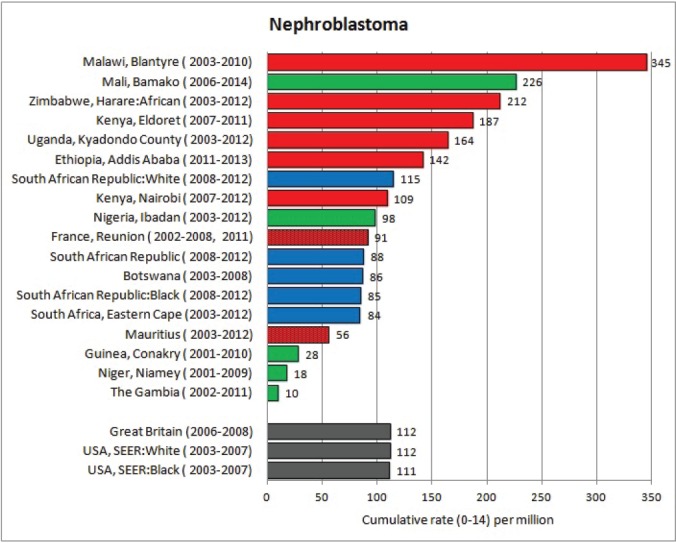
Cumulative (0–14) incidence rate—nephroblastoma.

**Figure 8.6b.**
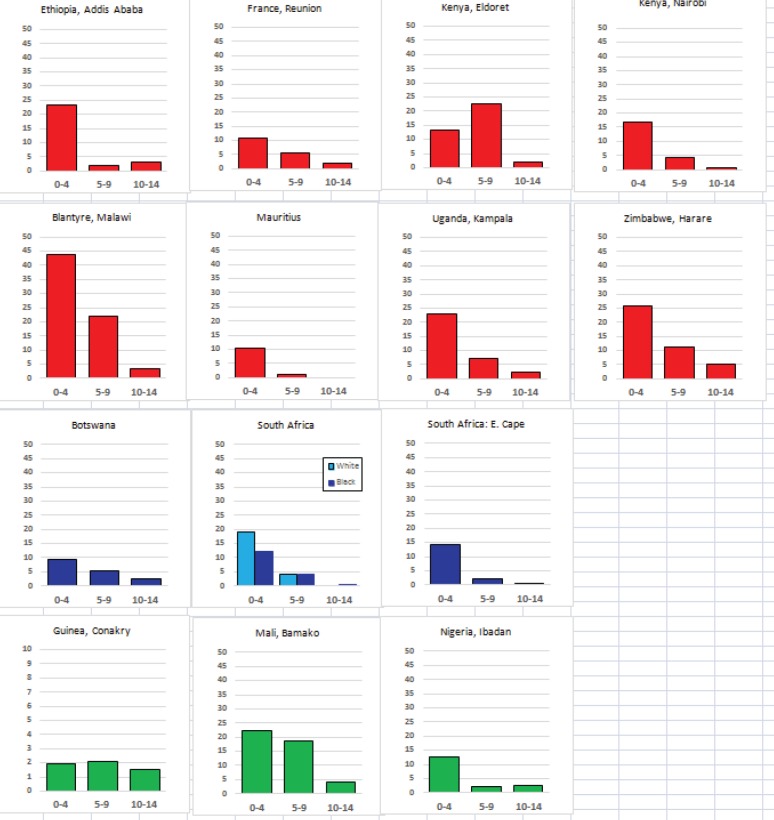
Age-specific histograms—nephroblastoma.

The great majority of childhood renal tumours are Wilms tumours (nephroblastoma). It has long been noted that, worldwide, the highest incidence rates of Wilms tumour are observed among black populations, both in Africa and in series from the United States [22, 76]. However, recent data from the SEER programme (2003–2007) (Table 8.6) do not suggest a higher incidence in black children (cum rate 111.3 per million) than in white children (111.7 per million). In the United Kingdom, children of West Indian descent were reported to have a relatively high frequency of Wilms tumour [17].

Among white populations, Wilms tumour usually has a cumulative incidence of 100–130 per million and accounts for 5–7% of all childhood cancers, though higher rates have been recorded in several Nordic countries, Estonia and New Zealand [4]. The age distributions in white and black populations are similar, with the highest incidence occurring in the second year of life.

The results from the African registries in this volume are varied (Table 8.6). The incidence rates from registries in eastern Africa are high (e.g. in Malawi, Kenya, Uganda, and Zimbabwe), with the lowest rates from Reunion (predominantly white population) and Mauritius (a population predominantly of Asian-Indian origin). Registries in southern Africa report moderate incidence rates (with rates in Rep. of South African whites higher than those in blacks), while those reported from West Africa are very varied (with Bamako (Mail), reporting a rate of 226 (95% CI: 183–269)).

Since incidence of Wilms tumour apparently varies along ethnic rather than geographical lines, it is possible that there is a strong element of genetic predisposition in its aetiology, despite the fact that very few cases can be identified as directly hereditary. No obvious environmental factors have been identified that influence the risk of this cancer [77].

### 8.7. Hepatic tumours

**Table 8.7.**
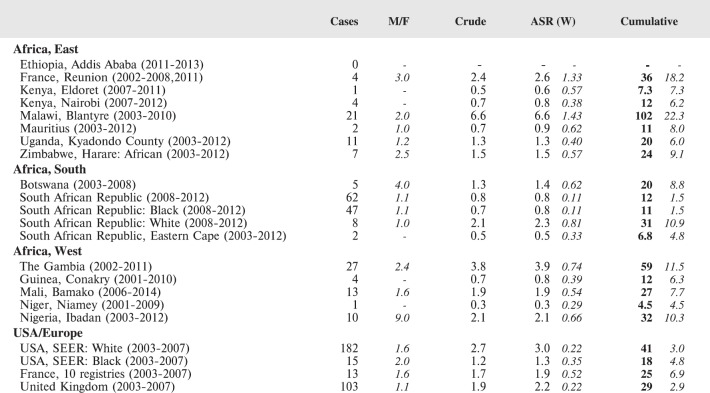
Crude, age-standardised (world) and cumulative (0–14) incidence rates (per million) and standarderrors—hepatic tumours.

**Figure 8.7.**
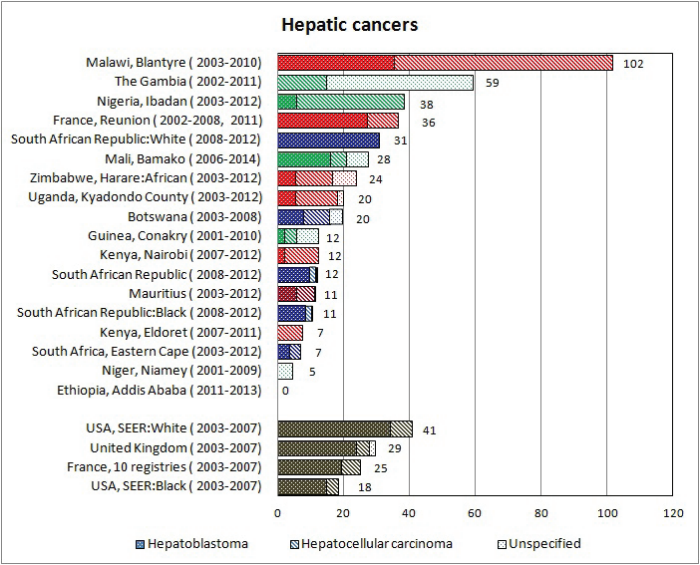
Cumulative (0–14) incidence rate—hepatoblastoma, hepatocellular carcinoma and others.

#### 8.7.1 Hepatoblastoma

**Table 8.7.1.**
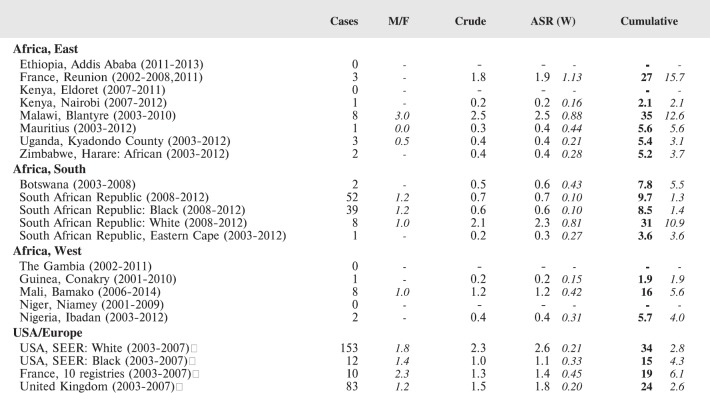
Crude, age-standardised (world) and cumulative (0–14) incidence rates (per million) and standarderrors—hepatoblastoma.

#### 8.7.2 Hepatocellular carcinomas

**Table 8.7.2.**
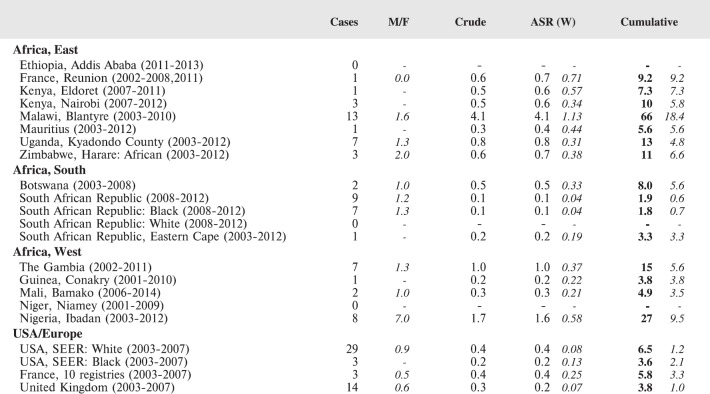
Crude, age-standardised (world) and cumulative (0–14) incidence rates (per million) and standarderrors—hepatocellular carcinomas.

Most malignant liver tumours of childhood are either hepatoblastomas or hepatocellular carcinomas. The other liver tumours in this group are of unspecified cell type. Hepatoblastoma is one of the rarer embryonal tumours. Nearly, all cases are diagnosed in the first few years of life and incidence is highest in infancy. There is apparently very little geographical variation, with a cumulative incidence of 15–30 per million worldwide [4]. In the series from Africa in this volume, the incidence rates in the white population of RSA, and the predominantly white population of Reunion, are similar to those in white populations in Europe and North America (Figure 8.7, Table 8.7.1). Only Blantyre in Malawi has a comparable incidence. Increases in incidence have been reported from the USA, which is thought to be due to an increase in premature births [78]; low birth weight has been shown increase the risk of hepatoblastoma [79].

Hepatocellular carcinoma shows much more geographical variation, though everywhere most cases are in older children (10–14 years). In Europe and North America, it is rare in childhood, occurring with well under half the frequency of hepatoblastoma. It is much more common, however, in regions of the world with high rates of adult liver cancer, including sub-Saharan Africa (especially West Africa). Numbers in the series in this volume are small, but the rates in Malawi, Nigeria (Ibadan) and The Gambia appear to be substantially higher than in European populations (Table 8.7.2).

Most childhood cases of liver cancer in these areas occur in chronic carriers of hepatitis B [80, 81], and chronic HBV infection has been shown to be established in the first 5 years of life [82]. Although aflatoxin exposure is also known to confer a high risk of HCC, and exposure has been shown to occur early in infancy [83], the characteristic mutation in the TP53 tumour suppressor gene at codon 249 (TP53 Ser249 mutation) that is associated with hepatocellular carcinoma tumours was not observed in young children (ages 2–5 years) from Guinea, west Africa, a region of high aflatoxin exposure [84].

The national hepatitis B vaccination programme that began in 1984 in Taiwan has been associated with over 70% reduction in liver cancer rates among children and adolescents [85]. In Rep. of South Africa, Moore et al (2008) [86] noted an apparent decline in the frequency of HCC in a case series from the period 1988–2006, which they ascribed to immunisation.

### 8.8. Bone tumours

**Table 8.8.**
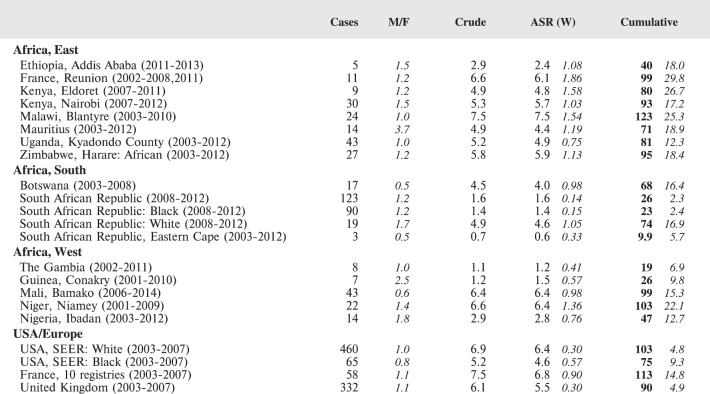
Crude, age-standardised (world) and cumulative (0–14) incidence rates (per million) and standard errors—bone tumours.

**Figure 8.8.**
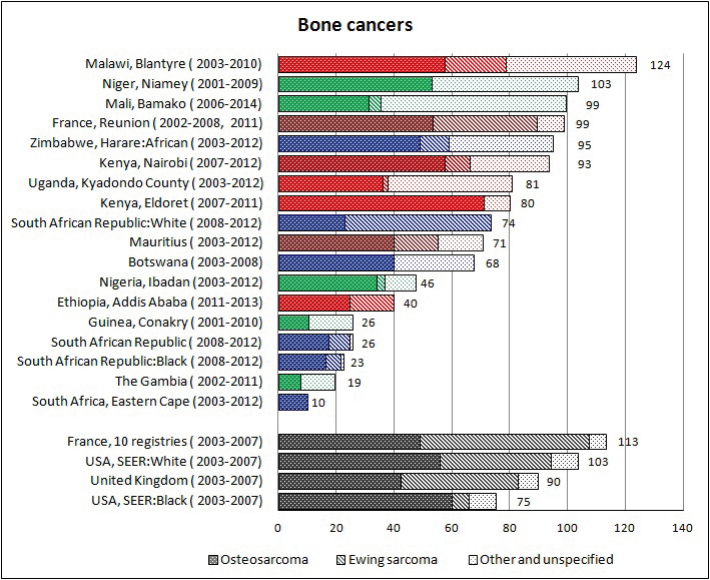
Cumulative (0–14) incidence rate—osteosarcoma, Ewing sarcoma and others.

**Table 8.8.1.**
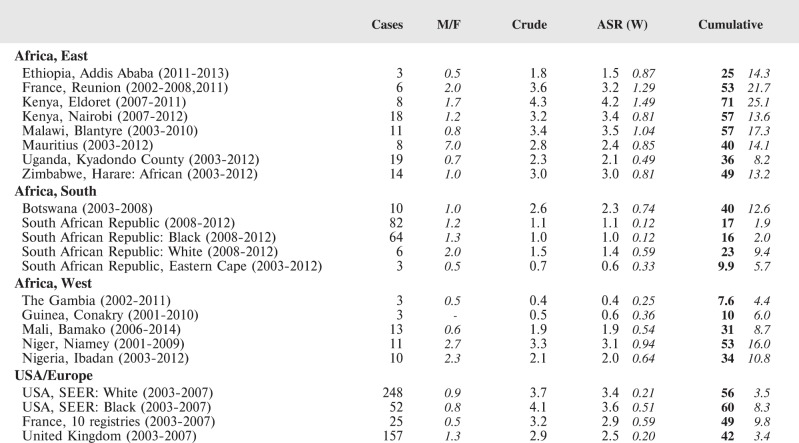
Crude, age-standardised (world) and cumulative (0–14) incidence rates (per million) and standard errors— osteosarcoma.

**Figure 8.8.1a.**
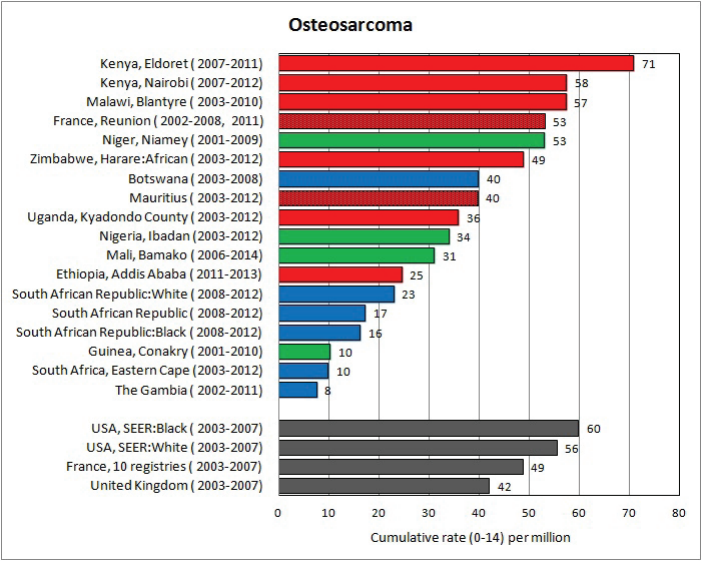
Cumulative (0–14) incidence rate—osteosarcoma.

**Figure 8.8.1b.**
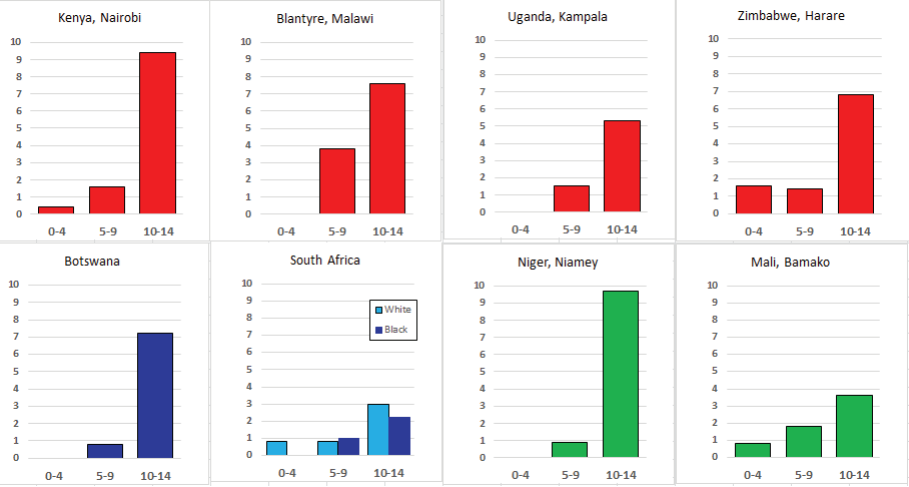
Age-specific histograms—osteosarcoma.

**Table 8.8.2.**
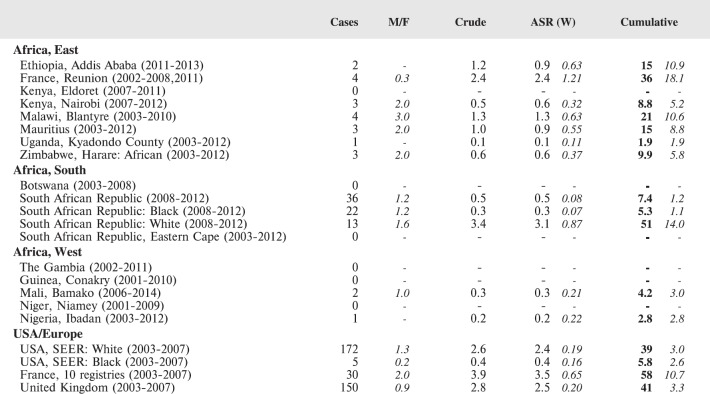
Crude, age-standardised (world) and cumulative (0–14) incidence rates (per million) and standard errors—Ewing sarcoma.

Bone tumours comprise about 5% of all childhood cancers, compared with less than 1% in adults. Almost all are either osteosarcoma or Ewing sarcoma, with fewer than 10% being of other types.

In the series from African registries in this volume, a substantial proportion of cases registered fall into the ‘Other and Unspecified’ category, except in series where a large proportion are histologically verified (South Africa (100%), Reunion (9/11), Addis Ababa (4/5)). Taking this into account, it is quite clear that in black African populations, Ewing’s sarcoma is a rare cancer, and the majority of bone cancers of specified type are osteosarcomas.

The risk of osteosarcoma shows a bimodal distribution throughout life, with the first peak at age 15–19 years, so that incidence within childhood increases with age and more than 70% occurs at age 10–14 years. This is clear from the age-specific histograms in Figure 8.8.1b. In the United States, the incidence was formerly somewhat higher in the black population than among whites [66], but since the 1980s rates have been very similar for the two ethnic groups [4]. A link between the risk of osteosarcoma and bone growth has long been suspected [87]. More than three quarters of tumours arise in the long bones of the legs and there is an excess in girls before age 13 years and in boys thereafter, corresponding to their relative rates of growth.

There is considerably more variation in risk between populations for Ewing sarcoma, with particularly low incidence in black populations. This was first noted in comparisons between rates in black and white children in the United States [88, 89]. The ratio between osteosarcoma and Ewing sarcoma in childhood in populations of African (black) origin is around 10:1, compared with approximately equal numbers in white populations [90]. This suggests that genetic factors are important in predisposition to (or protection against) Ewing sarcoma [91]. Incidence increases with age, though less steeply than for osteosarcoma. Compared with osteosarcoma, Ewing sarcoma arises more frequently in the ribs, pelvis (especially in older children) and skull (in younger children) and correspondingly less frequently in the long bones.

### 8.9. Soft-tissue sarcomas

**Table 8.9.**
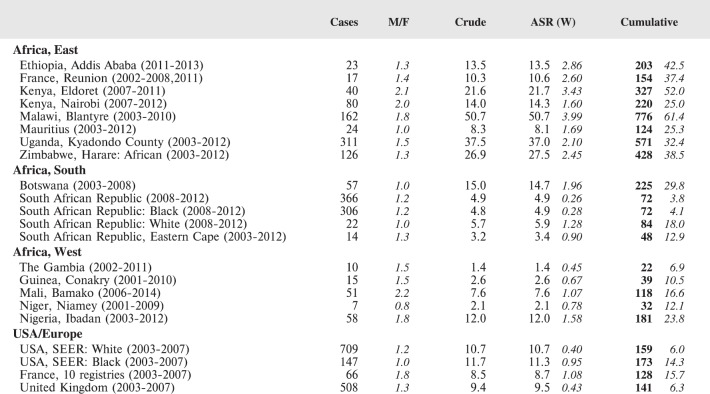
Crude, age-standardised (world) and cumulative (0–14) incidence rates (per million) and standard errors—soft-tissue sarcomas.

**Figure 8.9.**
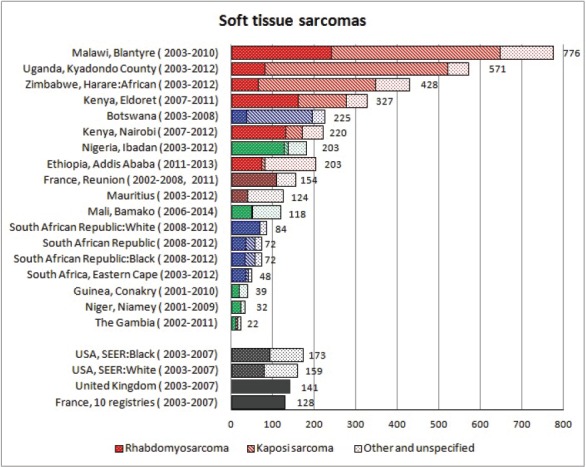
Cumulative (0–14) incidence rate—rhabdomyosarcoma, Kaposi sarcoma and others.

#### 8.9.1 Rhabdomyosarcoma

**Table 8.9.1.**
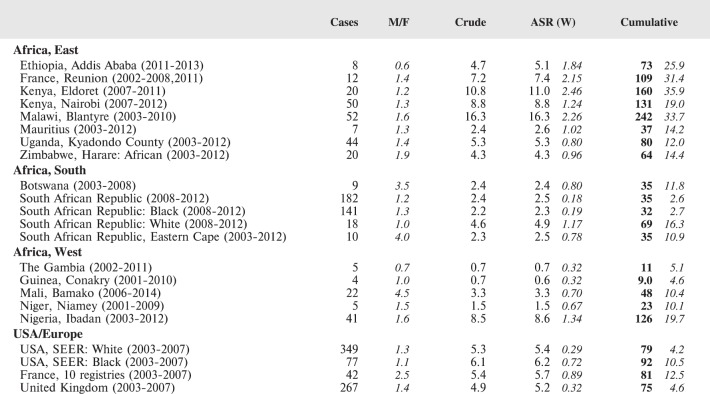
Crude, age-standardised (world) and cumulative (0–14) incidence rates (per million) and standard errors—rhabdomyosarcoma.

**Figure 8.9.1a.**
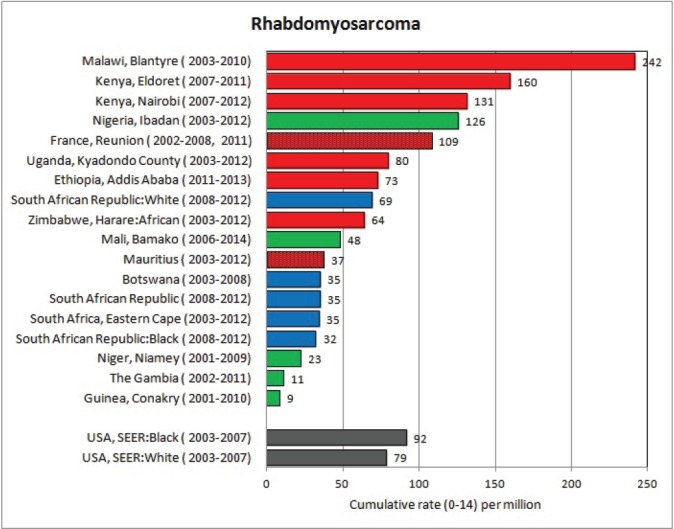
Cumulative (0–14) incidence rate—rhabdomyosarcoma.

**Figure 8.9.1b.**
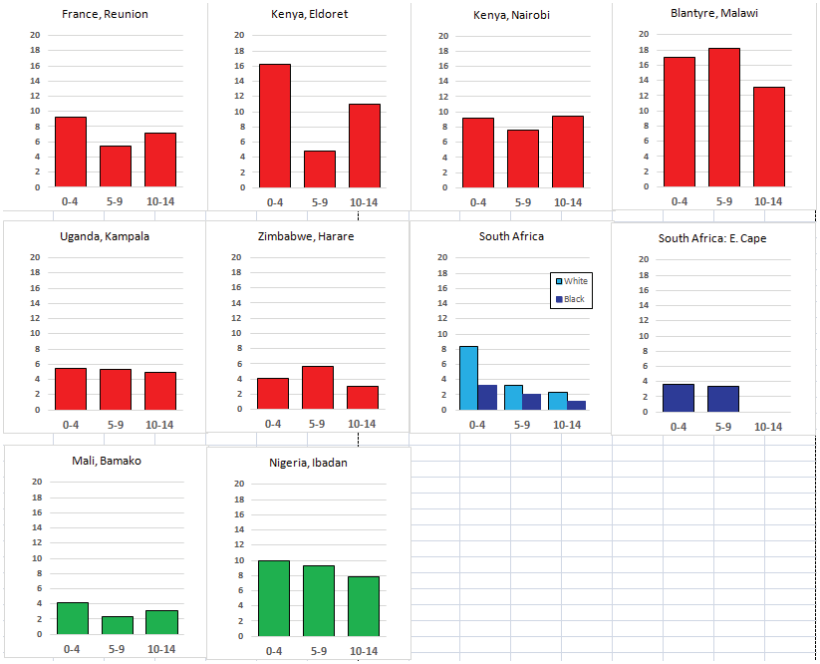
Age-specific histograms—rhabdomyosarcoma.

#### 8.9.2 Kaposi Sarcoma

**Table 8.9.2.**
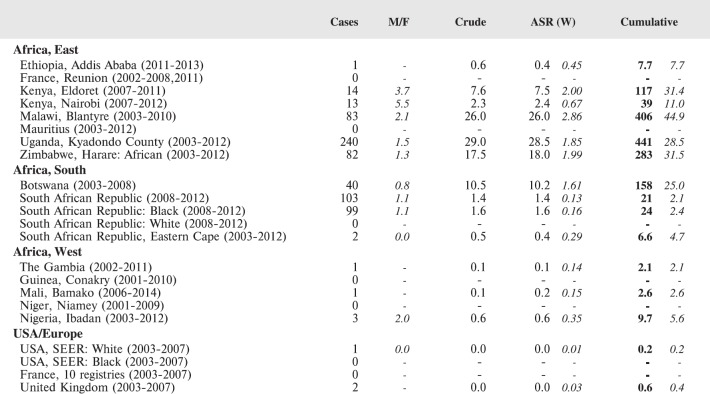
Crude, age-standardised (world) and cumulative (0–14) incidence rates (per million) and standard errors—Kaposi sarcoma.

**Figure 8.9.2a.**
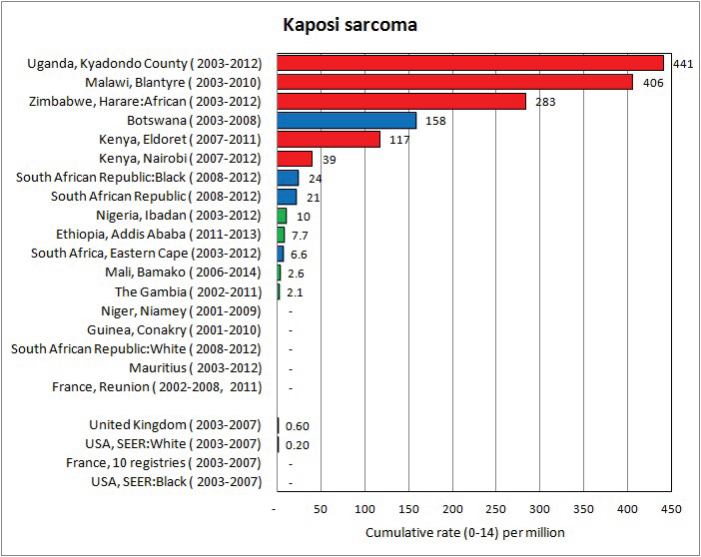
Cumulative (0–14) incidence rate—Kaposi sarcoma.

**Figure 8.9.2b.**
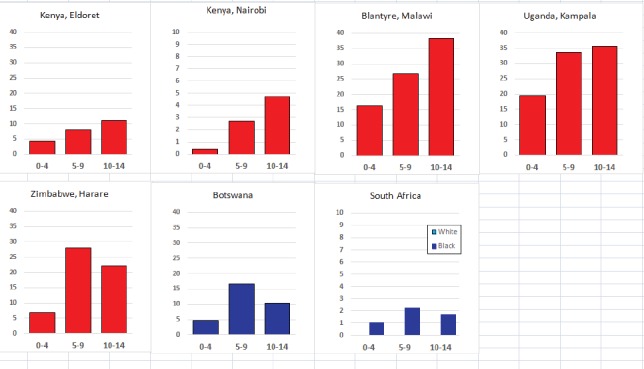
Age-specific histograms—Kaposi sarcoma.

Among white populations, soft-tissue sarcomas account for 4–8% of all childhood cancers and have a cumulative incidence of about 100–150 per million. Between two-thirds and three quarters are rhabdomyosarcomas, 10–20% are fibrosarcomas (including malignant fibrous histiocytoma and neurofibrosarcoma) and the remainder are other rare types. Among blacks in the United States, rhabdomyosarcoma has a similar incidence whereas fibrosarcoma is somewhat more common [92].

In Africa, incidence rates of rhabdomyosarcoma (Table 8.9.1, Figure 8.9.1a), although based on small numbers, show considerable variability, with relatively high rates in some centres, notably in East Africa.

Data from the period preceding the AIDS epidemic in sub-Saharan Africa give an indication of the incidence of childhood Kaposi sarcoma attributable to the endemic form of the disease. In Kampala (Uganda), the cumulative rate (per million) in 1960–1971 was 42.7, while in Bulawayo (Zimbabwe) in 1963–1977, it was 29.6 per 10^6^. With the advent of the epidemic of HIV-AIDS, very large increases in the incidence of KS were noted among children in East and Central Africa. In Kampala during 1993–1997, the rate was 771 per million, in Blantyre (Malawi) (1991–2001), it was 181 and among African residents of Harare (Zimbabwe) during 1990–1997, it was 147 per million [5]. In these two series, Kaposi sarcoma accounted for 37%, 16%, and 10% of all childhood cancers respectively. In Zambia over a similar period, the relative frequency was 19% [93]. In these early years of the epidemic of HIV-AIDS, childhood, Kaposi sarcoma was relatively frequent in young children (0–4 and 5–9), as a results of congenital IIV infection [94].

The great majority of the increase in incidence was related to the AIDS epidemic, which has been particularly severe in East and Central Africa. There seems to have been something of a decline in incidence in Kampala since then (Table 8.9.2), where adult rates of KS have certainly declined, along with reductions in prevalence of HIV and availability of antiretroviral therapy [94]. Nevertheless, KS remains the second most common cancer in children in Kampala (after BL), and it is still the principal cancer of children in Harare (2003–2012). Although very high rates of HIV infection are observed in Southern and, to a lesser extent, Western Africa, endemic KS has never been common in these areas, so childhood KS remains relatively rare.

It is interesting that in those areas with high rates of KS (as a result of HIV infection), such as Kampala, Harare, and Blantyre, the rates of childhood KS have declined in the last 10–15 years, and the age specific pattern has changed, so that it is now more frequent in older children (5–9 and 10–14) (Figure 9.5; [95, 96]. Early HIV diagnosis and a start of ART before advanced immunodeficiency develops has been shown to substantially reduce the risk of KS in HIV-positive children in South Africa [97]. Even before the onset of the AIDS epidemic, however, Kaposi sarcoma in childhood had very different clinical features from endemic Kaposi sarcoma of adults, and more resembled epidemic AIDS-related Kaposi sarcoma. Thus, it was often poly-lymphadenopathic, with either absent or sparse and anomalously sited skin lesions. Progression was rapid [98, 99].

### 8.10. Germ cell tumours

**Table 8.10.**
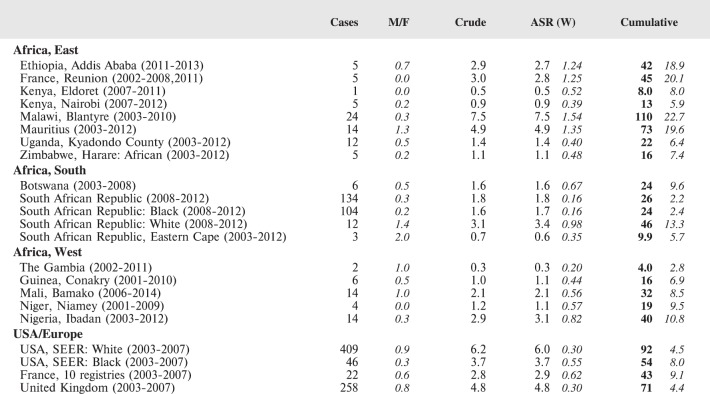
Crude, age-standardised (world) and cumulative (0–14) incidence rates (per million) and standard errors—germ cell tumours.

**Figure 8.10a.**
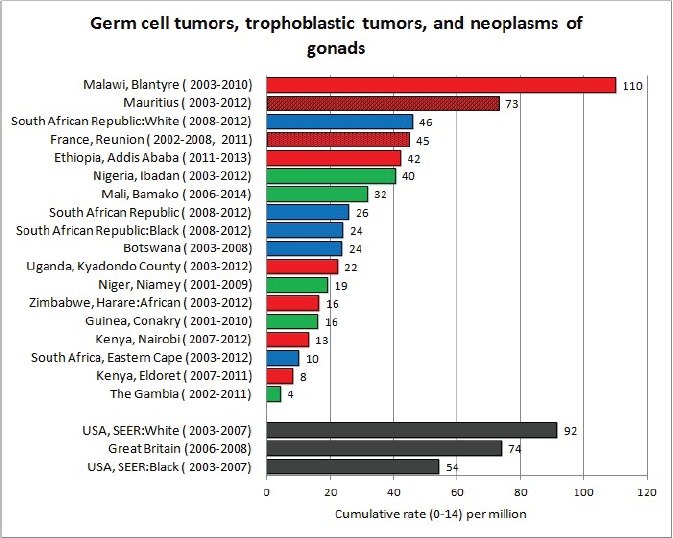
Cumulative (0–14) incidence rate—germ cell tumours.

**Figure 8.10b.**
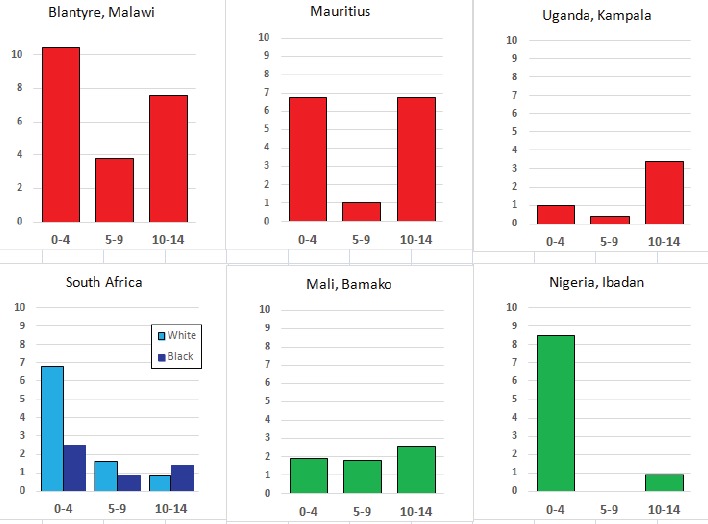
Age-specific histograms—germ cell tumours.

Germ-cell tumours generally account for less than 4% of all childhood cancers. Testicular tumours are rare in black children in the United States [15]; incidence rates for germ-cell tumours are about one third to one quarter those in white children [4]. These cancers are rare in all series of childhood cancers from Africa [100, 5, 101]. Table 8.10 shows the rates from the series in this monograph.

Only in Blantyre (Malawi) and in black children in RSA, there are sufficient cases for more detailed analysis. In Malawi, there were five cases in boys (only one of which was a testicular (yolk sac) tumour), and 19 in girls, 12 of which were ovarian. The remained were malignant extracranial and extragonadal germ cell tumours. In Rep. of South Africa, the great majority of germ cell tumours were extracranial and extragonadal (81/104); only 15 of the 104 cases (14.4%) were boys, and none of the tumours was gonadal (testicular), compared with 17/89 (19%) of the germ cell tumours in originating in the ovary in girls.

### 8.11. Carcinomas

**Table 8.11.**
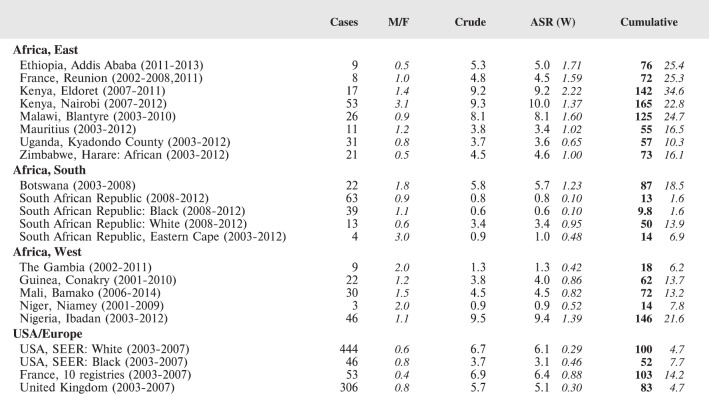
Crude, age-standardised (world) and cumulative (0–14) incidence rates (per million) and standard errors—carcinomas.

**Table 8.11a.** Numbers of carcinomas, as a percentage of all childhood cancer, and distribution by site.

	XI		XIa	XIb	XIc	XId	XIe	XIf(1)	XIf(2)	XIf(4)	XIf(8)	XIf(9)	XIf(10)	XIf(11)
	**ALL**		**Adrenal**	**Thyroid**	**Naso pharynx**	**Melanoma**	**Skin (other)**	**Salivary Gland**	**Large bowel**	**Lung**	**Bladder**	**Eye**	**Other sites**	**Unspecd**
Ethiopia, Addis Ababa (2011-2013)	9	(5.6%)	1	1	1	0	1	0	0	0	0	0	3	2
France, Reunion (2002-2008,2011)	8	(4.5%)	0	0	2	0	1	1	0	3	0	0	1	0
Kenya, Eldoret (2007-2011)	17	(6.0%)	0	0	5	1	3	0	1	0	0	0	3	4
Kenya, Nairobi (2007-2012)	53	(9.2%)	0	0	21	0	6	0	4	0	1	2	16	3
Malawi, Blantyre (2003-2010)	26	(2.6%)	0	1	1	0	4	4	0	0	1	3	2	10
Mauritius (2003-2012)	11	(4.1%)	0	1	1	0	0	3	3	0	1	0	1	1
Uganda, Kyadondo County (2003-2012)	31	(2.5%)	0	2	8	3	3	3	1	0	0	4	5	2
Zimbabwe, Harare: African (2003-2012)	21	(4.2%)	0	0	3	1	5	2	0	0	0	5	3	2
Botswana (2003-2008)	22	(8.2%)	0	2	3	2	3	0	0	0	0	3	8	1
South Africa (2008-2012)	63	(1.8%)	2	3	15	13	6	6	1	1	0	2	9	5
South Africa: Black (2008-2012)	39	(1.5%)	2	1	13	1	6	6	1	0	0	1	4	4
South Africa: White (2008-2012)	13	(3.8%)	0	1	0	11	0	0	0	1	0	0	0	0
South Africa, E. Cape (2003-2012)	4	(3.5%)	0	0	0	0	0	1	0	0	0	0	1	2
The Gambia (2002-2011)	9	(4.6%)	0	1	0	3	2	1	0	0	0	1	0	1
Guinea, Conakry (2001-2010)	22	(13.3%)	0	0	0	2	4	1	3	1	0	0	8	3
Mali, Bamako (2006-2014)	30	(3.7%)	0	1	0	0	5	0	1	2	2	4	12	3
Niger, Niamey (2001-2009)	3	(1.7%)	0	1	0	0	1	0	0	0	0	1	0	0
Nigeria, Ibadan (2003-2012)	46	(11.9%)	0	7	2	0	5	2	2	0	0	4	8	16
	427	(3.4%)	5	22	75	37	55	30	17	8	5	30	84	59
			1.2%	5.2%	17.6%	8.7%	12.9%	7.0%	4.0%	1.9%	1.2%	7.0%	19.7%	13.8%

**Figure 8.11a.**
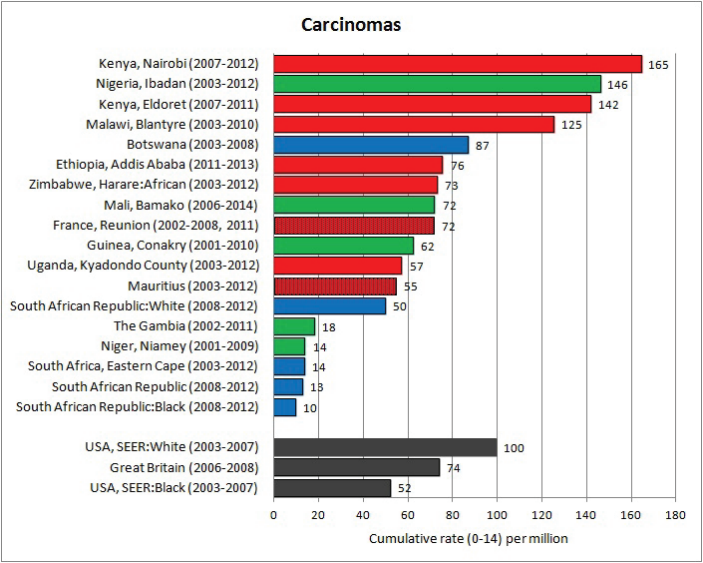
Cumulative (0–14) incidence rate—carcinomas.

**Figure 8.11b.**
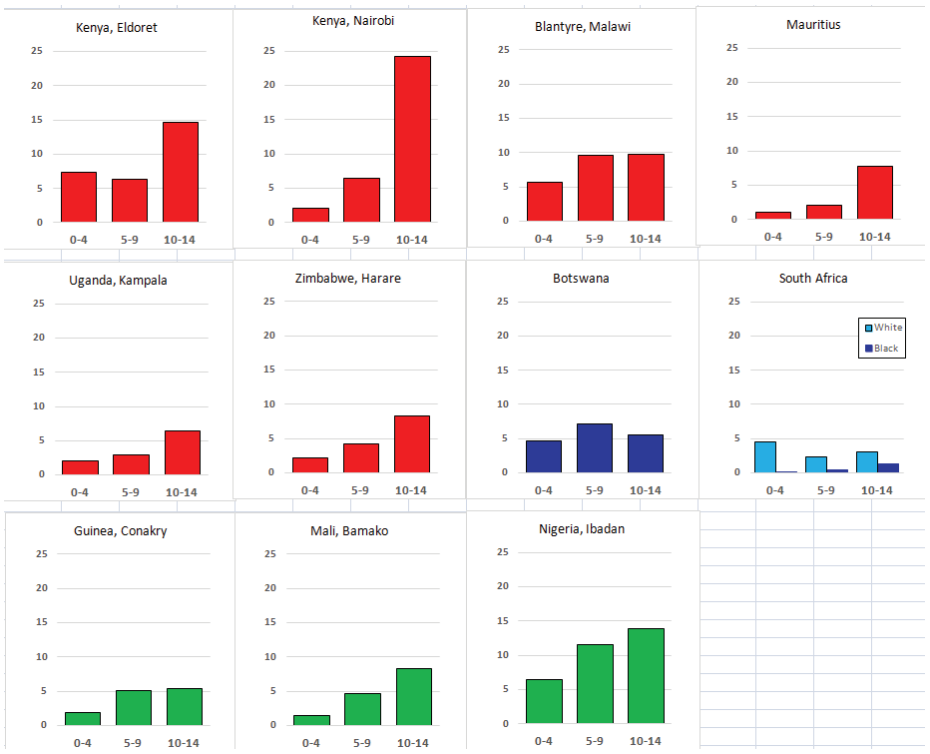
Age-specific histograms—carcinomas.

Carcinomas are not common in childhood. They comprise about 3.38% of childhood cancers in US white children, and 2.95% in the UK [4].

There is a wide variation in the series in this volume, from 1.5% of childhood cancers in RSA black children, to 13.7% in Conakry (Guinea). The incidence rates also show a wide range, with rates generally rather higher than in US black children. The highest incidence rates are observed in Nairobi and Eldoret (Kenya), Ibadan (Nigeria) and Blantyre (Malawi). Rates are generally highest in the 10–14 year age group (Figure 8.11b).

The most common carcinoma in African children overall (all series combined) is nasopharyngeal carcinoma (17.6%, Table 8.11a), as it is in Kenya (Nairobi and Eldoret), Uganda (Kampala), and RSA (black children). The relatively high incidence of nasopharyngeal cancers in Kenya – including childhood cases - was noted by Clifford and Beecher (1961) [102]. In the United States, black children had an incidence five times that in whites [107], and nasopharyngeal carcinoma has been reported to be the most frequent epithelial neoplasm in several case series (Ibadan, Nigeria [67], Uganda [99] and Zambia [92]).

Ascertainment of skin carcinoma is probably incomplete in most registries, but overall it is the second most frequent type of epithelial neoplasm. Malignant melanoma is less common – except in white children in RSA (11/13 carcinomas).

Squamous cell carcinoma of conjunctiva is relatively common in centres with high prevalence of HIV (and incidence of Kaposi sarcoma): this cancer comprises 24% of childhood carcinomas in Harare (Zimbabwe) and 12–14% in Uganda, Botswana and Malawi.

The thyroid is the most common site for carcinomas in children in many regions of the world [107], but such cancers appear to be rather rare in African children (except in Ibadan (Nigeria), where 15% of childhood carcinomas were thyroid tumours).

## Figures and Tables

**Table 4.** Total number of childhood cancers contributed by each African registry.

Cancer registry and time period	Number of cases
**Africa, East**	
Ethiopia, Addis Ababa (2011–2013)	160
France, Reunion (2002–2008, 2011)	178
Kenya, Eldoret (2007–2011)	282
Kenya, Nairobi (2007–2012)	577
Malawi, Blantyre (2003–2010)	986
Mauritius (2003–2012)	270
Uganda, Kyadondo County (2003–2012)	1254
Zimbabwe, Harare: African (2003–2012)	504
**Africa, South**	
Botswana (2003–2008)	267
South Africa (CCSG)^*^ (2008–2012)	3483
Black children	2583
White children	340
South African Republic, Eastern Cape (2003–2012)	113
**Africa, West**	
The Gambia (2002–2011)	194
Guinea, Conakry (2001–2010)	166
Mali, Bamako (2006–2014)	801
Niger, Niamey (2001–2009)	175
Nigeria, Ibadan (2003–2012)	387
^*^Childhood Cancer Study Group	

**Table 5.1.** International Classification of Childhood Cancer (ICCC-3).

Site group	ICD-O-3 histology (type)	ICD-O-2/3 site
I Leukaemias, myeloproliferative diseases, and myelodysplastic diseases		
(a) Lymphoid leukaemias	9820, 9823, 9826, 9827, 9831-9837, 9940, 9948	C000-C809
(b) Acute myeloid leukaemias	9840, 9861, 9866, 9867, 9870-9874, 9891, 9895-9897, 9910, 9920, 9931	C000-C809
(c) Chronic myeloproliferative diseases	9863, 9875, 9876, 9950, 9960-9964	C000-C809
(d) Myelodysplastic syndrome and other myeloproliferative diseases	9945, 9946, 9975, 9980, 9982-9987, 9989	C000-C809
(e) Unspecified and other specified leukaemias	9800, 9801, 9805, 9860, 9930	C000-C809
II Lymphomas and reticuloendothelial neoplasms		
(a) Hodgkin lymphomas	9650–9655, 9659, 9661–9665, 9667	C000-C809
(b) Non-Hodgkin lymphomas (except Burkitt lymphoma)	9591, 9670, 9671, 9673, 9675, 9678–9680, 9684, 9689–9691, 9695, 9698–9702, 9705, 9708, 9709, 9714, 9716–9719, 9727–9729, 9731–9734, 9760–9762, 9764–9769, 9970	C000-C809
(c) Burkitt lymphoma	9687	C000-C809
(d) Miscellaneous lymphoreticular neoplasms	9740–9742, 9750, 9754–9758	C000-C809
(e) Unspecified lymphomas	9590, 9596	C000-C809
III CNS and misc. intracranial and intraspinal neoplasms		
(a) Ependymomas and choroid plexus tumour	9383, 9390–9394	C000-C809
(b) Astrocytomas	9380	C723
	9384, 9400–9411, 9420, 9421–9424, 9440–9442	C000-C809
(c) Intracranial and intraspinal embryonal tumours	9470–9474, 9480, 9508	C000-C809
	9501–9504	C700-C729
(d) Other gliomas	9380	C700-C722, C724-C729, C751, C753
	9381, 9382, 9430, 9444, 9450, 9451, 9460	C000-C809
(e) Other specified intracranial and intraspinal neoplasms	8270–8281, 8300, 9350–9352, 9360–9362, 9412, 9413, 9492, 9493, 9505–9507, 9530–9539, 9582	C000-C809
(f) Unspecified intracranial and intraspinal neoplasms	8000–8005	C700-C729, C751-C753
IV Neuroblastoma and other peripheral nervous cell tumours		
(a) Neuroblastoma and ganglioneuroblastoma	9490, 9500	C000-C809
(b) Other peripheral nervous cell tumours	8680–8683, 8690–8693, 8700, 9520–9523	C000-C809
	9501–9504	C000-C699, C739-C768, C809
V Retinoblastoma	9510–9514	C000-C809
VI Renal tumours		
(a) Nephroblastoma and other non-epithelial renal tumours	8959, 8960, 8964–8967	C000-C809
	8963, 9364	C649
(b) Renal carcinomas	8010-8041, 8050-8075, 8082, 8120–8122, 8130–8141, 8143, 8155, 8190–8201, 8210, 8211, 8221–8231, 8240, 8241, 8244–8246, 8260–8263, 8290, 8310, 8320, 8323, 8401, 8430, 8440, 8480–8490, 8504, 8510, 8550, 8560–8576	C649
	8311, 8312, 8316–8319, 8361	C000-C809
(c) Unspecified malignant renal tumours	8000-8005	C649
VII Hepatic tumours		
(a) Hepatoblastoma	8970	C000-C809
(b) Hepatic carcinomas	8010–8041, 8050–8075, 8082, 8120–8122, 8140, 8141, 8143, 8155, 8190–8201, 8210, 8211, 8230, 8231, 8240, 8241, 8244–8246, 8260–8264, 8310, 8320, 8323, 8401, 8430, 8440, 8480–8490, 8504, 8510, 8550, 8560–8576	C220, C221
	8160–8180	C000-C809
(c) Unspecified malignant hepatic tumours	8000–8005	C220, C221
VIII Malignant bone tumours		
(a) Osteosarcomas	9180–9187, 9191–9195, 9200	C400-C419, C760-C768, C809
(b) Chondrosarcomas	9210, 9220, 9240	C400-C419, C760-C768, C809
	9221, 9230, 9241–9243	C000-C809
(c) Ewing tumour and related sarcomas of bone	9260	C400-C419, C760-C768, C809
	9363–9365	C400-C419
(d) Other specified malignant bone tumours	8810, 8811, 8823, 8830	C400-C419
	8812, 9250, 9261, 9262, 9270–9275, 9280–9282, 9290, 9300–9302, 9310–9312, 9320–9322, 9330, 9340–9342, 9370–9372	C000-C809
(e) Unspecified malignant bone tumours	8000–8005, 8800, 8801, 8803–8805	C400-C419
IX Soft tissue and other extraosseous sarcomas		
(a)Rhabdomyosarcomas	8900–8905, 8910, 8912, 8920, 8991	C000-C809
(b) Fibrosarcomas, peripheral nerve sheath tumours, and other fibrous neoplasms	8810, 8811, 8813–8815, 8821, 8823, 8834–8835	C000-C399, C440-C768, C809
	8820, 8822, 8824–8827, 9150, 9160, 9491, 9540–9571, 9580	C000-C809
(c) Kaposi sarcoma	9140	C000-C809
(d) Other specified soft tissue sarcomas	8587, 8710–8713, 8806, 8831–8833, 8836, 8840–8842, 8850–8858, 8860–8862, 8870, 8880, 8881, 8890–8898, 8921, 8982, 8990, 9040–9044, 9120–9125, 9130–9133, 9135, 9136, 9141, 9142, 9161, 9170–9175, 9231, 9251, 9252, 9373, 9581	C000-C809
	8830	C000-C399, C440-C768, C809
	8963	C000-C639, C659-C699, C739-C768, C809
	9180, 9210, 9220, 9240	C490-C499
	9260	C000-C399, C470-C759
	9364	C000-C399, C470-C639, C659-C699, C739-C768, C809
	9365	C000-C399, C470-C639, C659-C768, C809
(e) Unspecified soft tissue sarcomas	8800–8805	C000-C399, C440-C768, C809
X Germ cell tumours, trophoblastic tumours, and neoplasms of gonads		
(a) Intracranial and intraspinal germ cell tumours	9060–9065, 9070–9072, 9080–9085, 9100, 9101	C700-C729, C751-C753
(b) Malignant extracranial and extragonadal germ cell tumours	9060–9065, 9070–9072, 9080–9085, 9100–9105	C000-C559, C570-C619, C630-C699, C739-C750, C754-C768, C809
(c) Malignant gonadal germ cell tumours	9060–9065, 9070–9073, 9080–9085, 9090, 9091, 9100, 9101	C569, C620-C629
(d) Gonadal carcinomas	8010–8041, 8050–8075, 8082, 8120–8122, 8130–8141, 8143, 8190–8201, 8210, 8211, 8221–8241, 8244–8246, 8260–8263, 8290, 8310, 8313, 8320, 8323, 8380–8384, 8430, 8440, 8480–8490, 8504, 8510, 8550, 8560–8573, 9000, 9014, 9015	C569, C620-C629
	8441–8444, 8450, 8451, 8460–8473	C000-C809
(e) Other and unspecified malignant gonadal tumours	8590–8671	C000-C809
	8000–8005	C569, C620-C629
XI Other malignant epithelial neoplasms and malignant melanomas		
(a) Adrenocortical carcinomas	8370–8375	C000-C809
(b) Thyroid carcinomas	8010–8041, 8050–8075, 8082, 8120–8122, 8130–8141, 8190, 8200, 8201, 8211, 8230, 8231, 8244–8246, 8260–8263, 8290, 8310, 8320, 8323, 8430, 8440, 8480, 8481, 8510, 8560–8573	C739
	8330–8337, 8340–8347, 8350	C000-C809
(c) Nasopharyngeal carcinomas	8010–8041, 8050–8075, 8082, 8083, 8120–8122, 8130–8141, 8190, 8200, 8201, 8211, 8230, 8231, 8244–8246, 8260–8263, 8290, 8310, 8320, 8323, 8430, 8440, 8480, 8481, 8500–8576	C110-C119
(d) Malignant melanomas	8720–8780, 8790	C000-C809
(e) Skin carcinomas	8010–8041, 8050–8075, 8078, 8082, 8090–8110, 8140, 8143, 8147, 8190, 8200, 8240, 8246, 8247, 8260, 8310, 8320, 8323, 8390–8420, 8430, 8480, 8542, 8560, 8570–8573, 8940, 8941	C440-C449
(f) Other and unspecified carcinomas	8010–8084, 8120–8157, 8190–8264, 8290, 8310, 8313–8315, 8320–8325, 8360, 8380–8384, 8430–8440, 8452–8454, 8480–8586, 8588–8589, 8940, 8941, 8983, 9000, 9010–9016, 9020, 9030	C000-C109, C129-C218, C239-C399, C480-C488, C500-C559, C570-C619, C630-C639, C659-C729, C750-C768, C809
XII Other and unspecified malignant neoplasms		
(a) Other specified malignant tumours	8930–8936, 8950, 8951, 8971–8981, 9050–9055, 9110	C000-C809
	9363	C000-C399, C470-C759
(b) Other unspecified malignant tumours	8000–8005	C000-C218, C239-C399, C420-C559, C570-C619, C630-C639, C659-C699, C739-C750, C754-C809

**Table 5.2.**
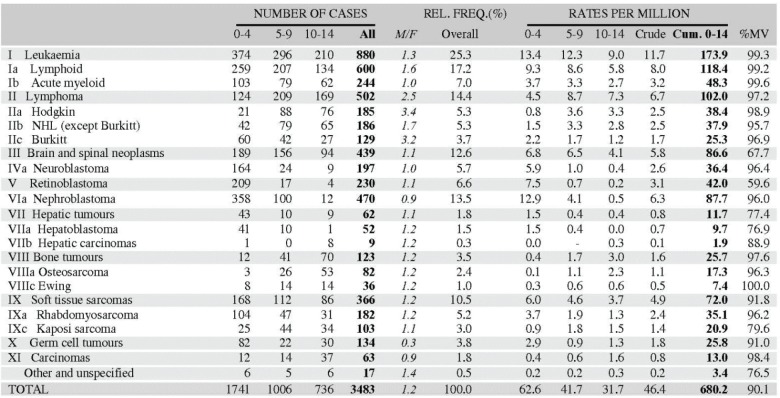
Table of incidence rates from one registry.

**Table 5.3.**
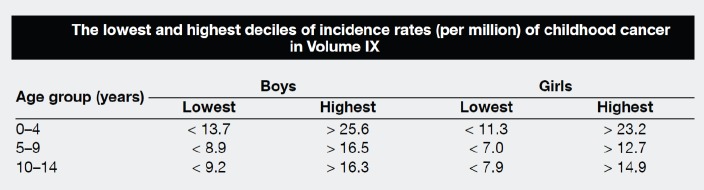
The lowest and highest deciles of incidence rates (per million) of childhood cancer in Vol IX.

**Table 5.4.**
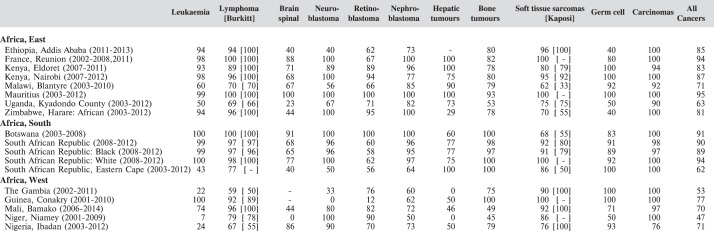
Percentage of microscopically verified cases – both sexes.

## 10. Abbreviations

AFCRN: African Cancer Registry Network
DCO: Death certificate only (when a cancer case can only be identified on a death certificate.)
IACR: International Association of Cancer Registries
IARC: International Agency for Research on Cancer
ICD-O: International Classification of Diseases for Oncology
KS: Kaposi sarcoma
LMIC: Low and Middle Income Countries
NCD: Non-communicable disease
SSA: Sub Saharan Africa
WHO: World Health Organisation
CI5: Cancer Incidence in Five Continents
RSA: Republic of South Africa
ASR: Age-standardised rate

## Notes

1 AFRCN acknowledges the generous contributions of the following organisations Doris Duke Charitable Foundation Glaxo-Smith-Kline (Oncology) F. Hoffman-La Roche Ltd. International Union for Cancer Control (UICC) American Cancer Society (ACS)

## References

[1] Stiller CA, Parkin DM (1996). Geographic and ethnic variations in the incidence of childhood cancer. Br Med Bull.

[2] Gakunga R, Parkin DM, African Cancer Registry N (2015). Cancer registries in Africa 2014: a survey of operational features and uses in cancer control planning. Int J Cancer.

[3] Steliarova-Foucher E, Colombet M (2017). International incidence of childhood cancer, 2001-10: a population-based registry study. Lancet Oncol.

[4] Parkin DM, Kramárová E, Draper GJ (1998). International incidence of childhood cancer, vol. II.

[5] Parkin DM FJ, Hamdi-Chérif M, Sitas F (2003). Cancer in Africa: epidemiology and prevention.

[6] Fritz A, Percy C, Jack A (2000). International classification of diseases for oncology (ICD-O-3).

[7] Ferlay J, Burkhard C, Whelan S (2005). Check and conversion programs for cancer registries (IARC Technical Report No. 42).

[8] Steliarova-Foucher E, Stiller C, Lacour B (2005). International classification of childhood cancer. Cancer.

[9] Boyle P, Parkin DM (1991). Cancer registration: principles and methods Statistical methods for registries. IARC Sci Publ.

[10] Forman D BF, Brewster DH, Mbalawa CG (2013). Cancer incidence in five continents.

[11] Bray F, Parkin DM (2009). Evaluation of data quality in the cancer registry: principles and methods Part I: comparability, validity and timeliness. Eur J Cancer.

[12] Coebergh JW, Reedijk AM, de Vries E (2006). Leukaemia incidence and survival in children and adolescents in Europe during 1978-1997 Report from the automated childhood cancer information system project. Eur J Cancer.

[13] Fleming AF (1988). Possible aetiological factors in leukaemias in Africa. Leuk Res.

[14] Brown WC, Doll R (1961). Leukaemia in childhood and young adult life. BMJ.

[15] Miller RW, Mulvihill JJ, Miller RW, Fraumeni JF (1977). Ethnic differences in cancer occurrence: genetic and environmental influences with particular reference to neuroblastoma. Genetics of Human Cancer.

[16] Ries LAG SM, Gurney JG, Linet M (1999). Cancer incidence and survival among children and adolescents: United States SEER program 1975-1995.

[17] Stiller CA, McKinney PA, Bunch KJ (1991). Childhood cancer and ethnic group in Britain: a United Kingdom children’s Cancer Study Group (UKCCSG) study. Br J Cancer.

[18] Davis S, Rogers MA, Pendergrass TW (1987). The incidence and epidemiologic characteristics of neuroblastoma in the United States. Am J Epidemiol.

[19] Hesseling PB, Hartley P, Zietsman L (2004). Incidence of acute lymphoblastic leukaemia in white and coloured children in the Western Cape. S Afr Med J.

[20] Sayers GM, Rip MR, Jacobs P (1992). Epidemiology of acute leukaemia in the Cape Province of South Africa. Leuk Res.

[21] Fleming AF (1993). Leukaemias in Africa. Leukemia.

[22] Stiller C, Parkin D (1990). International variations in the incidence of childhood renal tumours. Br J Cancer.

[23] Clavel J, Steliarova-Foucher E, Berger C (2006). Hodgkin’s disease incidence and survival in European children and adolescents (1978-1997): report from the automated cancer information system project. Eur J Cancer.

[24] Levy LM (1988). Hodgkin’s disease in black Zimbabweans A study of epidemiologic, histologic, and clinical features. Cancer.

[25] Glaser SL (1990). Hodgkin’s disease in black populations: a review of the epidemiologic literature. Semin Oncol.

[26] Stefan DC, Stones D, Dippenaar A (2009). Ethnicity and characteristics of Hodgkin lymphoma in children. Pediatr Blood Cancer.

[27] Glaser SL, Lin RJ, Stewart SL (1997). Epstein-Barr virus-associated Hodgkin’s disease: epidemiologic characteristics in international data. Int J Cancer.

[28] Leoncini L, Spina D, Nyong’o A (1996). Neoplastic cells of Hodgkin’s disease show differences in EBV expression between Kenya and Italy. Int J Cancer.

[29] Weinreb M, Day P, Niggli F (1996). The role of Epstein-Barr virus in Hodgkin’s disease from different geographical areas. Arch Dis Childhood.

[30] Wright DH (1967). The epidemiology of Burkitt’s tumour. Cancer Res.

[31] Burkitt DP (1969). Etiology of Burkitt’s lymphoma—an alternative hypothesis to a vectored virus. Journal of the National Cancer Institute.

[32] O’Conor GT (1970). Persistent immunologic stimulation as a factor in oncogenesis, with special reference to Burkitt’s tumor. Am J Med.

[33] Burkitt D, Wright D (1966). Geographical and tribal distribution of the African lymphoma in Uganda. BMJ.

[34] Edington G, Maclean CM (1964). Incidence of the Burkitt tumour in Ibadan, western Nigeria. BMJ.

[35] IARC (2012). Biological agents: a review of human carcinogens.

[36] Kelly GL, Rickinson AB (2007). Burkitt lymphoma: revisiting the pathogenesis of a virus-associated malignancy. ASH Education Program Book.

[37] Carbone A, Gloghini A, Dotti G (2008). EBV-associated lymphoproliferative disorders: classification and treatment. Oncologist.

[38] Geser A, De Thé G, Lenoir G (1982). Final case reporting from the ugandan prospective study of the relationship between ebv and burktit’s lymphoma. Int J Cancer.

[39] IARC (1997). Epstein-barr virus and Kaposi’s sarcoma herpesvirus/Human herpesvirus.

[40] Rochford R, Cannon MJ, Moormann AM (2005). Endemic Burkitt’s lymphoma: a polymicrobial disease?. Nature Rev Microbiol.

[41] Morrow RH, Lenoir GM, O’Conor GT, Olweny CLM (1985). Epidemiological evidence for the role of falciparum malaria in the pathogenesis of Burkitt’s lymphoma.

[42] Geser A, Brubaker G, Draper CC (1989). Effect of a malaria suppression program on the incidence of African Burkitt’s lymphoma. Am J Epidemiol.

[43] Carpenter LM, Newton R, Casabonne D (2008). Antibodies against malaria and Epstein–Barr virus in childhood Burkitt lymphoma: a case–control study in Uganda. Int J Cancer.

[44] Mutalima N, Molyneux E, Jaffe H (2008). Associations between Burkitt lymphoma among children in Malawi and infection with HIV, EBV and malaria: results from a case-control study. PLoS One.

[45] Asito AS, Piriou E, Odada PS (2010). Elevated anti-Zta IgG levels and EBV viral load are associated with site of tumor presentation in endemic Burkitt’s lymphoma patients: a case control study. Infect Agent Cancer.

[46] Guech-Ongey M, Yagi M, Palacpac NMQ (2012). Antibodies reactive to Plasmodium falciparum serine repeat antigen in children with Burkitt lymphoma from Ghana. Int J Cancer.

[47] IARC (2014). Malaria and Some Polyomaviruses (SV40, BK, JC, and Merkel Cell Viruses). IARC monographs on the evaluation of carcinogenic risks to humans.

[48] Parkin D, Garcia-Giannoli H, Raphael M (2000). Non-Hodgkin lymphoma in Uganda: a case–control study. Aids.

[49] Otieno MW, Remick SC, Whalen C (2001). Adult Burkitt’s lymphoma in patients with and without human immunodeficiency virus infection in Kenya. Int J Cancer.

[50] Dave SS, Fu K, Wright GW (2006). Molecular diagnosis of Burkitt’s lymphoma. NEngl J Med.

[51] Hummel M, Bentink S, Berger H (2006). A biologic definition of Burkitt’s lymphoma from transcriptional and genomic profiling. N Engl J Med.

[52] De Falco G, Ambrosio MR, Fuligni F (2015). Burkitt lymphoma beyond MYC translocation: N-MYC and DNA methyltransferases dysregulation. BMC Cancer.

[53] Piccaluga PP, De Falco G, Kustagi M (2011). Gene expression analysis uncovers similarity and differences among Burkitt lymphoma subtypes. Blood.

[54] Abate F, Ambrosio MR, Mundo L Distinct viral and mutational spectrum of endemic burkitt lymphoma. PLoS Pathog.

[55] Ambrosio MR, Piccaluga PP, Ponzoni M (2012). The alteration of lipid metabolism in Burkitt lymphoma identifies a novel marker: adipophilin. PLoS ONE.

[56] Schmitz R, Young RM, Ceribelli M (2012). Burkitt lymphoma pathogenesis and therapeutic targets from structural and functional genomics. Nature.

[57] Ambrosio MR, Navari M, Di Lisio L (2014). The Epstein Barr-encoded BART-6-3p microRNA affects regulation of cell growth and immuno response in Burkitt lymphoma. Infect Agent Cancer.

[58] Amato T, Abate F, Piccaluga P (2016). Clonality analysis of immunoglobulin gene rearrangement by next-generation sequencing in endemic Burkitt lymphoma suggests antigen drive activation of BCR as opposed to sporadic Burkitt lymphoma. Am J Clin Pathol.

[59] Jaffe ES, Harris NL, Stein H (2001). Pathology and genetics of tumours of the haematopoietic and lymphoid tissues.

[60] Iscovich J, Parkin D (1997). Risk of cancer in migrants and their descendants in Israel: I. Leukaemias and lymphomas. Int J Cancer.

[61] O’Conor GT, Davies J (1960). Malignant tumors in African children: with special reference to malignant lymphoma. J Pediatr.

[62] Kung’u A (1984). Childhood cancers in Kenya A histopathological and epidemiological study. East Afr Med J.

[63] Makata AM, Toriyama K, Kamidigo NO (1996). The pattern of pediatric solid malignant tumors in western Kenya, east Africa, 1979–1994: an analysis based on histopathologic study. Am J Trop Med Hygiene.

[64] Mwanda O (1999). Cancers in children younger than age 16 years in Kenya. East Afr Med J.

[65] Miller RW (1989). No neuroblastoma in Zaire. Lancet.

[66] Parkin DM, Stiller CA, Bieber CA (1988). International incidence of childhood cancer.

[67] Williams AO (1975). Tumors of childhood in Ibadan, Nigeria. Cancer.

[68] Traoré F, Eshun F, Togo B (2016). Neuroblastoma in Africa: a survey by the Franco-African pediatric oncology group. J Global Oncol.

[69] Heck JE, Ritz B, Hung RJ (2009). The epidemiology of neuroblastoma: a review. Paediatr Perinat Epidemiol.

[70] French AE, Grant R, Weitzman S (2003). Folic acid food fortification is associated with a decline in neuroblastoma. Clin Pharmacol Ther.

[71] Chow EJ, Friedman DL, Mueller BA (2007). Maternal and perinatal characteristics in relation to neuroblastoma. Cancer.

[72] Draper GJ, Sanders BM, Brownbill PA (1992). Patterns of risk of hereditary retinoblastoma and applications to genetic counselling. Br J Cancer.

[73] Waddell KM, Kagame K, Ndamira A (2015). Clinical features and survival among children with retinoblastoma in Uganda. Br J Ophthalmol.

[74] Wessels G, Hesseling P (1996). Unusual distribution of childhood cancer in Namibia. Pediatr Hematol Oncol.

[75] Traore F, Togo B, Sylla F (2003). Retinoblastoma: inventory in Mali and program to develop early diagnosis, treatments and rehabilitation. Bull Cancer.

[76] Breslow NOA, Beckwith JB, Moksness J (1994). Ethnic variation in the incidence, diagnosis, prognosis, and follow-up of children with Wilms’ tumor. J Natl Cancer Inst.

[77] Olshan AF, Breslow NE, Falletta JM (1993). Risk factors for Wilms tumor: report from the National Wilms Tumor Study. Cancer.

[78] Reynolds P, Urayama KY, Von Behren J (2004). Birth characteristics and hepatoblastoma risk in young children. Cancer.

[79] Spector LG, Birch J (2012). The epidemiology of hepatoblastoma. Pediatr Blood Cancer.

[80] Cameron H, Warwick G (1977). Primary cancer of the liver in Kenyan children. Br J Cancer.

[81] Moore S, Hesseling P, Wessels G (1997). Hepatocellular carcinoma in children. Pediatr Surg Int.

[82] Edmunds WJ, Medley GF, Nokes DJ (1993). The influence of age on the development of the hepatitis B carrier state. Proc Biol Sci.

[83] IARC (2002). Some traditional herbal medicines, some mycotoxins, naphthalene and styrene.

[84] Turner PC, Sylla A, Kuang SY (2005). Absence of TP53 codon 249 mutations in young Guinean children with high aflatoxin exposure. Cancer Epidemiol Biomark Prevent.

[85] Chiang CJ, Yang YW, You SL (2013). Thirty-year outcomes of the national hepatitis B immunization program in Taiwan. JAMA.

[86] Moore S, Davidson A, Hadley G (2008). Malignant liver tumors in South African children: a national audit. World J Surg.

[87] Johnson LC (1953). A general theory of bone tumours. Bull NY Acad Med.

[88] Fraumeni JF, Glass AG (1970). Rarity of Ewing’s sarcoma among U.S. Negro children. Lancet.

[89] Glass AG, Fraumeni JF (1970). Epidemiology of bone cancer in children. J Natl Cancer Instit.

[90] Parkin D, Stiller C, Nectoux J (1993). International variations in the incidence of childhood bone tumours. Int J Cancer.

[91] Beck R, Monument MJ, Watkins WS (2012). EWS/FLI-responsive GGAA microsatellites exhibit polymorphic differences between European and African populations. Cancer Genet.

[92] Stiller C, Parkin D (1994). International variations in the incidence of childhood soft-tissue sarcomas. Paediatr Perinatal Epidemiol.

[93] Chintu C, Athale UH, Patil P (1995). Childhood cancers in Zambia before and after the HIV epidemic. Arch Dis Child.

[94] Parkin DM, Wabinga H, Nambooze S (1999). AIDS-related cancers in Africa: maturation of the epidemic in Uganda. AIDS.

[95] Wabinga HR, Nambooze S, Amulen PM (2014). Trends in the incidence of cancer in Kampala, Uganda 1991–2010. Int J Cancer.

[96] Chokunonga E, Borok MZ, Chirenje ZM (2013). Trends in the incidence of cancer in the black population of Harare, Zimbabwe 1991-2010. Int J Cancer.

[97] Bohlius J, Maxwell N, Spoerri A (2016). Incidence of AIDS-defining and other cancers in HIV-positive children in South Africa: record linkage study. Pediatr Infect Dis J.

[98] Slavin G, Cameron HM, Forbes C (1970). Kaposi’s sarcoma in East African children: a report of 51 cases. J Pathol.

[99] Olweny C, Kaddumukasa A, Atine I (1976). Childhood Kaposi’s sarcoma: clinical features and therapy. Br J Cancer.

[100] Davies JNP, Templeton AC (1973). Childhood tumours. Tumours in a tropical country Recent results in cancer research (No.41).

[101] Babatunde T, Akang E, Ogun G (2015). Pattern of childhood cancer in University College Hospital, Ibadan during 1991–2010 and comparison with the previous three decades. Paediatr Int Child Health.

[102] Clifford P, Beecher J (1964). Nasopharyngeal cancer in Kenya: clinical and environmental aspects. BrJ Cancer.

[103] (1995). Epidemiology of childhood cancer in Africa. Int J Pediatr Hematol Oncol.

[104] Carlsen NL (1986). Epidemiological investigations on neuroblastomas in Denmark 1943-1980. Br J Cancer.

[105] Kramárová E, Stiller CA, Ferlay J (1996). The international classification of childhood cancer.

[106] Russell HV SJ, Nuchtern JG (2010). Epidemiology, pathogenesis, and pathology of neuroblastoma. http://cursoenarm.net/UPTODATE/contents/mobipreview.htm?36/46/37600?source=see_link.

[107] Stiller C (1994). International variations in the incidence of childhood carcinomas. Cancer Epidemiol Biomark Prevent.

[108] Stiller C (1998). What causes Hodgkin’s disease in children?. Eur J Cancer.

